# Sulfur-Phenolate Exchange As a Fluorine-Free Approach to S(VI) Exchange Chemistry on Sulfonyl Moieties

**DOI:** 10.1021/acs.orglett.2c03421

**Published:** 2022-11-16

**Authors:** Alyssa F.J. van den Boom, Muthusamy Subramaniam, Han Zuilhof

**Affiliations:** † Laboratory of Organic Chemistry, Wageningen University, Stippeneng 4, Wageningen 6708WE, The Netherlands; ‡ School of Pharmaceutical Science and Technology, Tianjin University, 92 Weijin Road, Tianjin 300072, China; § Department of Chemical and Materials Engineering, Faculty of Engineering, King Abdulaziz University, Jeddah 21589, Saudi Arabia

## Abstract

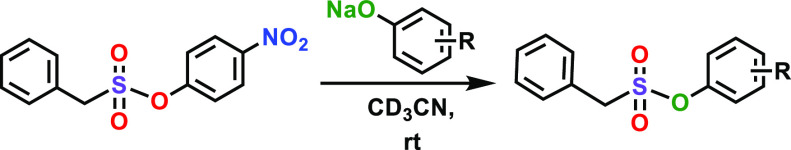

SuFEx chemistry has recently evolved as next-generation click chemistry. However, in most SuFEx syntheses, additional reagents/catalysts and carefully controlled conditions are still needed. Here, we aim to further generalize S(VI) exchange chemistry, using 4-nitrophenyl phenylmethanesulfonate as example, in which the nitrophenolate group is exchanged for a wide range of (substituted) phenols and alkyl alcohols. Quantitative yields were reached within 10 min under ambient conditions and required only filtering through silica as workup.

Over the past few decades, an ever-growing toolbox of click reactions has been developed, which has revolutionized the molecular sciences. Well-known examples are, for example, the copper(I)-catalyzed alkyne–azide cycloaddition, strain-promoted variants thereof, or thiol–ene radical additions.^1,2^ One of the most recent additions is the sulfur fluoride exchange (SuFEx) reaction, in which an S–F bond is replaced by an S–O or S–N bond.^3^ This reaction is both facile and high yielding, and has been shown to be highly useful for fields from medicinal chemistry to polymer chemistry.^4−8^ The original scope of the reaction was focused on silyl-protected phenols^9−13^ and (unprotected) amines.^14,15^ More recent extensions opened this up and allowed for the first intrinsically enantiospecific click reaction, namely the catalyst-free reaction of (sodium) phenolates to chiral sulfonimidoyl fluorides.^16,17^ Furthermore, addition of hexamethyl disilazane and base as catalysts recently extended the scope to saturated alcohols,^18^ BF_3_ catalysis allowed coupling of terminal alkynes,^19^ and the use of 1-hydroxybenzotriazole (HOBt) together with TMDS and DIPEA allowed the formation of sulfonamides.^20^

While SuFEx chemistry is generally considered as fast and easy, the addition of extra reagents, catalysts, or use of an inert atmosphere is typically needed to reach high yields. Examples of additional reagents or catalysts used in the literature include BTMG (yield without 77%, yield with >99%)^18^ [Ph_3_P = N–PPh_3_]^+^[HF_2_]^−^ (yield 99%),^9^ and tris(dimethylamimo)sulfonium bifluoride.^11^ Finally, the involvement of fluorine does provide the S–F reagents with a remarkable combination of stability and specific reactivity, but makes the SuFEx chemistry less attractive from both an environmental and industrial point of view, due to the temporary formation of surface-etching fluoride anions, the toxicity of fluoride anions, and government regulations that increasingly restrict the use of fluorine-containing chemicals. Evidently, there is need for a catalyst-free, rapid, high-yielding, and easy-to-handle S(VI) exchange chemistry that does not involve fluorine. Some important steps have already been taken in this direction, by the synthesis of 1-sulfonimidoyl- and 1-sulfamimidoyl-3-methylimidazolium derivatives that can be used to produce sulfonimidamides and imidosulfuric diamides,^21^ and the use of sulfonyl-triazoles for the synthesis of sulfonyl esters.^22^

In this work, we hope to expand upon these first steps, by presenting a generalized, fluorine-free version of S(VI) exchange chemistry—a Sulfur Phenolate Exchange reaction (SuPhenEx)—for SO_2_X moieties, in which nitrophenolate is the leaving group instead of fluoride. This approach is similar to previous studies on sulfonimidoyl moieties.^17^ Using 4-nitrophenyl phenylmethanesulfonate (**1**) as the starting material, we produce a wide range of products via a simple, uncatalyzed exchange reaction with a large selection of (natural) phenols and alkyl alcohols. The starting material for all reactions described hereafter, i.e., 4-nitrophenyl phenylmethanesulfonate (**1**), was prepared on a multigram scale from phenylmethanesulfonyl chloride and 4-nitrophenol (Figure 1). The crystalline material showed good stability for multiple weeks under ambient conditions, while a solution of **1** in 20% DMSO in water was stable for at least 9 days without signs of degradation, clearly demonstrating the high stability and easy-to-handle nature of the starting material **1**, which allows bulk production and long-term storage. Compound **1** was then used to generate a large range of S(VI) exchange products via a phenolate exchange reaction (Table 1).

**Figure 1:**
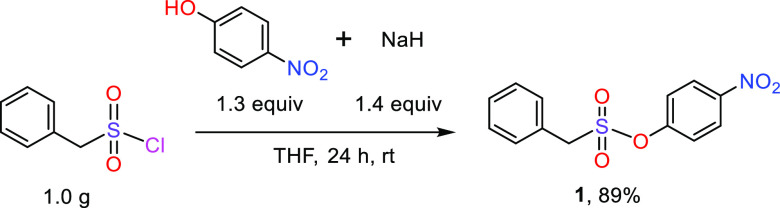
Synthesis of the starting compound, 4-nitrophenyl phenylmethanesulfonate (**1**).

**Table 1:**
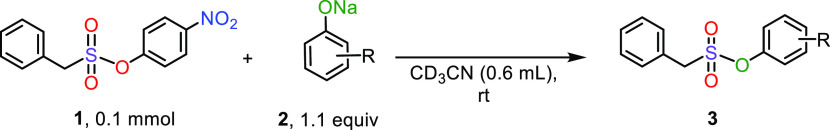

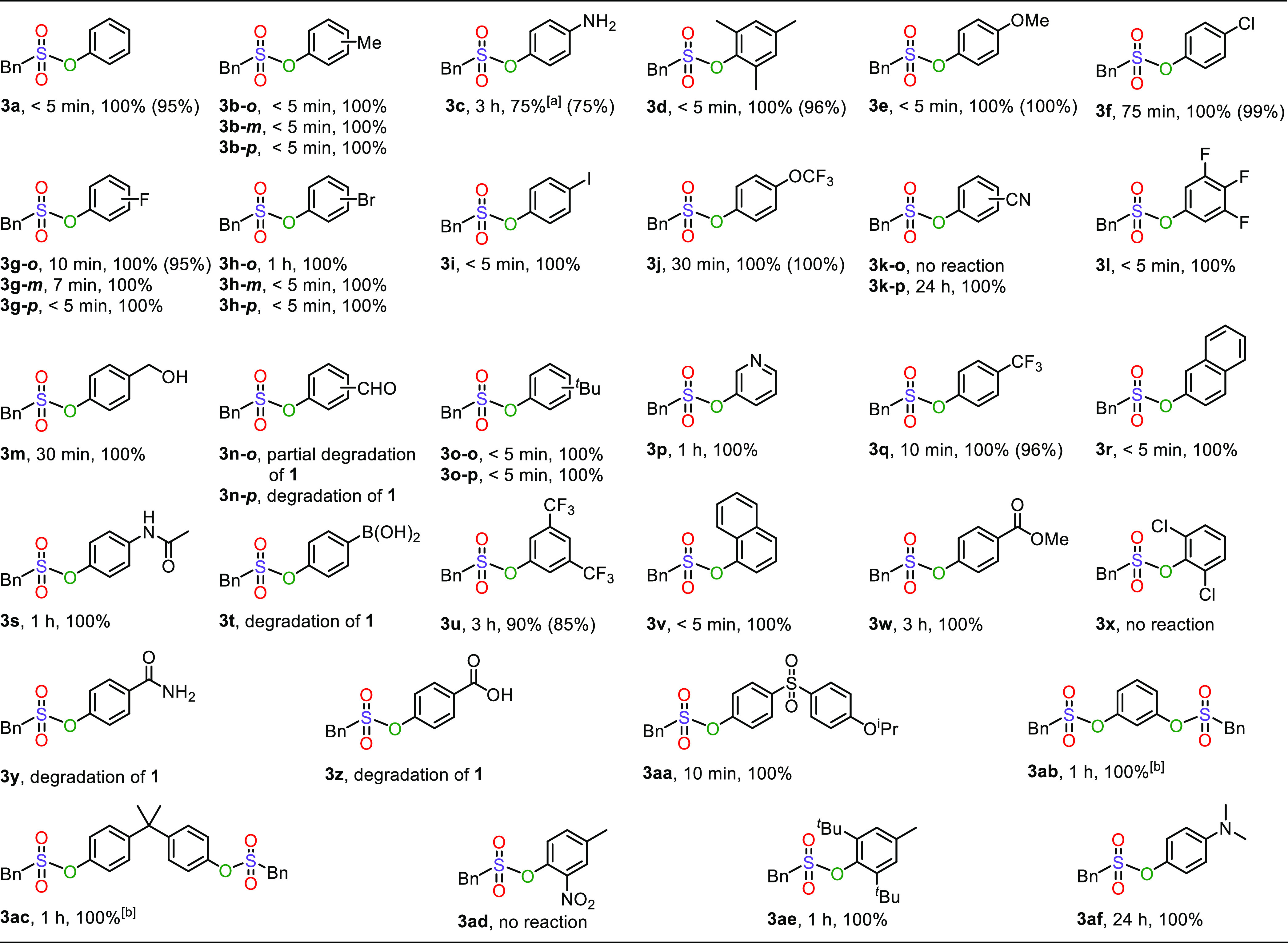
Reaction Times and Yields for the Exchange Reaction of **1** with a Range of Substrates^c^

a 75% yield for addition *via* the hydroxyl moiety, 25% yield for addition *via* the amine moiety.

b 2.1 equiv. of NaH, 2.1 equiv. of **1**, 1 equiv. of phenol.

c Yield was determined by NMR measurements, after filtering through a short silica plug. For several compounds, an isolated yield was also determined (yield in brackets).

This reaction hinges on the strong electron-withdrawing nature of the *p*-NO_2_ group on the nitrophenolate, which makes it an excellent leaving group in the exchange reaction. As a small added benefit, the strong orange color of the displaced nitrophenolate allows rough tracking of the reaction progress by eye. The exchange reaction does also work starting from less electron-withdrawing groups, like 4-CN or 4-Cl, yet the use of these starting materials drastically increases the reaction time; the conversion to product **3a**, which takes <5 min using **1**, takes 20 min using **3k**–**p** or 40 min using **3f** as a starting material. Still, the conversions remained clean, with typically 85–95% isolated yield and no byproducts.

As can be seen in Table 1, most products were formed with excellent yields (100%, as determined by NMR) after just 5 min of reaction time. Previous procedures to produce already-described products from Table 1 involved stirring for 1–24 h, in dry solvents, on an ice bath, under an inert atmosphere, using phenylmethanesulfonyl chloride as the starting material.^23^ Here, we simply stir at room temperature under ambient atmosphere, using nondried deuterated acetonitrile.

Yet, for most known products from Table 1, the yield obtained using our simple and robust method is at least as high as yields obtained previously.^18,24,25^ Additionally, the reaction time is significantly shorter, and the workup typically consisted only of a simple filtering step through a short silica plug. To confirm the accuracy of the NMR yield, we repeated several reactions on a 0.51 mmol **1** scale, and isolated the products. The thus obtained isolated yields—given in brackets in Table 1—were all within 5% of the NMR yields reported above.

The base used in this reaction to generate the reactive phenolate is somewhat dependent on the substrate. Often NaH is favored for two reasons: (1) the base is removed as hydrogen gas during the deprotonation step; (2) the sodium cation precipitates with the nitrophenolate, allowing this side product of the reaction to be filtered off. However, for some phenols containing additional base-sensitive groups (e.g., **2n**, **2y**, **2z**), treatment with NaH caused degradation. For phenols **2y** and **2z**, the milder organic bases BTMG or DBU were shown to be a better choice to provide the desired product, though for compound **3z**, purification to a pure product was not possible without causing (partial) degradation. Phenol **2n** still only showed degradation products regardless of base, but product **3y** could be isolated and purified following a reaction of **1** and **2y** with either BTMG or DBU. However, with these bases, a column or extraction was needed to purify the final product, instead of the simple filtering through silica required for NaH reactions. Several other common bases were also tested for their ability to perform the exchange reaction, but these typically yielded lower yields (Supporting Info, Table S1). Apart from this, there were also some phenols–those containing electron-withdrawing groups on the *ortho* position (**2k-***o*, **2x**, **2ad**)—that did not react at all under the conditions specified above. This is attributed to the combination of reaction-diminishing steric and electronic effects, as phenols containing other bulky, but electron-donating, groups at the *ortho* position (**2o-***o*, **2ae**) did give 100% yield, albeit at a reduced reaction rate. Here especially **2ae** is remarkable, as the corresponding reaction product on sulfonimidoyl fluorides did not give significant amounts of substitution products.^16^

After these first results, the scope of the exchange reaction was investigated by testing the ability of the nitrophenolate on **1** to exchange with a range of alkyl alcohols **4a**–**f** and naturally occurring phenols **6a**–**k** (Table 2). Similar to the phenols in Table 1, a quantitative yield was obtained for most of the alkyl alcohols; only for the sterically hindered *tert*-butyl alcohol **4c**, no product was formed. The efficacy is perhaps most clearly shown for the reaction with glycerol (**4f**): even with only a minor excess of **1** (3.1 equiv), only one product, displaying triple addition of **1** to glycerol, is observed upon workup. This marks a significant step forward, as under classical SuFEx conditions saturated alcohols are typically under-reactive, and can only be induced to react upon addition of both HMDS and BTMG, as shown by Moses’ group.^18^ Here, no additional agents are needed to obtain quantitative sulfonate product formation with saturated alcohols.

**Table 2:**
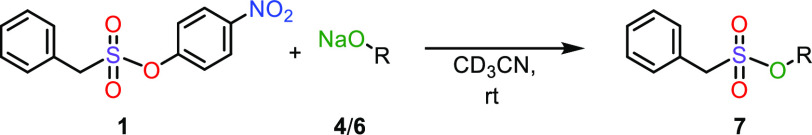

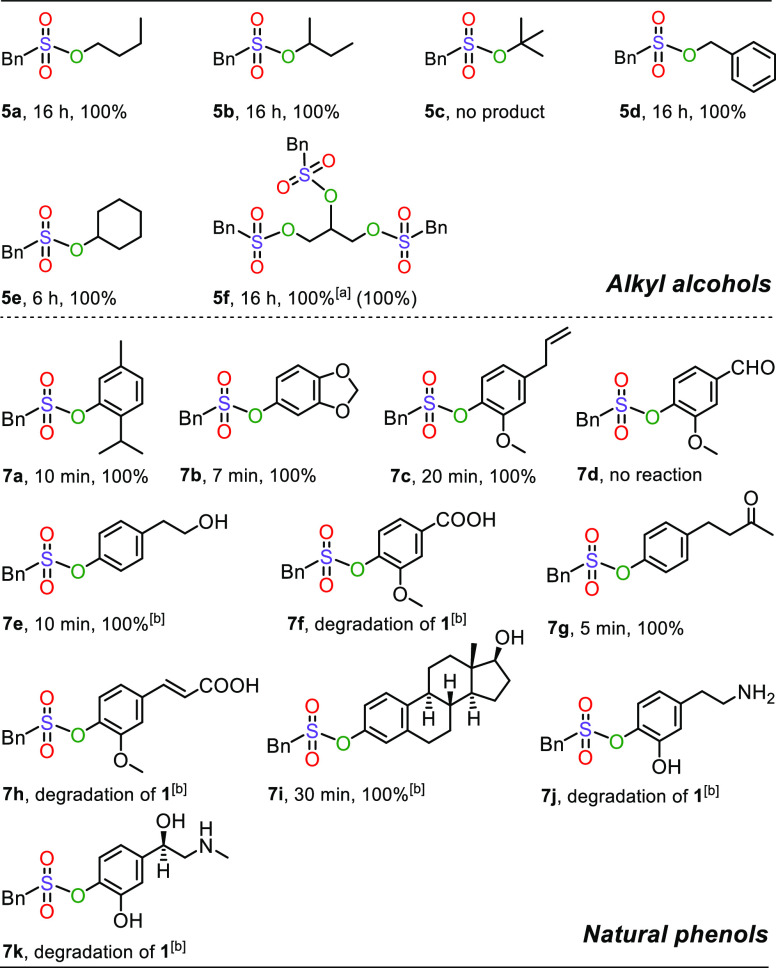
Extension of Scope of the S(VI) Exchange Reaction from **1**: Saturated Alcohols and Natural Phenols

a 3 equiv. of NaH and 3.1 equiv. of **1** used.

b 2 equiv. of NaH used.

c Yields were determined by ^1^H NMR measurements. Reaction conditions: 0.10 mmol of **1** and 1.1 equiv. of **4/6** in 0.6 mL of CD_3_CN.

While the yields for saturated alcohols are as high as observed for phenols, there is a significant increase in the reaction time needed to reach full conversion: 6–16 h compared to the typically <5 min for the phenols. This clearly shows the difference in reactivity between phenols and alkyl alcohols under these conditions. However, when 1 equiv of 15-crown-5 was added to capture the Na^+^ ion, the reaction time for alkyl alcohols (tested for **4a** and **4e**) is decreased to less than 15 min, close to the reaction times observed for most phenols. Such addition also helped to speed up the reaction for some of the slower-reacting phenols, such as **2w** or **2af**. As the addition of 15-crown-5 ether prevents the formation, and therefore precipitation, of the sodium 4-nitrophenolate salt, we conclude that, while advantageous from the point of easy purification, precipitation of the sodium nitrophenolate as a means of removing one of the products of the reaction is not an essential component of the driving force of the reaction.

When looking at the results for the naturally occurring phenols, a similar trend is observed as for phenols **2a**–**2af**. Again, phenols containing an additional base-sensitive acid group (**6f**, **6h**) show degradation upon interaction with NaH, while most other phenols again give quantitative yields. Also phenols with two hydroxyl groups (**6j** and **6k**) situated *ortho* to each other show degradation, though in this case this is likely due to a strong electronic repulsion between the additional (deprotonated) hydroxyl group on the attacking phenolate and the oxygen atoms on the sulfonyl group during the nucleophilic attack of the phenolate, as these atoms will be in close proximity to each other in the transition state. As a result of this strong electronic repulsion, the attacking phenolate cannot approach close enough to form the desired product, and degradation of **1** occurs instead. Finally, the phenol containing an aldehyde group (**6d**) gave no reaction, while this functionality led to degradation before (**2n-***p*). Apparently, the additional methoxy group on the ortho position prevents degradation, yet the presence of the aldehyde moiety still prevents exchange with the nitrophenolate on **1**.

To investigate whether not just benzylic sulfonates but also aryl sulfonates would undergo this transformation, we repeated the exchange reaction for phenols **2a**, **2e**, **2k**–**p**, and alcohol **4b** using 4-nitrophenyl 4-methylbenzene sulfonate. The selected phenols and alcohol represent the full range of electron-donating to electron-withdrawing aryl substituents, as well as an aliphatic alcohol. All reactions gave good yields, though the reaction times were increased compared to the reaction with **1**: 40 min for **2a** (100% yield), 10 min for **2e** (100% yield), 85% conversion after 5 days for **2k**–**p**, and 95% conversion after 5 days for **4b**. This indicates that, while aryl sulfonates can be used, the reactivity is greatly decreased compared to benzylic sulfonates.

To further explore the relevance of this reaction, we investigated its potential for dynamic covalent chemistry, as such a feature would significantly widen the applicability of this exchange chemistry in the field of materials and polymer science. In principle, a leaving group phenolate can–if it is sufficiently nucleophilic–reattach to the sulfonate to regain the starting material. This was not observed for any of the phenols or alcohols tested here, as the nitro group on the nitrophenol has a much stronger electron-withdrawing effect than any other substituent or alcohol tested. However, for more nucleophilic phenolates such exchange might be feasible. So, to test this, product **3f** (1 equiv) was exposed to 1 equiv of NaH and 1 equiv. of 4-bromophenol (**2h-***p*). At the same time, product **3h-***p* (1 equiv) was exposed to 1 equiv of NaH and 1 equiv of 4-chlorophenol (**2f**). As 4-bromophenol and 4-chlorophenol have a similar electron-withdrawing effect (Hammett parameter of 0.23 for both substituents),^26^ an equilibrium between products **3f** and **3h-***p* should be reached. Indeed, after 6 days of reaction time, a ratio of 1:1 **3f**:**3h-***p* was found in both reaction mixtures. This proves that the phenol sulfonates undergo a dynamic covalent reaction, albeit slowly, at room temperature. In addition, the easy control of the driving force of the SuPhenEx reaction via the para-substituents on the nucleophile and/or leaving group allows fine-tuning of e.g. the degree and rate of replacement. In order to increase the atom efficiency of the reaction, we have studied the recyclability of the excess 4-nitrophenol used in the production of the starting material. We could recover >85% of the 4-nitrophenol, which was used to make fresh starting material **1** (see the Supporting Information).

In addition, the exchange reaction was tested for its ability to degrade a polysulfonate polymer. Such polymers have become easily available via SuFEx click chemistry, both in a step-growth^11^ as well as in a chain-growth fashion.^10^ When the model polymer **8** (*M*_*n*_ = 27 kDa) was treated with 10 equiv of sodium phenolate in THF at 80 °C, the molecular weight of the degraded products was reduced to ≤0.7 kDa within 24 h (as measured by gel permeation chromatography), indicating the complete degradation of polymer **8** (Figure 2). Furthermore, LC-MS analysis of the degraded polymer showed fragments **9**, **10** and **11** as the major degradation products (Figure 3). Additionally, trace amounts of product **12** as observed in LC/MS confirmed the formation of also this degradation product. Based on this, we propose a possible degradation pathway of the polymer (Figure 3, see Supporting Info for more details). This result shows the potential of the reversible SuPhenEx reaction in depolymerization.

**Figure 2:**
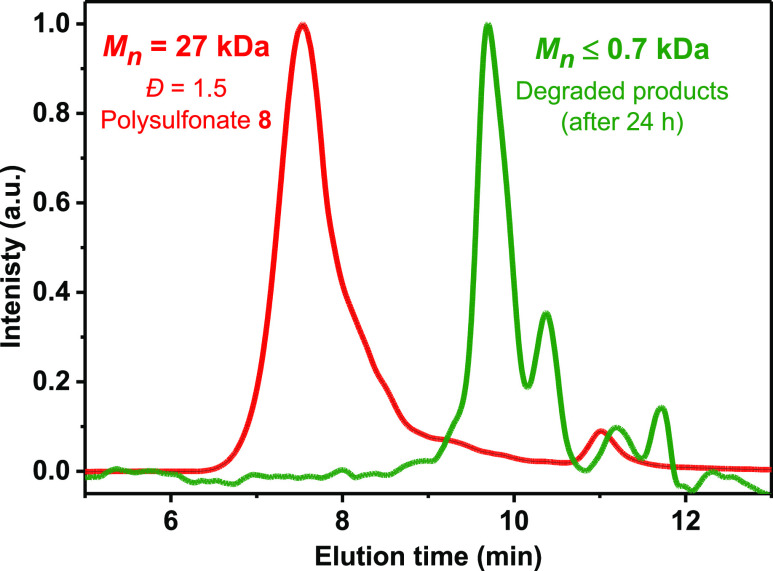
GPC traces of polymer and degraded products.

**Figure 3:**
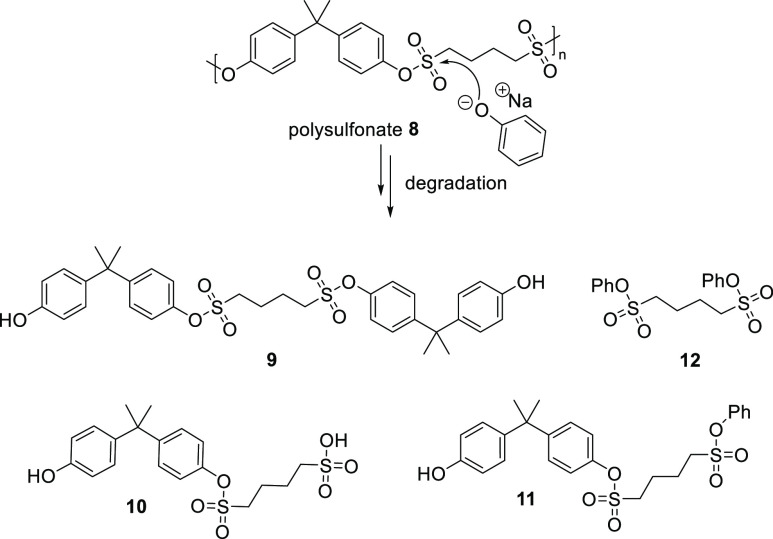
Degradation of polysulfonate **8**.

In conclusion, we provide a simple, fast, and highly efficient click reaction to create a wide range of S(VI) alcohol exchange compounds starting from a single easy-to-produce and stable starting material. The reaction is fluorine-free, fast and typically quantitative, the workup facile, the driving force tunable, and the reaction shows a very wide functional group tolerance, which we thus expect to have significant use in a wide range of the molecular sciences.

## Notes

All underlying data is available in the article itself and its Supporting Information.

## Supporting Information Available

Experimental details, supporting figures/tables, and characterization of novel compounds (PDF)
