# Supplementary material for: Sulfur-Phenolate Exchange As a Fluorine-Free Approach to S(VI) Exchange Chemistry on Sulfonyl Moieties
Source: Org Lett. 2022 Nov 16;24(47):8621–6. doi: 10.1021/acs.orglett.2c03421 (PMC9724081; doi:10.1021/acs.orglett.2c03421)
Supplement: Supplementary file 1 — ol2c03421_si_001.pdf [file ol2c03421_si_001.pdf]

Supporting information

Sulfur-Phenolate Exchange as a fluorine-free approach to S(VI) exchange chemistry on sulfonyl moieties

Alyssa F.J. van den Boom,[a] Muthusamy Subramaniam,[a, b] and Han Zuilhof*[a,b,c]

[a] Laboratory of Organic Chemistry, Wageningen University, Stippeneng 4, 6708WE Wageningen, The Netherlands.

[b] School of Pharmaceutical Science and Technology, Tianjin University, 92 Weijin Road, Tianjin 300072, China.

[c] Department of Chemical and Materials Engineering, Faculty of Engineering, King Abdulaziz University Jeddah 21589, Saudi Arabia.

Contents

General information **2**
Chemicals 2
Analysis 2
Synthetic procedures **4**
4-Nitrophenyl phenylmethanesulfonate 1 4
General procedure for the synthesis of products (for NMR screening) 4
Products for isolated yields and full characterization 4
Reaction with 2-tert-butyl-1,1,3,3-tetramethylguanidine (BTMG) or DBU 4
Base screening 5
Hydrolytic stability 5
Recycling experiment 5
Polymer synthesis 5
Degradation of polysulfonate 8 6
Supporting tables & figures **7**
Hydrolytic stability 7
Base screening 9
Characterization of compounds **10**
NMR spectra of novel compounds **22**
GPC distribution plots **60**
LC Mass Spectra of the degraded polysulfonate 8 .... **61**
References **62**

General information

Chemicals

Sodium hydride (60% dispersion in mineral oil), 4-nitrophenol, phenylmethylsulfonyl fluoride, phenol, 4-methoxyphenol, 4-cyanophenol, 4-chlorophenol, 4-aminophenol, p-cresol, m-cresol, 4-hydroxybenzamide, 3,4,5-trifluorophenol, 3,5-bis(trifluoromethyl)phenol, 4-bromophenol, 2-bromophenol, 3-bromophenol, 4-fluorophenol, 2-fluorophenol, 4-methyl-2-nitrophenol, acetaminophen, 4-hydroxybenzyl alcohol, resorcinol, vanillic acid, bisphenol A, 2-cyanophenol, sesamol, trans-ferulic acid, salicylaldehyde, vanillin, β-estradiol, dopamine hydrochloride, and (−)-epinephrine were bought from Merck Life Science N.V.; acetonitrile-d3, 2,6-dichlorophenol, 4-hydroxybenzoic acid, 3-fluorophenol, and 4-hydroxyphenylboronic acid were bought from Fisher Scientific B.V.; o-cresol, 2,4,6-trimethylphenol, 3-hydroxypyridine, 2-tert-butylphenol, 4-hydroxybenzaldehyde, eugenol, 4-(4-hydroxyphenyl)-2-butanone, 2-(4-hydroxyphenyl)ethanol, and thymol were bought from TCI EUROPE N.V.; 4-(trifluoromethoxy)phenol and 4-((4-isopropoxyphenyl)sulfonyl) phenol were bought from Fluorochem Ltd; 4-iodophenol was bought from VWR International B.V. All chemicals were used as received.

Analysis

NMR measurements were conducted on a 400 MHz Bruker Avance III at 298K, and the resulting data were analyzed using MestReNova software, version 14.1.0-24037. Spectra were calibrated relative to signals corresponding to the non-deuterated solvents (CH_3CN solvent peak) – at 1.94 ppm for ^{1}H spectra, and 1.32 ppm for ^{13}C spectra. For spectra measured in $CDCl_3$ calibration was performed with the $CHCl_3$ peaks at 7.26 ppm (^{1}H spectra) and 77.16 ppm (^{13}C spectra). ^{19}F spectra were not calibrated. Abbreviations used in the description of NMR data are as follows: chemical shift (δ = ppm), multiplicity (s = singlet, d = doublet, t = triplet, q = quartet, p= pentet, sextet = sext, h = heptet, dt = doublet of triplets, m = multiplet, br = broadened), coupling constant (*J*, Hz).

High-resolution mass spectra (HRMS) were recorded on a Thermo Scientific Exactive 1.1 with an orbitrap mass analyzer, using a DART gun from Ion Sense. The temperature of the DART gun was set to 400 °C, with a helium gas flow. Data were analyzed using Thermo Xcalibur software, version 2.2 SP1.48.

IR spectra were recorded on a Bruker Tensor 27 spectrometer equipped with a diamond ATR accessory (64 scans; 4 cm^{-1} resolution; range 4000-350 cm^{-1}). Strong or indicative peaks in the region 4000-1000 cm^{-1} are mentioned for each novel compound.

TLC analysis was performed on pre-coated, alumina-backed silica gel plates. TLC plates were analyzed by UV fluorescence (254 nm) or $KMnO_4$ stain followed by heating.

Column chromatography for isolated yields and full characterization was performed using silica (40-63 µm, 230-400 mesh) and 20-50% ethyl acetate in hexane, depending on the polarity of the product. To perform flash chromatography, a connector connected to a compressed-air flow was installed on the column.

Gel Permeation Chromatography (GPC). The polymer molecular weight (M_n^{ps}) and polydispersity (Đ) relative to a set of polystyrenes were measured on an Agilent Technologies 1200 Gel Permeation Chromatography (GPC) system. It was equipped with a diode-array detector (DAD, G1315D, data obtained using a 250 nm, 260 nm, and 290 nm UV-vis detector), a refractive index detector (RID, G1362A), autosampler (ALS, G1329A), bin pump (G1312A), solvent degasser (G1322A). GPC equipped with VARIAN GPC/SEC column from PLgel 5 µm MIXED-D (300 × 7.5 mm) part number: PL1110-6504. The system was calibrated with EasyVial PS-M and EasyVial PS-L polystyrene standards (Agilent Technologies), GPC polystyrene standards with a combined range from Mp 400 to 2,000,000 Da (Fluka). HPLC-grade *N*,*N*-dimethylformamide (DMF, Biosolve®, unstabilized, HPLC grade) with 0.01% LiBr (ReagentPlus®, ≥99% from Sigma-Aldrich) was used as mobile phase for determining the molecular weight polymers. The elution rate was 1.0 mL/min (all GPC performed at room temperature).

Liquid chromatography–mass spectrometry LC-MS analysis was performed using Agilent 1290 Infinity UHPLC system and Thermo Scientific Q Exactive Focus Mass Spectrometer in electrospray ionization (ESI^+) mode. LC-MS grade acetonitrile (HiPerSolv CHROMANORM®) and millipore water with 0.1 % formic acid were used as mobile phase in gradient flow. Eclipse XDB C-18 HPLC column was used with flow rate 0.4 mL/min, oven temperature 25 °C, detector wavelength 240 nm and 280 nm.

Synthetic procedures

Unless mentioned otherwise, all reactions were performed at room temperature under ambient atmosphere.

4-Nitrophenyl phenylmethanesulfonate 1

NaH (60% in oil, 293 mg, 7.34 mmol, 1.4 equiv) was added to 4-nitrophenol (948 mg, 6.82 mmol, 1.3 equiv) in 80 ml of dry tetrahydrofuran, and the solution was stirred for 5 min. Next, phenylmethanesulfonyl chloride (1.0 g, 5.25 mmol, 1.0 equiv) was added, and stirring was continued for 24 h. Then, the reaction mixture was diluted with 150 ml of DCM and transferred to a separatory funnel, where it was washed with water (3 × 150 ml). The organic phase was isolated, dried over sodium sulphate, and the solvent was evaporated. Next, the product was further purified by column chromatography (n-hex/EA) (using a Biotage® system and SiliCycle® precast silica columns (200−300 mesh or 300−400 mesh)) to yield compound **1** (1.37 g, 89%) as white crystals.

General procedure for the synthesis of products (for NMR screening)

In a typical exchange reaction, 5 mg of NaH (0.124 mmol, 1.2 equiv) and 0.113 mmol (1.1 equiv) of phenol/alcohol were dissolved in 0.9 mL of CD_3CN, and the reaction was stirred for ~5 min to generate the phenolate. After this time, 30 mg (0.103 mmol, 1.0 equiv) of **1** was added, and stirring was continued. TLC (25% ethyl acetate in hexane) was used to monitor the progress of the reaction by the disappearance of the spot belonging to 4-nitrophenyl phenylmethanesulfonate. When this spot had fully disappeared, the reaction mixture was filtered into an NMR tube through a short silica plug, to remove the sodium nitrophenolate. The filtered reaction mixture was then directly analyzed using ^1H NMR. No internal standard was needed in this case, as the sodium nitrophenolate was filtered off, and no other side products – except in the case of degradation – are formed during the reaction. As a result, the reaction mixture only contains the product and any remaining starting material, both of which provide well-separated signals in ^1H NMR. The yield could therefore be determined by the disappearance of the signals belonging to 4-nitrophenol, with a simultaneous appearance of product signals. A reference spectrum of the reactant phenol was recorded in all cases, and successful binding was further confirmed by a change in chemical shift for the signals belonging to the added phenol upon attachment to the S(VI) hub. When the reaction was filtered prematurely – or when conversion was <100% – signals from an attached nitrophenol group were still visible (though with a lower integral value), and the product signals of the partially attached phenol had lower integrals than expected based on full conversion. When degradation took place, no product signals were observed, and signals from the starting material had shifted or disappeared.

Products for isolated yields and full characterization

Compounds for which the isolated yield was determined, and novel compounds that had to be characterized more extensively, were synthesized in the same way as described above for the NMR products, using non-deuterated acetonitrile as the solvent, on a scale of 150 mg (0.51 mmol) of **1**. After the reaction was completed, the solvent was evaporated, and the products were purified using flash column chromatography (silica gel) with 25%-40% ethyl acetate in hexane, depending on the polarity of the product. For highly polar products (e.g. **7i**), 5% methanol in DCM was used as the mobile phase.

Reaction with 2-tert-butyl-1,1,3,3-tetramethylguanidine (BTMG) or DBU

25 µl of BTMG or 17.5 µl of DBU (0.117 mmol, 1.1 equiv) and 0.113 mmol (1.1 equiv) of phenol were dissolved in 0.9 mL of CD_3CN, and the reaction was stirred for ~10 min to generate the phenolate. After this time, 30 mg (0.103 mmol, 1 equiv) of **1** was added, and stirring was continued. TLC (25% ethyl acetate in hexane) was used to monitor the progress of the reaction by the disappearance of the spot belonging to 4-nitrophenyl phenylmethanesulfonate. When this spot had fully disappeared, the reaction mixture was either transferred into an NMR tube for direct analysis using ^1H NMR, or purified for characterization.

Base screening

For the base screening, the same protocol was used as described above for BTMG, with a few adjustments: 0.117 mmol of the base mentioned in the left column of Table S1 was used instead of BTMG; compound **1** was added after 20 min instead of 10; and the exchange reaction after addition of **1** was run for 15 min, before the reaction mixture was filtered into an NMR tube for analysis.

Reaction with 4-cyanophenyl- or 4-chlorophenyl phenylmethanesulfonate

Reactions using either 4-cyanophenyl phenylmethanesulfonate or 4-chlorophenyl phenylmethanesulfonate as starting material (instead of **1**) were performed in the same way as described in the general procedure, though CH_3CN was used instead of CD_3CN. After synthesis had been completed, the solvent was evaporated on a rotavapor, and the products were purified using flash column chromatography (silica gel, 30% ethyl acetate in hexane).

Reaction with 4-nitrophenyl 4-methylbenzenesulfonate

Reactions performed with 4-nitrophenyl 4-methylbenzenesulfonate instead of **1** as a starting material were performed as described in the general procedure, on a scale of 90 mg (0.307 mmol) 4-nitrophenyl 4-methylbenzenesulfonate, and using CH_3CN as the solvent. When full conversion was reached, the solvent was evaporated on a rotavapor, and the crude product was purified using flash column chromatography (silica gel, 30% ethyl acetate in hexane).

Hydrolytic stability

To test the hydrolytic stability of compound **1**, 50 mg of **1** was added to 5 ml of a 20% solution of DMSO in water. As compound **1** does not dissolve in this solution, the resulting suspension was stirred vigorously on a stir plate. Degradation of **1** was followed by absorption measurements on a spectrophotometer. The increase in absorption arising from the nitrophenol degradation product was taken as a measure for the amount of degradation.

Recycling experiment

The starting material **1** (1.0 g, 3.41 mmol, 1 equiv) was added to a stirred solution of sodium phenolate (0.395 g, 3.41 mmol, 1 equiv) in dry CH_3CN (15 mL) and allowed to stir for an hour. The reaction mixture was quenched with water (100 mL), diluted with CH_2Cl_2 (100 mL) and adjusted to pH = 10 with 1 M NaOH (aq). Then the aqueous layer was separated, diluted with CH_2Cl_2 (100 mL), adjusted to pH = 6 with 1 M NaOH (aq) and two layers were separated. The aqueous layer was extracted with CH_2Cl_2 (2 × 30 mL) and the combined organic layers were dried over Na_2SO_4 and concentrated under vacuum to give 4-nitrophenol (0.408 g, 86%). This 4-nitrophenol was reacted to NaH (60% in oil, 164 mg, 4.10 mmol, 1.4 equiv) followed by phenylmethanesulfonyl chloride (559 mg, 2.93 mmol, 1.0 equiv) in dry THF (10 mL) to prepare the fresh starting material **1** (705 mg, 82%).

Polymer synthesis

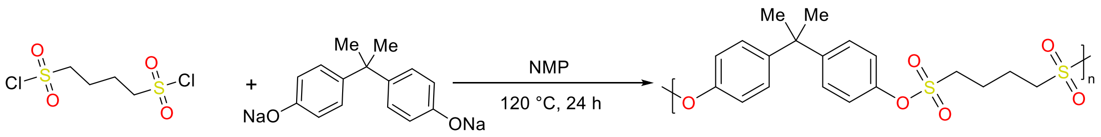

Inside an argon-filled glovebox (MBRAUN's MB 20 G-LMF gas purifier with H_2O and O_2 values of <0.1 ppm), butane-1,4-disulfonyl dichloride (0.5 g, 1.96 mmol, 1 equiv) and disodium bis-phenolate (0.53 g, 1.96 mmol, 1 equiv) were added in a 5 ml screw-capped glass vial equipped with a magnetic stir bar. 1.25 mL of anhydrous *N*-methyl-2-pyrrolidone (Thermo Scientific 99.9+% Extra Dry over Molecular Sieve, AcroSeal®) was added, and the vial was taken out of the glovebox. The reaction mixture was heated to 120 °C using an oil bath, and stirred for 24 h, during which course the reaction mixture turned viscous. Next, the reaction mixture was cooled, and 4 mL DMF was added to dissolve the crude polymer. The resulting solution was poured slowly into 30 mL of MeOH under constant stirring. After a precipitate formed, the stirring was continued for another 10 min. The formed precipitate was then allowed to sediment for 10

min, after which the methanol layer was removed and a minimal amount of DMF was added to re-dissolve the precipitate. This precipitate, sediment, and re-dissolve cycle was repeated, for a total of 4 cycles. Afterwards, the polymer was purified by dialysis (Sigma-Aldrich, Pur-A-Lyzer™ Mega 6000) in DMSO, and subsequently used for degradation experiments. The molecular weight M_n of polymer **8** was found to be 27 kDa (*Đ* = 1.54) with GPC measurement.

Degradation of polysulfonate 8

NaH (9.8 mg, 0.24 mmol, 10 equiv) was added to phenol (23 mg, 0.24 mmol, 10 equiv) in 1 ml of tetrahydrofuran, and the solution was stirred for 5 min. Next, polysulfonate **8** (10 mg, 1.0 equiv) was added, and the stirring was continued for 24 h at 80 °C in an oil bath. Then the mixture was diluted with DCM (3 mL) and water (3 mL), adjusted to pH ~ 5 with 0.05 M HCl. Then, the organic layer was washed with water (2 × 3 mL), dried over Na_2SO_4, and used for GPC and LC-MS analysis.

Plausible mechanism for the degradation of polymer 8:

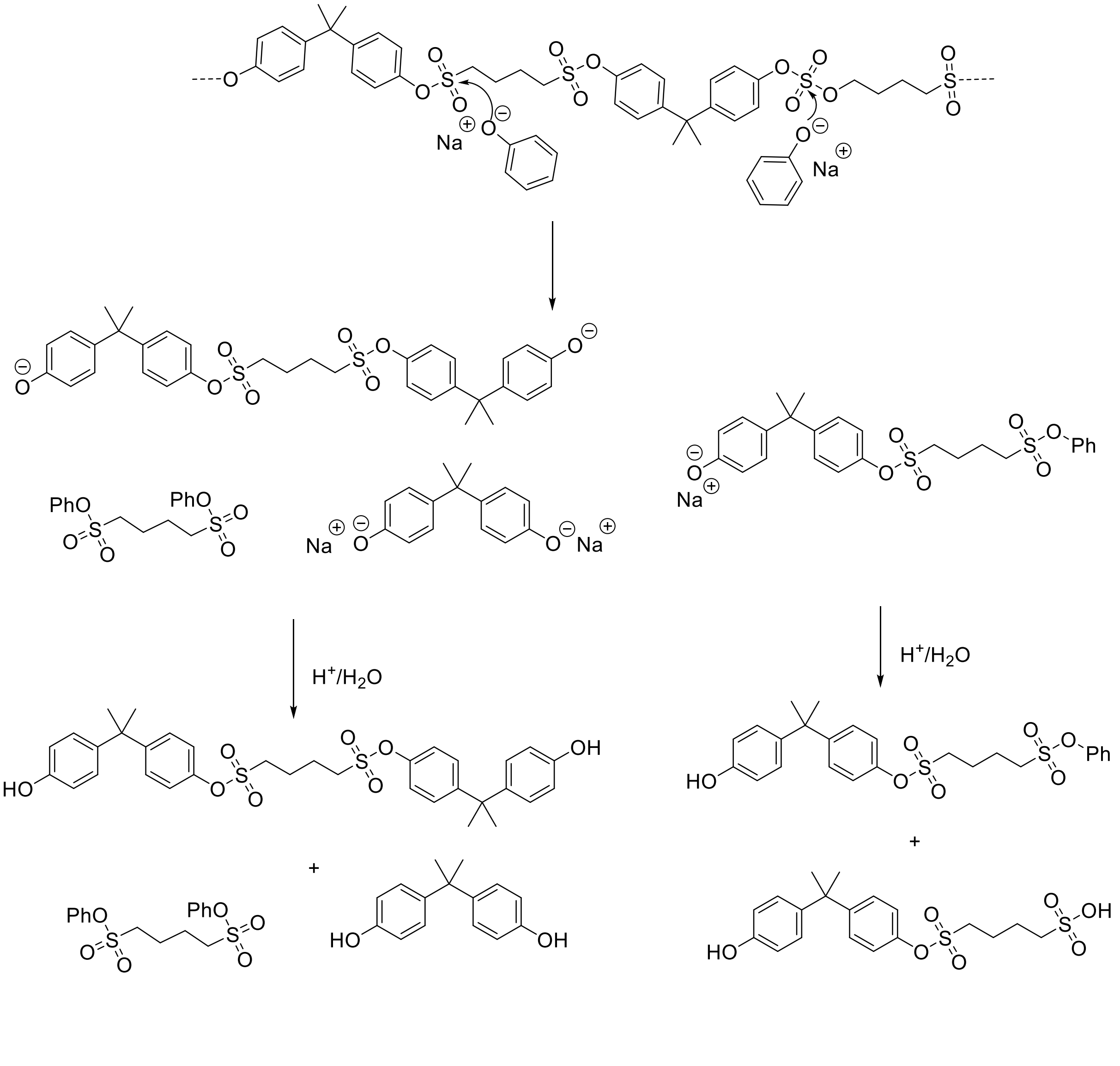

Supporting tables & figures

Hydrolytic stability

To measure the degradation of **1**, the optical absorption at 418 nm was followed, as at this wavelength **1** does not have any significant absorption (Figure S1). The degree of degradation (in %) was calculated using a calibration curve of 4-nitrophenol in 20% DMSO in water.

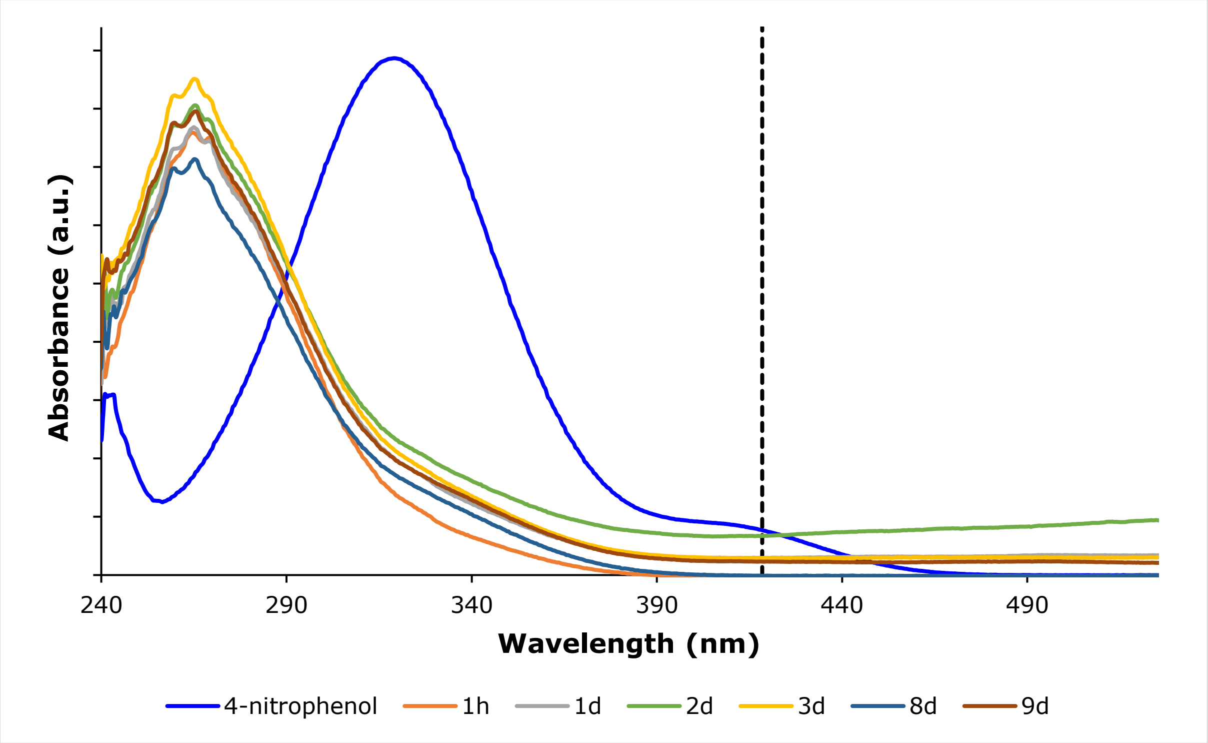

Figure S1: absorption spectra of **1** in 20% DMSO in water after different exposure times. The spectrum of 4-nitrophenol is shown in blue. The vertical dashed line indicates the wavelength at which the amount of degradation was determined (418 nm).

The results after 9 days of exposure to 20% DMSO in water show only minimal degradation (<0.09%, Figure S2). However, the actual degradation is even lower, as the absorption across the whole spectrum was increased for several time points, due to the turbidity of the sample. This can also be clearly seen in Figure S1 above for the spectra of *e.g.* 2d, 3d, and 9d.

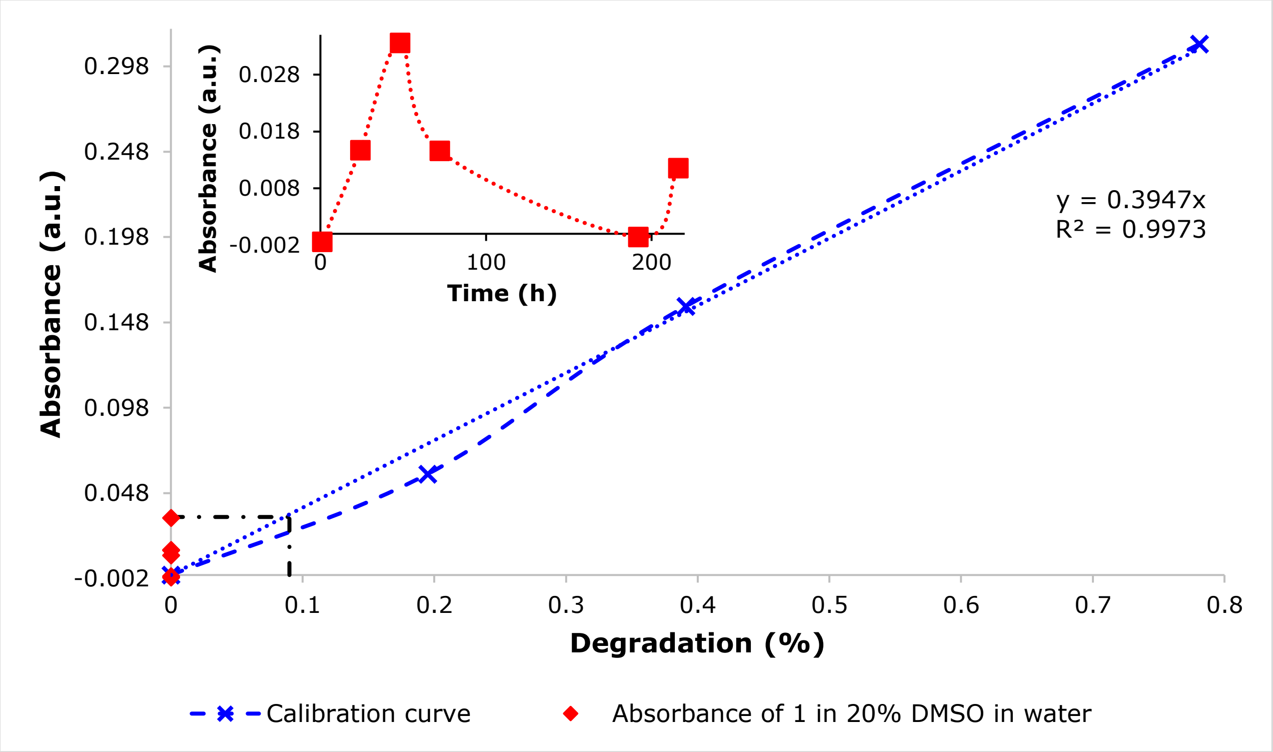

Figure S2: Optical absorption values at 418 nm for **1** in 20% DMSO in water, compared to the absorption of different concentrations of 4-nitrophenol in the same solvent mixture (calibration curve). The insert shows the absorption at 418 nm over time, for the solution of **1** in 20% DMSO in water.

An NMR spectrum taken after 9 days of the exposed sample confirms no degradation has taken place.

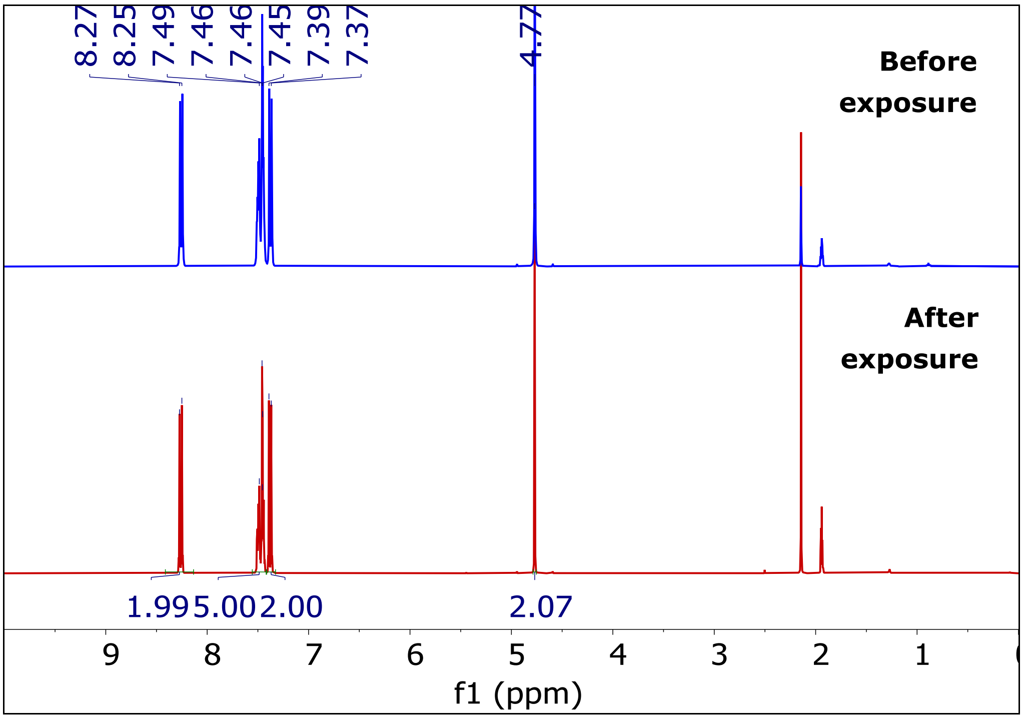

Figure S3: ^{1}H NMR spectrum (400 MHz, CD_3CN) of **1** before (blue upper spectrum) and after (red bottom spectrum) 9 days exposure to a 20% solution of DMSO in water.

Base screening

In the table below, conversion refers to the percentage of **1** that has reacted, while yield refers to the percentage of the desired product found in the reaction mixture. In cases where the conversion is higher than the yield, side reactions or degradation of **1** occurred.

Table S1: Overview of common bases tested for the exchange reaction of **1** with phenol.

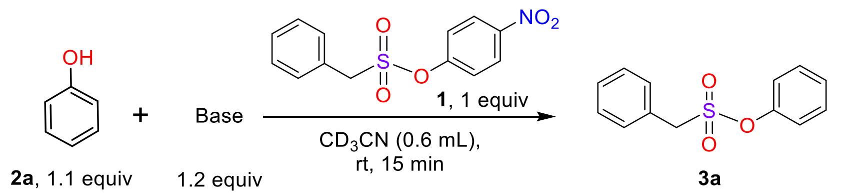

Base	Yield (%)	Conversion (%)
Sodium hydride (NaH)	100	100
Potassium hydride (KH)	Degraded	100
Lithium hydride (LiH)	50	50
Potassium hydroxide (KOH)	55	55
Cesium hydroxide monohydrate ($CsOH•H_2O$)	84	84
Potassium tert-butoxide (KOtBu)	25	100
Lithium diisopropylamide (LDA)	Degraded	100
Triethylamine $(Et)_3N$	53	53
N,N-Diisopropylethylamine (DIPEA)	46	46
2-tert-Butyl-1,1,3,3-tetramethylguanidine (BTMG)	100	100
1,4-Diazabicyclo[2.2.2]octane (DABCO)	72	72
1,8-Diazabicyclo[5.4.0]undec-7-ene (DBU)	100	100
None	0	0

Characterization of compounds

3a. Phenyl benzylsulfonate

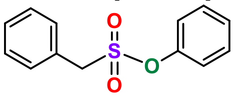

Prepared according to the general procedure described above using phenol as the phenol. White solid. Yield: 121 mg (95%)

^{1}H NMR (CD_3CN, 400 MHz): δ 7.53 – 7.41 (m, 7H), 7.39 – 7.33 (m, 1H), 7.25 – 7.18 (m, 2H), 4.68 (s, 2H).

This product has been reported in >20 publications, with the first mention in Zaborsky & Kaiser (1966).[1]

3b-*o*. 2-Methylphenyl benzylsulfonate

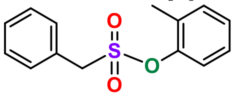

Prepared according to the general procedure described above using *o*-cresol as the phenol. NMR yield: 100%

HRMS: *m/z* $C_{16}H_{17}NNaO_3S^+$ ($[M+ACN+Na]^+$): calculated 326.0821, found 326.0822

^{1}H NMR (CD_3CN, 400 MHz): δ 7.55 – 7.48 (m, 2H), 7.48 – 7.43 (m, 3H), 7.33 – 7.27 (m, 1H), 7.26 – 7.20 (m, 2H), 7.16 – 7.10 (m, 1H), 4.73 (s, 2H), 2.22 (s, 3H). **^{13}C NMR (CD_3CN, 101 MHz):** δ 148.9, 132.8, 132.7, 132.1, 130.1, 129.8, 129.1, 128.3, 128.2, 123.1, 57.7, 16.7.

$\bar{\nu}_{max}$: 3062, 3032, 2935, 1593, 1489, 1456, 1363, 1180, 1146, 1099 cm^{-1}.

3b-*m*. 3-Methylphenyl benzylsulfonate

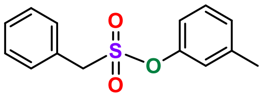

Prepared according to the general procedure described above using *m*-cresol as the phenol. White solid. NMR yield: 100%

HRMS: *m/z* $C_{16}H_{17}NNaO_3S^+$ ($[M+ACN+Na]^+$): calculated 326.0821, found 326.0824.

^{1}H NMR (CD_3CN, 400 MHz): δ 7.52 – 7.42 (m, 5H), 7.35 – 7.27 (m, 1H), 7.18 (d, *J* = 7.6 Hz, 1H), 7.04 – 6.97 (m, 2H), 4.67 (s, 2H), 2.35 (s, 3H). **^{13}C NMR (CD_3CN, 101 MHz):** δ 150.39, 141.63, 132.02, 130.70, 130.10, 129.84, 129.03, 128.95, 123.51, 120.01, 57.19, 21.25

$\bar{\nu}_{max}$: 3066, 3035, 2927, 1610, 1583, 1487, 1456, 1367, 1174, 1122 cm^{-1}.

3b-*p*. 4-Methylphenyl benzylsulfonate

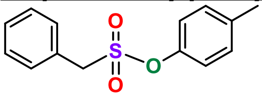

Prepared according to the general procedure described above using *p*-cresol as the phenol. NMR yield: 100%

^{1}H NMR (CD_3CN, 400 MHz): δ 7.53 – 7.38 (m, 5H), 7.24 (d, *J* = 8.3 Hz, 2H), 7.09 (d, *J* = 8.5 Hz, 2H), 4.65 (s, 2H), 2.34 (s, 3H).

Product was previously reported in Alonso *et al.* (2005) & Moghaddam *et al.* (2004).[2,3]

3c. 4-Aminophenyl benzylsulfonate

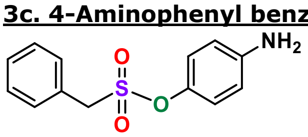

Prepared according to the general procedure described above using 4-aminophenol as the phenol. brown solid. The secondary product that was formed during this reaction – where phenol **2c** had attached to **1** *via* the -NH_2 moiety – was separated from the desired product using column chromatography (silica gel,

gradient of 53-65% ethyl acetate in hexane), and the product distribution that was found using NMR (75% **3c**, 25% secondary product) was confirmed by isolating and weighing both products.

HRMS: *m/z* $C_{13}H_{14}NO_3S^+$ ($[M+H]^+$): calculated 264.0689, found 264.0690.

^{1}H NMR (CD_3CN with 20% MeOD, 400 MHz): δ 7.50 – 7.40 (m, 5H), 6.93 (d, *J* = 8.9 Hz, 2H), 6.64 (d, *J* = 8.9 Hz, 2H), 4.58 (s, 2H), 4.27 (s, 2H). **^{13}C NMR (CD_3CN with 20% MeOD, 101 MHz):** δ 148.3, 141.3, 132.0, 130.0, 129.8, 129.2, 123.8, 115.8, 56.5.

$\tilde{\nu}_{max}$: 3475, 3385, 3063, 3034, 2924, 2854, 1622, 1502, 1362, 1169, 1144 cm^{-1}.

<u>3d. 2,4,6-Trimethylphenyl benzylsulfonate</u>

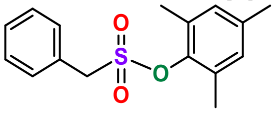

Prepared according to the general procedure described above using 2,4,6-trimethylphenol as the phenol. White solid. Yield: 143 mg (96%)

^{1}H NMR (CD_3CN, 400 MHz): δ 7.54 (dd, *J* = 6.6, 3.2 Hz, 2H), 7.48 – 7.41 (m, 3H), 6.92 (s, 2H), 4.77 (s, 2H), 2.25 (s, 3H), 2.22 (s, 6H).

Product was previously reported in Alonso *et al.* (2005) & Truce *et al.* (1970).[2,4]

<u>3e. 4-Methoxyphenyl benzylsulfonate</u>

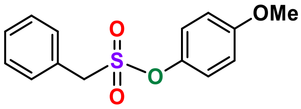

Prepared according to the general procedure described above using 4-methoxyphenol as the phenol. White solid. Yield: 142 mg (100%)

^{1}H NMR (CD_3CN, 400 MHz): δ 7.51 – 7.40 (m, 5H), 7.13 (d, *J* = 9.2 Hz, 2H), 6.95 (d, *J* = 9.2 Hz, 2H), 4.64 (s, 2H), 3.79 (s, 3H).

Product was previously reported in Moghaddam *et al.* (2004) & Betts *et al.* (2006).[3,5]

<u>3f. 4-Chlorophenyl benzylsulfonate</u>

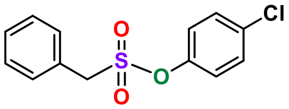

Prepared according to the general procedure described above using 4-chlorophenol as the phenol. White solid. Yield: 143 mg (99%)

^{1}H NMR (CD_3CN, 400 MHz): δ 7.51 – 7.40 (m, 7H), 7.20 (dt, *J* = 8.9, 3.2 Hz, 2H), 4.69 (s, 2H).

Product was previously reported in Alonso *et al.* (2005), Moghaddam *et al.* (2004), Gao *et al.* (2015) and Davy *et al.* (1977).[2,3,6,7]

<u>3g-*o*. 2-Fluorophenyl benzylsulfonate</u>

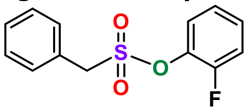

Prepared according to the general procedure described above using 2-fluorophenol as the phenol. White solid. Yield: 129 mg (95%)

HRMS: *m/z* $C_{13}H_{11}FNaO_3S^+$ ($[M+Na]^+$): calculated 289.0305, found 289.0306.

^{1}H NMR (CD_3CN, 400 MHz): δ 7.56 – 7.41 (m, 5H), 7.40 – 7.28 (m, 2H), 7.29 – 7.17 (m, 2H), 4.76 (s, 2H). **^{13}C NMR (CD_3CN, 101 MHz):** δ 155.5 (d, *J* = 242.4 Hz), 137.7 (d, *J* = 10.1 Hz), 132.1, 130.3, 129.9, 129.8 (d, *J* = 8.1 Hz), 128.6, 126.2 (d, *J* = 3.8 Hz), 126.16, 125.63, 57.8 (d, *J* = 1.4 Hz). **^{19}F NMR (CD_3CN, 376 MHz, decoupled):** δ -129.28.

$\tilde{\nu}_{max}$: 3068, 3036, 2931, 1599, 1497, 1458, 1373, 1252, 1159, 1099, 1028 cm^{-1}.

3g-*m*. 3-Fluorophenyl benzylsulfonate

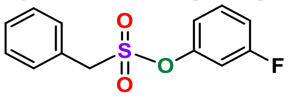

Prepared according to the general procedure described above using 3-fluorophenol as the phenol. NMR yield: 100%

^{1}H NMR (CD_3CN, 400 MHz): δ 7.51 – 7.42 (m, 7H), 7.06 (dd, *J* = 8.3, 2.3 Hz, 1H), 6.99 (dt, *J* = 9.6, 2.4 Hz, 1H), 4.71 (s, 2H).

Product was previously reported in Davy *et al.* (1977).[7]

3g-*p*. 4-Fluorophenyl benzylsulfonate

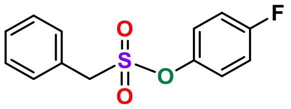

Prepared according to the general procedure described above using 4-fluorophenol as the phenol. NMR yield: 100%

^{1}H NMR (CD_3CN, 400 MHz): δ 7.51 – 7.41 (m, 5H), 7.25 – 7.13 (m, 4H), 4.67 (s, 2H).

Product was previously reported in Alonso *et al.* (2005).[2]

3h-*o*. 2-Bromophenyl benzylsulfonate

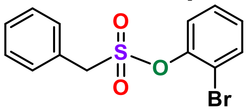

Prepared according to the general procedure described above using 2-bromophenol as the phenol. White solid. NMR yield: 100%

HRMS: *m/z* $C_{13}H_{11}BrNaO_3S^+$ ([M+Na]$^+$): calculated 348.9504, found 348.9506.

^{1}H NMR (CD_3CN, 400 MHz): δ 7.71 (dd, *J* = 8.0, 1.6 Hz, 1H), 7.55 – 7.50 (m, 2H), 7.48 – 7.42 (m, 3H), 7.43 – 7.36 (m, 1H), 7.33 – 7.17 (m, 2H), 4.80 (s, 2H). **^{13}C NMR (CD_3CN, 101 MHz):** δ 147.7, 135.0, 132.1, 130.3, 130.2, 129.9, 129.7, 128.6, 124.9, 117.0, 58.5.

$\bar{\nu}_{max}$: 3088, 3063, 3034, 2982, 2933, 1464, 1371, 1207, 1167, 1155, 1142, 1041 cm^{-1}.

3h-*m*. 3-Bromophenyl benzylsulfonate

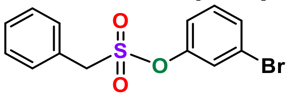

Prepared according to the general procedure described above using 3-bromophenol as the phenol. White solid. NMR yield: 100%

HRMS: *m/z* $C_{13}H_{11}BrNaO_3S^+$ ([M+Na]$^+$): calculated 348.9504, found 348.9507.

^{1}H NMR (CD_3CN, 400 MHz): δ 7.57 – 7.40 (m, 6H), 7.41 – 7.29 (m, 2H), 7.21 (dd, *J* = 6.7, 2.3 Hz, 1H), 4.71 (s, 2H). **^{13}C NMR (CD_3CN, 101 MHz):** δ 150.8, 132.4, 132.0, 131.4, 130.2, 129.9, 128.7, 126.3, 123.1, 122.3, 57.4.

$\bar{\nu}_{max}$: 3093, 3064, 3034, 2983, 2929, 1579, 1466, 1358, 1205, 1186, 1149 cm^{-1}.

3h-*p*. 4-Bromophenyl benzylsulfonate

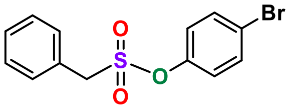

Prepared according to the general procedure described above using 4-bromophenol as the phenol. NMR yield: 100%

^{1}H NMR (CD_3CN, 400 MHz): δ 7.58 (dt, *J* = 9.1, 3.4 Hz, 2H), 7.51 – 7.42 (m, 5H), 7.12 (dt, *J* = 8.9, 3.4 Hz, 2H), 4.68 (s, 2H).

Product was previously reported in Alonso *et al.* (2005), Truce *et al.* (1970), and Smedley *et al.* (2022).[2,4,8]

3i. 4-Iodophenyl benzylsulfonate

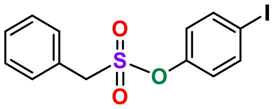

Prepared according to the general procedure described above using 4-iodophenol as the phenol. White solid. NMR yield: 100%

HRMS: *m/z* $C_{13}H_{11}INaO_3S^+$ ($[M+Na]^+$): calculated 396.9366, found 396.9367.

^{1}H NMR (CD_3CN, 400 MHz): δ 7.78 (d, *J* = 8.7 Hz, 2H), 7.53 – 7.41 (m, 5H), 6.99 (d, *J* = 8.7 Hz, 2H), 4.68 (s, 2H). **^{13}C NMR (CD_3CN, 101 MHz):** δ 150.4, 140.1, 132.0, 130.2, 129.9, 128.7, 125.3, 92.1, 57.2.

$\bar{\upsilon}_{max}$: 3084, 3060, 3005, 2955, 1572, 1477, 1354, 1209, 1194, 1173, 1151, 1007 cm^{-1}.

3j. 4-(Trifluoromethoxy)phenyl benzylsulfonate

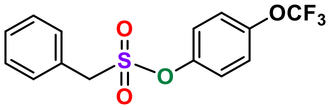

Prepared according to the general procedure described above using 4-(trifluoromethoxy)phenol as the phenol. White solid. Yield: 170 mg (100%)

HRMS: *m/z* $C_{14}H_{15}F_3NO_4S^+$ ($[M+NH_4]^+$): calculated 350.0668, found 350.0659.

^{1}H NMR (CD_3CN, 400 MHz): δ 7.55 – 7.40 (m, 5H), 7.36 (d, *J* = 9.3 Hz, 2H), 7.31 – 7.25 (m, 2H), 4.71 (s, 2H). **^{13}C NMR (CD_3CN, 101 MHz):** δ 148.8, 148.3, 132.0, 130.2, 129.9, 128.7, 124.9, 123.8, 121.4 (q, *J* = 259.3 Hz), 57.28. **^{19}F NMR (CD_3CN, 376 MHz):** δ -58.92.

$\bar{\upsilon}_{max}$: 3076, 2949, 1498, 1354, 1223, 1215, 1146, 1014 cm^{-1}.

3k-*p*. 4-Cyanophenyl benzylsulfonate

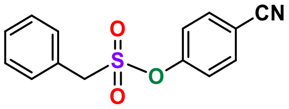

Prepared according to the general procedure described above using 4-cyanophenol as the phenol. White solid. NMR yield: 100%

HRMS: *m/z* $C_{14}H_{12}NO_3S^+$ ($[M+H]^+$): calculated 274.0532, found 274.0527.

^{1}H NMR (CD_3CN, 400 MHz): δ 7.79 (d, *J* = 8.8 Hz, 2H), 7.47 (dd, *J* = 12.4, 3.1 Hz, 5H), 7.33 (d, *J* = 8.8 Hz, 2H), 4.74 (s, 2H). **^{13}C NMR (CD_3CN, 101 MHz):** δ 153.4, 135.4, 132.0, 130.3, 129.9, 128.5, 124.1, 118.8, 111.9, 57.7.

$\bar{\upsilon}_{max}$: 3101, 3063, 2237, 1597, 1493, 1458, 1361, 1151, 1144, 1103, 1016 cm^{-1}.

3l. 3,4,5-Trifluorophenyl benzylsulfonate

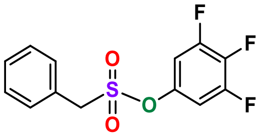

Prepared according to the general procedure described above using 3,4,5-trifluorophenol as the phenol. White solid. NMR yield: 100%

HRMS: *m/z* $C_{13}H_{13}F_3NO_3S^+$ ($[M+NH_4]^+$): calculated 320.0563, found 320.0555.

^{1}H NMR (CD_3CN, 400 MHz): δ 7.50 – 7.43 (m, 5H), 7.06 – 6.97 (m, 2H), 4.73 (s, 2H). **^{13}C NMR (CD_3CN, 101 MHz):** δ 151.9 (ddd, *J* = 249.5, 10.7, 5.2 Hz), 145.0 (td, *J* = 12.8, 4.6 Hz), 140.5 (dd, *J* = 249.3, 15.1 Hz), 132.1, 130.4, 130.0, 128.4, 109.1 (dd, *J* = 25.6, 7.1 Hz), 57.4. **^{19}F NMR (CD_3CN, 376 MHz, decoupled):** δ -134.01, -134.06, -163.71 (t, *J* = 20.0 Hz).

$\bar{\upsilon}_{max}$: 3093, 3007, 2956, 1633, 1514, 1454, 1338, 1228, 1171, 1105, 1051 cm^{-1}.

3m. 4-(Hydroxymethyl)phenyl benzylsulfonate

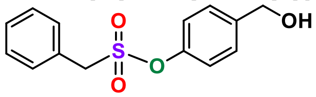

Prepared according to the general procedure described above using 4-hydroxybenzyl alcohol as the phenol. White solid. NMR yield: 100%

HRMS: *m/z* $C_{14}H_{18}NO_4S^+$ ($[M+NH_4]^+$): calculated 296.0951, found 296.0949.

^{1}H NMR (CD_3CN, 400 MHz): δ 7.50 – 7.44 (m, 5H), 7.40 (dt, *J* = 8.5, 2.6 Hz, 2H), 7.17 (dt, *J* = 8.5, 2.8 Hz, 2H), 4.67 (s, 2H), 4.59 (s, 2H). **^{13}C NMR (CD_3CN, 101 MHz):** δ 149.2, 142.5, 132.0, 130.1, 129.9, 129.1, 122.9, 115.6, 63.9, 57.1

$\bar{\upsilon}_{max}$: 3541, 3066, 3036, 2931, 2875, 1595, 1502, 1354, 1142, 1014 cm^{-1}.

3o-*o*. 2-Tert-butylphenyl benzylsulfonate

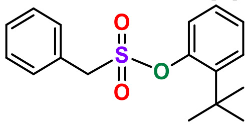

Prepared according to the general procedure described above using 2-tert-butylphenol as the phenol. White solid. NMR yield: 100%

HRMS: *m/z* $C_{17}H_{20}NaO_3S^+$ ($[M+Na]^+$): calculated 327.1025, found 327.1027.

^{1}H NMR (CD_3CN, 400 MHz): 7.64 – 7.37 (m, 6H), 7.38 – 7.27 (m, 1H), 7.26 – 7.20 (m, 2H), 4.80 (s, 2H), 1.36 (s, 9H). **^{13}C NMR (CD_3CN, 101 MHz):** δ 150.0, 142.1, 132.1, 130.2, 129.9, 129.2, 128.8, 128.4, 127.3, 122.2, 59.1, 35.4, 30.7.

$\bar{\upsilon}_{max}$: 3066, 2956, 2872, 1485, 1443, 1354, 1203, 1180, 1147, 1076 cm^{-1}.

3o-*p*. 4-Tert-butylphenyl benzylsulfonate

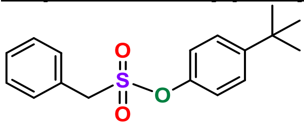

Prepared according to the general procedure described above using 4-tert-butylphenol as the phenol. White solid. NMR yield: 100%

HRMS: *m/z* $C_{17}H_{20}NaO_3S^+$ ($[M+Na]^+$): calculated 327.1025, found 327.1027.

^{1}H NMR (CD_3CN, 400 MHz): δ 7.52 – 7.41 (m, 7H), 7.12 (d, *J* = 8.8 Hz, 2H), 4.66 (s, 2H), 1.32 (s, 9H). **^{13}C NMR (CD_3CN, 101 MHz):** δ 151.5, 148.1, 132.0, 130.1, 129.8, 129.0, 127.9, 122.6, 57.2, 35.3, 31.5.

$\bar{\upsilon}_{max}$: 3061, 2960, 2870, 1502, 1367, 1205, 1176, 1149, 1016 cm^{-1}.

3p. Pyridin-3-yl benzylsulfonate

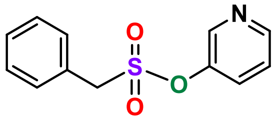

Prepared according to the general procedure described above using 3-hydroxypyridine as the phenol. White solid. NMR yield: 100%

HRMS: *m/z* $C_{12}H_{12}NO_3S^+$ ($[M+H]^+$): calculated 250.0532, found 250.0533.

^{1}H NMR (CD_3CN, 400 MHz): δ 8.53 (dd, *J* = 4.7, 1.4 Hz, 1H), 8.44 (d, *J* = 2.2 Hz, 1H), 7.58 (ddd, *J* = 8.4, 2.8, 1.4 Hz, 1H), 7.53 – 7.39 (m, 6H), 4.75 (s, 2H). **^{13}C NMR (CD_3CN, 101 MHz):** δ 149.3, 147.5, 144.6, 132.1, 130.7, 130.3, 129.9, 128.6, 125.6, 57.4.

$\bar{\upsilon}_{max}$: 3095, 3055, 2980, 2926, 1572, 1473, 1421, 1369, 1207, 1188, 1159, 1022 cm^{-1}.

3q. 4-(Trifluoromethyl)phenyl benzylsulfonate

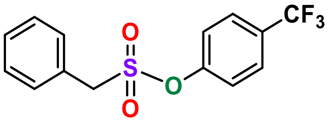

Prepared according to the general procedure described above using 4-(trifluoromethyl)phenol as the phenol. White solid. Yield: 155 mg (96%)

HRMS: *m/z* $C_{16}H_{14}F_3NNaO_3S^+$ ([M+ACN+Na]$^+$): calculated 380.0539, found 380.0541.

^{1}H NMR (CD$_3$CN, 400 MHz): δ 7.76 (d, *J* = 8.5 Hz, 2H), 7.53 – 7.41 (m, 5H), 7.37 (d, *J* = 8.5 Hz, 2H), 4.74 (s, 2H). **^{13}C NMR (CD$_3$CN, 101 MHz):** δ 153.0, 132.1, 130.3, 129.9, 129.6 (q, *J* = 34.3 Hz), 128.6, 128.4 (q, *J* = 3.8 Hz), 124.9 (q, *J* = 270.8 Hz) 123.8, 57.5. **^{19}F NMR (CD$_3$CN, 376 MHz, decoupled):** δ -62.89.

$\tilde{\nu}_{max}$: 3064, 3005, 2953, 1610, 1510, 1327, 1215, 1151, 1119, 1066, 1014 cm^{-1}.

3r. Naphthalen-2-yl benzylsulfonate

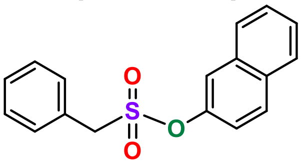

Prepared according to the general procedure described above using 2-naphthol as the phenol. NMR yield: 100%

^{1}H NMR (CD$_3$CN, 400 MHz): δ 8.00 – 7.86 (m, 3H), 7.72 (d, *J* = 2.5 Hz, 1H), 7.61 – 7.54 (m, 2H), 7.54 – 7.48 (m, 2H), 7.48 – 7.42 (m, 3H), 7.34 (dd, *J* = 8.9, 2.5 Hz, 1H), 4.74 (s, 2H).

Product was previously reported in Bahrami *et al.* (2012).[9]

3s. 4-acetamidophenyl benzylsulfonate

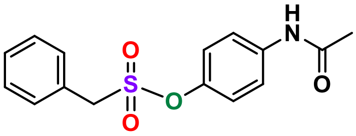

Prepared according to the general procedure described above using acetaminophen as the phenol. White solid. NMR yield: 100%

HRMS: *m/z* $C_{15}H_{15}NNaO_4S^+$ ([M+Na]$^+$): calculated 328.0614, found 328.0614.

^{1}H NMR (CD$_3$CN with 37.5% CD$_3$OD, 400 MHz): δ 7.57 (dt, *J* = 8.9, 3.6 Hz, 2H), 7.48 – 7.38 (m, 5H), 7.12 (dt, *J* = 8.9, 3.4 Hz, 2H), 4.66 (s, 2H), 2.06 (s, 3H). **^{13}C NMR (CD$_3$CN with 37.5% CD$_3$OD, 101 MHz):** δ 171.4, 146.5, 139.4, 132.5, 130.5, 130.3, 129.5, 123.9, 122.3, 57.4, 24.4.

$\tilde{\nu}_{max}$: 3313, 3074, 2995, 2941, 2447, 1651, 1595, 1508, 1362, 1190, 1147, 1018 cm^{-1}.

3u. 3,5-Bis(trifluoromethyl)phenyl benzylsulfonate

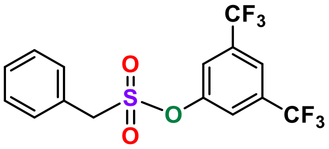

Prepared according to the general procedure described above using 3,5-bis(trifluoromethyl)phenol as the phenol. White solid. Yield: 167 mg (85%)

HRMS: *m/z* $C_{15}H_{14}F_6NO_3S^+$ ([M+NH$_4$]$^+$): calculated 402.0593, found 402.0582.

^{1}H NMR (CD$_3$CN, 400 MHz): δ 7.97 (s, 1H), 7.67 (s, 2H), 7.55 – 7.39 (m, 5H), 4.81 (s, 2H). **^{13}C NMR (CD$_3$CN, 101 MHz):** δ 151.1, 133.8 (q, *J* = 33.9 Hz), 132.1, 130.5, 130.0, 128.3, 124.4 (q, *J* = 4.0 Hz), 122.2 (hept, *J* = 3.6 Hz), 121.1 (q, *J* = 272.7 Hz), 57.9. **^{19}F NMR (CD$_3$CN, 376 MHz, decoupled):** δ -63.49.

$\tilde{\nu}_{max}$: 3086, 2989, 2933, 1614, 1458, 1360, 1275, 1128 cm^{-1}.

3v. Naphthalen-1-yl benzylsulfonate

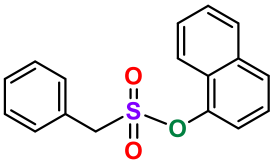

Prepared according to the general procedure described above using 1-naphthol as the phenol. NMR yield: 100%

^{1}H NMR (CD_3CN, 400 MHz): δ 7.98 – 7.92 (m, 2H), 7.87 (d, *J* = 8.3, 1.1 Hz, 1H), 7.60 – 7.53 (m, 4H), 7.50 – 7.44 (m, 4H), 7.34 (dd, *J* = 7.7, 1.2 Hz, 1H), 4.85 (s, 2H).

Product was previously reported in Alonso *et al.* (2005) & Moghaddam *et al.* (2004).[2,3]

3w. methyl 4-((benzylsulfonyl)oxy)benzoate

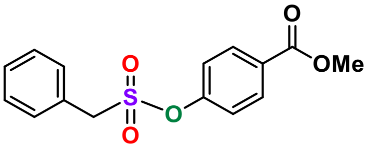

Prepared according to the general procedure described above using methyl 4-hydroxybenzoate as the phenol. White solid. NMR yield: 100%

HRMS: *m/z* $C_{15}H_{15}O_5S^+$ ($[M+H]^+$): calculated 307.0635, found 307.0632.

^{1}H NMR (CD_3CN, 400 MHz): δ 8.05 (d, *J* = 8.8 Hz, 2H), 7.52 – 7.41 (m, 5H), 7.29 (d, *J* = 8.8 Hz, 2H), 4.72 (s, 2H), 3.88 (s, 3H). **^{13}C NMR (CD_3CN, 101 MHz):** δ 166.7, 153.7, 132.3, 132.0, 130.2, 130.1, 129.9, 128.7, 123.1, 57.5, 53.0.

$\bar{\upsilon}_{max}$: 3007, 2955, 1716, 1602, 1504, 1433, 1356, 1277, 1215, 1157, 1153, 1097, 1018 cm^{-1}.

3y. 4-carbamoylphenyl phenylmethanesulfonate

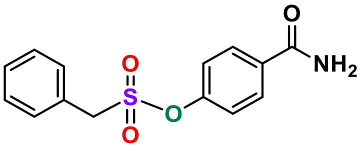

Prepared according to the description of the BTMG/DBU reaction provided above. **3y** was isolated by precipitation from the reaction mixture using an acetone/water mixture. The solid was collected using vacuum filtration, washed with an acetone/water mixture, followed by hexane, and then dried under vacuum. White solid.

HRMS: *m/z* $C_{14}H_{12}NO_4S^-$ ($[M-H]^-$): calculated 290.0493, found 290.0497

^{1}H NMR (MeOD with 50% CD_3CN, 400 MHz): δ 7.93 (dt, *J* = 8.9, 2.8 Hz, 2H), 7.55 – 7.49 (m, 2H), 7.49 – 7.43 (m, 3H), 7.29 (dt, *J* = 8.8, 2.8 Hz, 2H), 4.79 (s, 2H). **^{13}C NMR (MeOD with 50% CD_3CN, 101 MHz):** δ 170.9, 153.6, 134.3, 132.7, 131.1, 130.8, 130.4, 129.5, 123.6. (Aliphatic peak is hidden underneath MeOD solvent peak.)

3aa. 4-((4-Isopropoxyphenyl)sulfonyl)phenyl benzylsulfonate

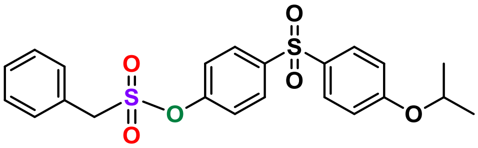

Prepared according to the general procedure described above using 4-((4-isopropoxyphenyl)sulfonyl)phenol as the phenol. White solid. NMR yield: 100%

HRMS: *m/z* $C_{22}H_{23}O_6S_2^+$ ($[M+H]^+$): calculated 447.0931, found 447.0939.

^{1}H NMR (CD_3CN, 400 MHz): δ 7.95 (d, *J* = 8.8 Hz, 2H), 7.84 (d, *J* = 9.0 Hz, 2H), 7.49 – 7.36 (m, 5H), 7.33 (d, *J* = 8.8 Hz, 2H), 7.02 (d, *J* = 9.0 Hz, 2H), 4.71 (s, 2H), 4.68 (hept, 1H), 1.29 (d, *J* = 6.0 Hz, 6H).

^{13}C NMR (CD_3CN, 101 MHz): δ 163.3, 153.5, 142.2, 132.9, 132.0, 130.9, 130.4, 130.3, 129.9, 128.5, 124.1, 117.1, 71.6, 57.6, 22.0.

$\tilde{\upsilon}_{max}$: 3097, 3066, 2980, 2933, 1589, 1489, 1373, 1319, 1292, 1259, 1144, 1101, 1014 cm^{-1}.

3ab. 1,3-phenylene-bis(benzylsulfonate)

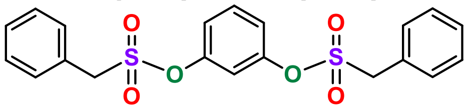

Prepared according to the general procedure described above, with 2 equiv of NaH, using resorcinol as the phenol. White solid. NMR yield: 100%

HRMS: *m/z* $C_{20}H_{22}NO_6S_2^+$ ([M+NH$_4$]$^+$): calculated 436.0883, found 436.0875.

^{1}H NMR (CD_3CN, 400 MHz): δ 7.52 – 7.42 (m, 11H), 7.21 (d, *J* = 2.3 Hz, 1H), 7.19 (d, *J* = 2.3 Hz, 1H), 7.03 (t, *J* = 2.3 Hz, 1H), 4.70 (s, 4H). **^{13}C NMR (CD_3CN, 101 MHz):** δ 150.8, 132.0, 130.3, 129.9, 128.7, 122.0, 117.4, 57.4.

$\tilde{\upsilon}_{max}$: 3066, 3037, 2929, 1595, 1477, 1356, 1171, 1101 cm^{-1}.

3ac. Propane-2,2-diylbis(4,1-phenylene) bis(benzylsulfonate)

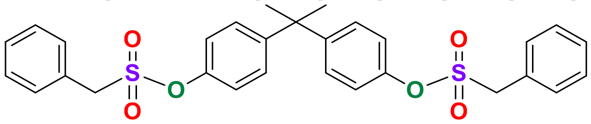

Prepared according to the general procedure described above, with 2 equiv of NaH, using bisphenol A as the phenol. White solid. NMR yield: 100%

HRMS: *m/z* $C_{29}H_{32}NO_6S_2^+$ ([M+NH$_4$]$^+$): calculated 554.1666, found 554.1672.

^{1}H NMR (CD_3CN, 400 MHz): δ 7.57 – 7.35 (m, 10H), 7.28 (d, *J* = 8.8 Hz, 4H), 7.11 (d, *J* = 8.8 Hz, 4H), 4.66 (s, 4H), 1.67 (s, 6H). **^{13}C NMR (CD_3CN, 101 MHz):** δ 150.4, 148.4, 132.0, 130.1, 129.8, 129.3, 129.0, 122.6, 57.2, 43.4, 30.9.

$\tilde{\upsilon}_{max}$: 3066, 3036, 2970, 1498, 1365, 1207, 1176, 1146, 1016 cm^{-1}.

3ae. 2,6-Di-tert-butyl-4-methylphenyl benzylsulfonate

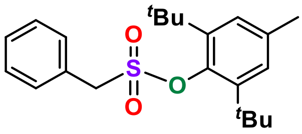

Prepared according to the general procedure described above using 2,6-di-tert-butyl-4-methylphenol as the phenol. White solid. NMR yield: 100%

HRMS: *m/z* $C_{22}H_{34}NO_3S^+$ ([M+NH$_4$]$^+$): calculated 392.2254, found 392.2255.

^{1}H NMR (CD_3CN, 400 MHz): δ 7.58 – 7.42 (m, 5H), 7.22 (s, 2H), 4.75 (s, 2H), 2.31 (s, 3H), 1.39 (s, 18H). **^{13}C NMR (CD_3CN, 101 MHz):** δ 145.9, 136.2, 132.4, 130.1, 130.0, 129.7, 128.6, 58.1, 37.6, 33.2, 30.7, 20.9.

$\tilde{\upsilon}_{max}$: 3039, 2958, 1670, 1595, 1346, 1267, 1178, 1157, 1088 cm^{-1}.

3af. 4-(Dimethylamino)phenyl benzylsulfonate

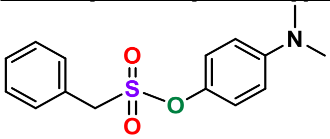

Prepared according to the general procedure described above using 4-(dimethylamino)phenol as the phenol. Pinkish solid. NMR yield: 100%

HRMS: *m/z* $C_{15}H_{18}NO_3S^+$ ([M+H]$^+$): calculated 292.1002, found 292.1001.

^{1}H NMR (CD_3CN, 400 MHz): δ 7.52 – 7.40 (m, 5H), 7.05 (d, *J* = 9.2 Hz, 2H), 6.72 (d, *J* = 9.2 Hz, 2H), 4.59 (s, 2H), 2.92 (s, 6H). **^{13}C NMR (CD_3CN, 101 MHz):** δ 150.80, 140.71, 131.97, 129.98, 129.79, 129.20, 123.61, 113.72, 56.61, 40.88.

$\tilde{\nu}_{max}$: 3070, 3037, 2985, 2937, 2808, 1605, 1514, 1336, 1198, 1174, 1142, 1070 cm^{-1}.

5a. 1-Butoxy benzylsulfonate

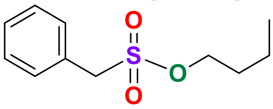

Prepared according to the general procedure described above using 1-butanol as the alcohol. White solid. NMR yield: 100%

HRMS: *m/z* $C_{11}H_{20}NO_3S^+$ ($[M+NH_4]^+$): calculated 246.1158, found 246.1156.

^{1}H NMR (CD_3CN, 400 MHz): δ 7.42 (m, 5H), 4.44 (s, 2H), 4.16 (t, *J* = 6.5 Hz, 2H), 1.64 (p, 2H), 1.37 (sext, *J* = 7.5 Hz, 2H), 0.91 (t, *J* = 7.4 Hz, 3H). **^{13}C NMR (CD_3CN, 101 MHz):** δ 131.77, 129.85, 129.76, 129.69, 72.28, 56.23, 31.86, 19.36, 13.77.

$\tilde{\nu}_{max}$: 3066, 3036, 2962, 2875, 1497, 1456, 1352, 1203, 1169, 1140 cm^{-1}.

5b. 2-Butoxy benzylsulfonate

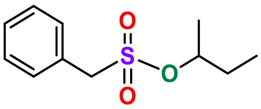

Prepared according to the general procedure described above using 2-butanol as the alcohol. White solid. NMR yield: 100%

HRMS: *m/z* $C_{11}H_{20}NO_3S^+$ ($[M+NH_4]^+$): calculated 246.1158, found 246.1156.

^{1}H NMR (CD_3CN, 400 MHz): δ 7.47 – 7.37 (m, 5H), 4.68 (sext, *J* = 6.2 Hz, 1H), 4.42 (s, 2H), 1.71 – 1.59 (m, 2H), 1.31 (d, *J* = 6.3 Hz, 3H), 0.90 (t, *J* = 7.4 Hz, 3H). **^{13}C NMR (CD_3CN, 101 MHz):** δ 131.8, 130.0, 129.7, 129.6, 83.2, 57.5, 30.3, 20.8, 9.6.

$\tilde{\nu}_{max}$: 3066, 3034, 2978, 2935, 2881, 1497, 1456, 1336, 1171 cm^{-1}.

5d. benzyl benzylsulfonate

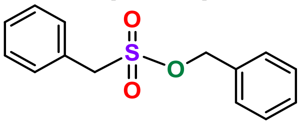

Prepared according to the general procedure described above using benzyl alcohol as the alcohol. NMR yield: 100%

^{1}H NMR (CD_3CN, 400 MHz): δ 7.46 – 7.33 (m, 10H), 5.21 (s, 2H), 4.57 (s, 2H).

Product was previously reported in Dai *et al.* (2021).[10]

5e. cyclohexyl benzylsulfonate

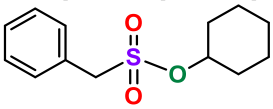

Prepared according to the general procedure described above using cyclohexanol as the alcohol. NMR yield: 100%

^{1}H NMR (CD_3CN, 400 MHz): δ 7.50 – 7.35 (m, 5H), 4.59 (hept, *J* = 8.7, 3.9 Hz, 1H), 4.42 (s, 2H), 1.93 – 1.86 (m, 2H), 1.76 – 1.65 (m, 2H), 1.61 – 1.46 (m, 3H), 1.43 – 1.20 (m, 3H).

Product was previously reported in Alonso *et al.* (2005).[2]

5f. propane-1,2,3-triyl tris(benzylsulfonate)

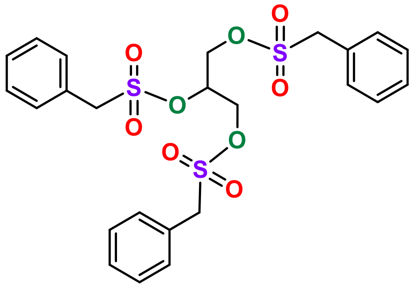

Prepared according to the general procedure described above, using 3 equiv of NaH and 3 equiv of **1**, and using glycerol as the alcohol. White solid. Yield: 95 mg (100%)

HRMS: *m/z* $C_{24}H_{30}NO_9S_3^+$ ($[M+NH_4]^+$): calculated 572.1077, found 572.1074.

^{1}H NMR (CD_3CN, 400 MHz): δ 7.49 – 7.37 (m, 15H), 5.01 (tt, *J* = 5.6, 3.7 Hz, 1H), 4.51 (s, 4H), 4.45 (s, 2H), 4.30 (qd, *J* = 11.8, 4.7 Hz, 4H). **^{13}C NMR (CD_3CN, 101 MHz):** δ 131.92, 131.87, 130.07, 130.06, 129.89, 129.85, 129.08, 128.90, 77.14, 68.75, 57.76, 56.78.

$\bar{\upsilon}_{max}$: 3066, 3034, 3007, 2955, 1493, 1456, 1358, 1169, 1090, 1014 cm^{-1}.

7a. 2-isopropyl-5-methylphenyl benzylsulfonate

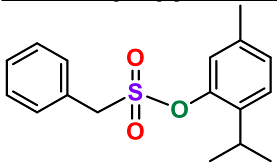

Prepared according to the general procedure described above using thymol as the phenol. White solid. NMR yield: 100%

HRMS: *m/z* $C_{17}H_{24}NO_3S^+$ ($[M+NH_4]^+$): calculated 322.1471, found 322.1470.

^{1}H NMR (CD_3CN, 400 MHz): δ 7.59 – 7.41 (m, 5H), 7.26 (d, *J* = 8.0 Hz, 1H), 7.11 (d, *J* = 8.0 Hz, 1H), 6.88 (s, 1H), 4.75 (s, 2H), 3.06 (hept, *J* = 6.9 Hz, 1H), 2.27 (s, 3H), 1.11 (d, *J* = 6.9 Hz, 6H). **^{13}C NMR (CD_3CN, 101 MHz):** δ 147.32, 139.65, 138.28, 132.11, 130.11, 129.84, 129.23, 129.17, 128.03, 123.31, 57.90, 27.31, 23.45, 20.79.

$\bar{\upsilon}_{max}$: 3064, 3034, 2962, 2929, 2872, 1620, 1502, 1456, 1352, 1234, 1173, 1142, 1076 cm^{-1}.

7b. benzo[*d*][1,3]dioxol-5-yl benzylsulfonate

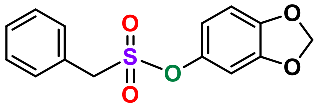

Prepared according to the general procedure described above using sesamol as the phenol. White solid. NMR yield: 100%

HRMS: *m/z* $C_{14}H_{16}NO_5S^+$ ($[M+NH_4]^+$): calculated 310.0744, found 310.0744.

^{1}H NMR (CD_3CN, 400 MHz): δ 7.53 – 7.40 (m, 5H), 6.84 (d, *J* = 8.4 Hz, 1H), 6.75 – 6.66 (m, 2H), 6.02 (s, 2H), 4.65 (s, 2H). **^{13}C NMR (CD_3CN, 101 MHz):** δ 149.4, 147.7, 144.4, 132.0, 130.1, 129.8, 128.9, 116.0, 109.0, 104.9, 103.6, 56.9.

$\bar{\upsilon}_{max}$: 3064, 2985, 2897, 1633, 1610, 1479, 1354, 1246, 1157, 1111, 1090, 1034 cm^{-1}.

7c. 4-Allyl-2-methoxyphenyl benzylsulfonate

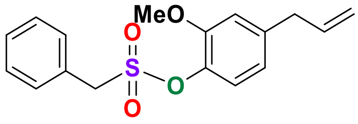

Prepared according to the general procedure described above using eugenol as the phenol. White solid. NMR yield: 100%

HRMS: *m/z* $C_{17}H_{19}O_4S^+$ ($[M+H]^+$): calculated 319.0999, found 319.1001.

^{1}H NMR (CD_3CN, 400 MHz): δ 7.57 – 7.35 (m, 5H), 7.07 (d, *J* = 8.2 Hz, 1H), 6.99 (d, *J* = 2.0 Hz, 1H), 6.79 (dd, *J* = 8.2, 2.0 Hz, 1H), 6.00 (ddt, *J* = 16.9, 10.0, 6.8 Hz, 1H), 5.14 (dd, *J* = 17.1, 1.8 Hz, 1H), 5.09 (d, *J* = 10.0 Hz, 1H), 4.68 (s, 2H), 3.88 (s, 3H), 3.40 (d, *J* = 6.7 Hz, 2H). **^{13}C NMR (CD_3CN, 101 MHz):** δ 152.6, 142.1, 138.2, 137.8, 132.1, 130.0, 129.7, 129.3, 124.8, 121.6, 116.7, 114.5, 58.0, 56.7, 40.5.

$\tilde{\nu}_{max}$: 3066, 3007, 2978, 2939, 2839, 1601, 1504, 1456, 1381, 1367, 1267, 1169, 1109, 1113, 1030 cm^{-1}.

7e. 4-(2-hydroxyethyl)phenyl benzylsulfonate

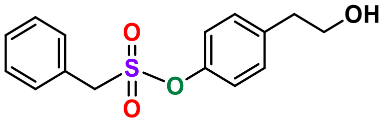

Prepared according to the general procedure described above, using 2 equiv of NaH, and using 2-(4-hydroxyphenyl)ethanol as the phenol. White solid. NMR yield: 100%

HRMS: *m/z* $C_{15}H_{20}NO_4S^+$ ($[M+NH_4]^+$): calculated 310.1108, found 310.1110.

^{1}H NMR ($CDCl_3$, 400 MHz): δ 7.38 – 7.29 (m, 5H), 7.01 (dt, *J* = 8.7, 2.6 Hz, 2H), 6.76 (dt, *J* = 8.5, 2.9 Hz, 2H), 4.26 (s, 2H), 4.16 (t, *J* = 7.0 Hz, 2H), 2.86 (t, *J* = 7.0 Hz, 2H). **^{13}C NMR ($CDCl_3$, 101 MHz):** δ 154.7, 130.8, 130.4, 129.2, 129.0, 128.5, 127.9, 115.6, 71.6, 57.0, 35.0.

$\tilde{\nu}_{max}$: 3066, 3036, 2926, 2854, 1734, 1502, 1456, 1356, 1238, 1171, 1144 cm^{-1}.

7g. 4-(3-oxobutyl)phenyl benzylsulfonate

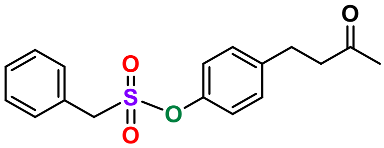

Prepared according to the general procedure described above using 4-(4-hydroxyphenyl)-2-butanone as the phenol. White solid. NMR yield: 100%

HRMS: *m/z* $C_{17}H_{19}O_4S^+$ ($[M+H]^+$): calculated 319.0999, found 319.1000.

^{1}H NMR (CD_3CN, 400 MHz): δ 7.52 – 7.41 (m, 5H), 7.26 (dt, *J* = 8.8, 2.8 Hz, 2H), 7.11 (dt, *J* = 8.6, 2.8 Hz, 2H), 4.66 (s, 2H), 2.87 – 2.75 (m, 4H), 2.09 (s, 3H). **^{13}C NMR (CD_3CN, 101 MHz):** δ 208.4, 148.5, 142.0, 132.0, 130.8, 130.1, 129.8, 123.0, 116.1, 57.1, 45.1, 30.1, 29.6.

$\tilde{\nu}_{max}$: 3066, 3036, 2926, 1711, 1502, 1365, 1205, 1173, 1144 cm^{-1}.

7i. β-Estradiol, benzylsulfonyl ester

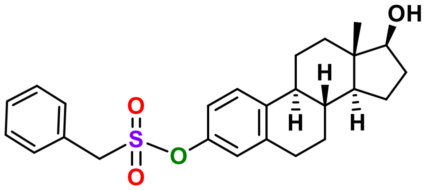

Prepared according to the general procedure described above, using 2 equiv of NaH, and using β-estradiol as the phenol. White solid. NMR yield: 100%

HRMS: *m/z* $C_{25}H_{34}NO_4S^+$ ($[M+NH_4]^+$): calculated 444.2203, found 444.2211.

^{1}H NMR ($CDCl_3$, 400 MHz): δ 7.43 – 7.31 (m, 5H), 7.19 – 7.17 (m, 1H), 6.81 (dd, *J* = 8.6, 2.6 Hz, 1H), 6.75 (d, *J* = 2.6 Hz, 1H), 4.43 (s, 2H), 3.66 (t, *J* = 8.5 Hz, 1H), 2.80 – 2.71 (m, 2H), 2.27 – 2.20 (m, 1H), 2.17 – 2.03 (m, 2H), 1.89 (dt, *J* = 12.5, 3.6 Hz, 1H), 1.85 – 1.78 (m, 1H), 1.70 – 1.58 (m, 1H), 1.38 – 1.08 (m, 8H), 0.71 (s, 3H). **^{13}C NMR ($CDCl_3$, 101 MHz):** δ 147.2, 139.7, 139.1, 131.0, 129.4, 129.1, 127.6, 126.9, 122.1, 119.0, 82.0, 56.8, 50.2, 44.3, 43.4, 38.5, 36.8, 30.8, 29.7, 27.1, 26.3, 23.3, 11.2.

$\tilde{\nu}_{max}$: 3456, 3066, 3034, 2924, 2868, 1720, 1605, 1491, 1456, 1367, 1169, 1130, 1055 cm^{-1}.

Phenyl 4-methylbenzenesulfonate

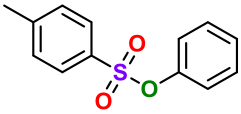

Prepared according to the procedure for reactions with 4-nitrophenyl 4-methylbenzenesulfonate, using phenol as the phenol. White solid. Yield: 127 mg (100%)

^{1}H NMR (CD_3CN, 400 MHz): δ 7.70 (dt, *J* = 8.4, 2.2 Hz, 2H), 7.40 (d, *J* = 8.1 Hz, 2H), 7.37 – 7.26 (m, 3H), 7.04 – 6.98 (m, 2H), 2.43 (s, 3H).

Product was previously reported in many publications, the earliest mention being Gilman *et al.* (1925).[11]

4-Methoxyphenyl 4-methylbenzenesulfonate

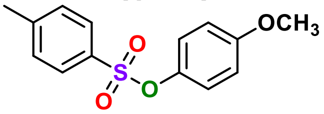

Prepared according to the procedure for reactions with 4-nitrophenyl 4-methylbenzenesulfonate, using 4-methoxyphenol as the phenol. White solid. Yield: 142 mg (100%)

^{1}H NMR (CD_3CN, 400 MHz): δ 7.68 (dt, *J* = 8.4, 2.0 Hz, 2H), 7.41 – 7.36 (m, 2H), 6.91 (dt, *J* = 9.3, 3.5 Hz, 2H), 6.82 (dt, *J* = 9.3, 3.5 Hz, 2H), 3.73 (s, 3H), 2.42 (s, 3H).
Product was previously reported in many publications, the earliest mention being Borrows *et al.* (1949).[12]

4-Cyanophenyl 4-methylbenzenesulfonate

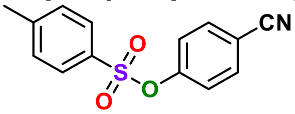

Prepared according to the procedure for reactions with 4-nitrophenyl 4-methylbenzenesulfonate, using 4-cyanophenol as the phenol. White solid. Yield: 119 mg (85%)

^{1}H NMR (CD_3CN, 400 MHz): δ 7.75 – 7.66 (m, 4H), 7.44 – 7.35 (m, 2H), 7.17 (dt, *J* = 8.9, 2.6 Hz, 2H), 2.42 (s, 3H).

Product was previously reported in many publications, the earliest mention being Takikawa *et al.* (1972).[13]

2-butoxy 4-methylbenzenesulfonate

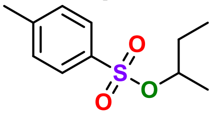

Prepared according to the procedure for reactions with 4-nitrophenyl 4-methylbenzenesulfonate, using 2-butanol as the alcohol. White solid. Yield: 111 mg (95%)

^{1}H NMR (CD_3CN, 400 MHz): δ 7.76 (dt, *J* = 8.4, 1.9 Hz, 2H), 7.47 – 7.36 (m, 2H), 4.53 (h, *J* = 6.2 Hz, 1H), 2.43 (s, 3H), 1.61 – 1.45 (m, 2H), 1.19 (d, *J* = 6.3 Hz, 3H), 0.75 (t, *J* = 7.4 Hz, 3H).

Product was previously reported in many publications, the earliest mention being Kenyon *et al.* (1935).[14]

Polymer 8

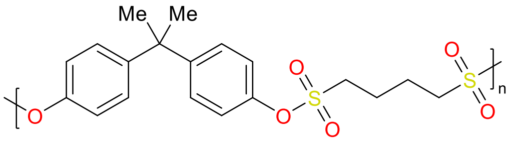

Product was previously reported in Gao *et al.* (2017).[15]
^{1}H NMR (400 MHz, CD_2Cl_2): δ 7.27 (d, *J* = 8.5 Hz, 4H), 7.17 (d, *J* = 8.4 Hz, 4H), 3.31 (s, 4H), 2.14 (s, 5H), 1.67 (s, 6H).

NMR spectra of novel compounds

In both ^{1}H and ^{13}C spectra (400 and 101 MHz, respectively), the solvent (CD_3CN) and water signals are indicated in grey.

<u>3b-*o*. 2-Methylphenyl benzylsulfonate</u>

^{1}H spectrum (400 MHz, CD_3CN):

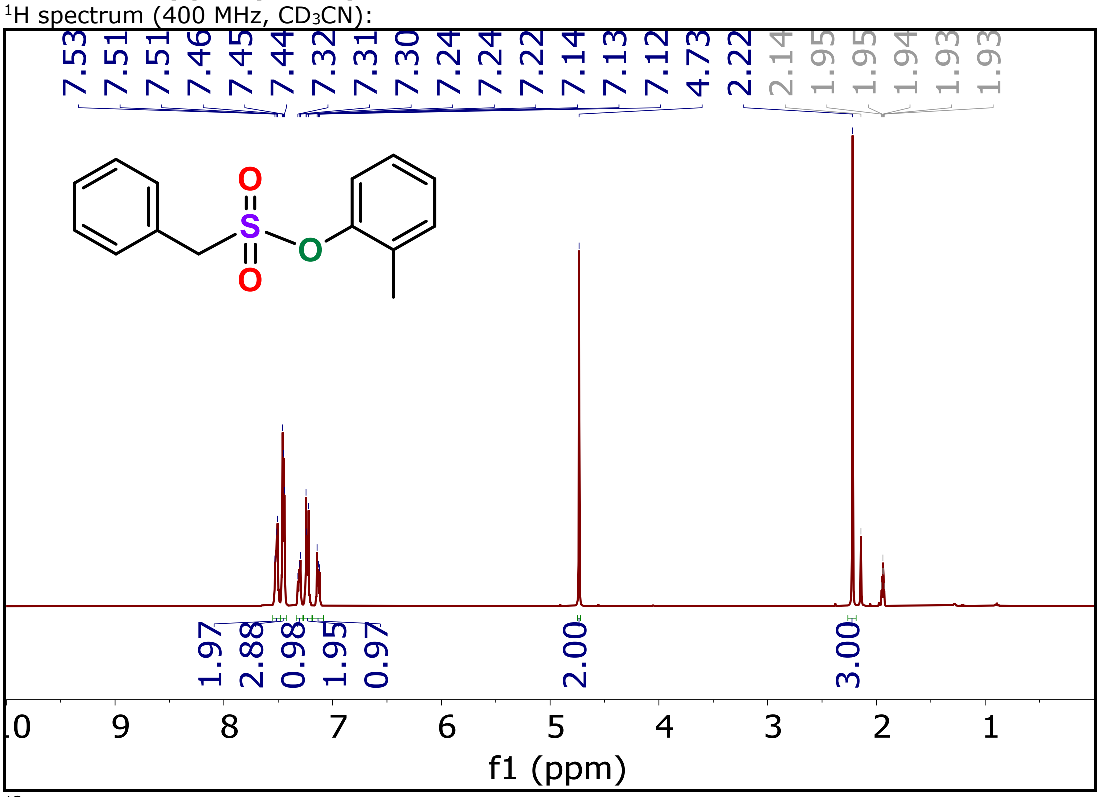

^{13}C spectrum (101 MHz, CD_3CN):

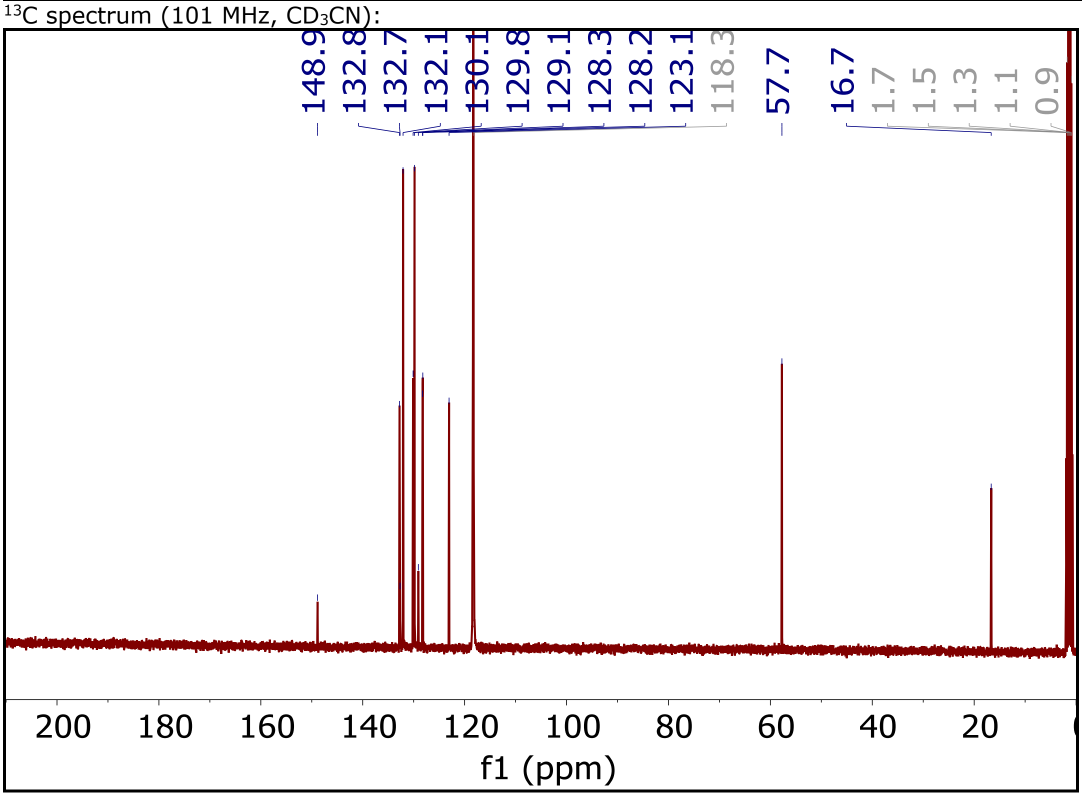

3b-*m*. 3-Methylphenyl benzylsulfonate

^{1}H spectrum (400 MHz, CD_3CN):

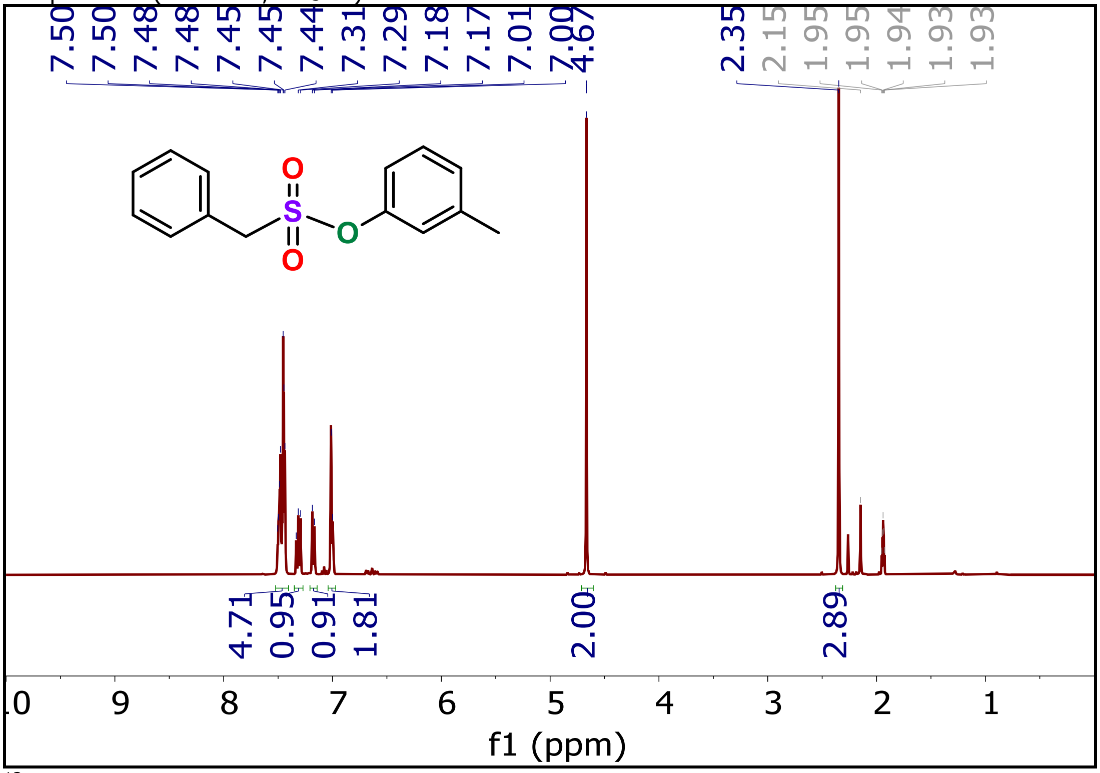

^{13}C spectrum (101 MHz, CD_3CN):

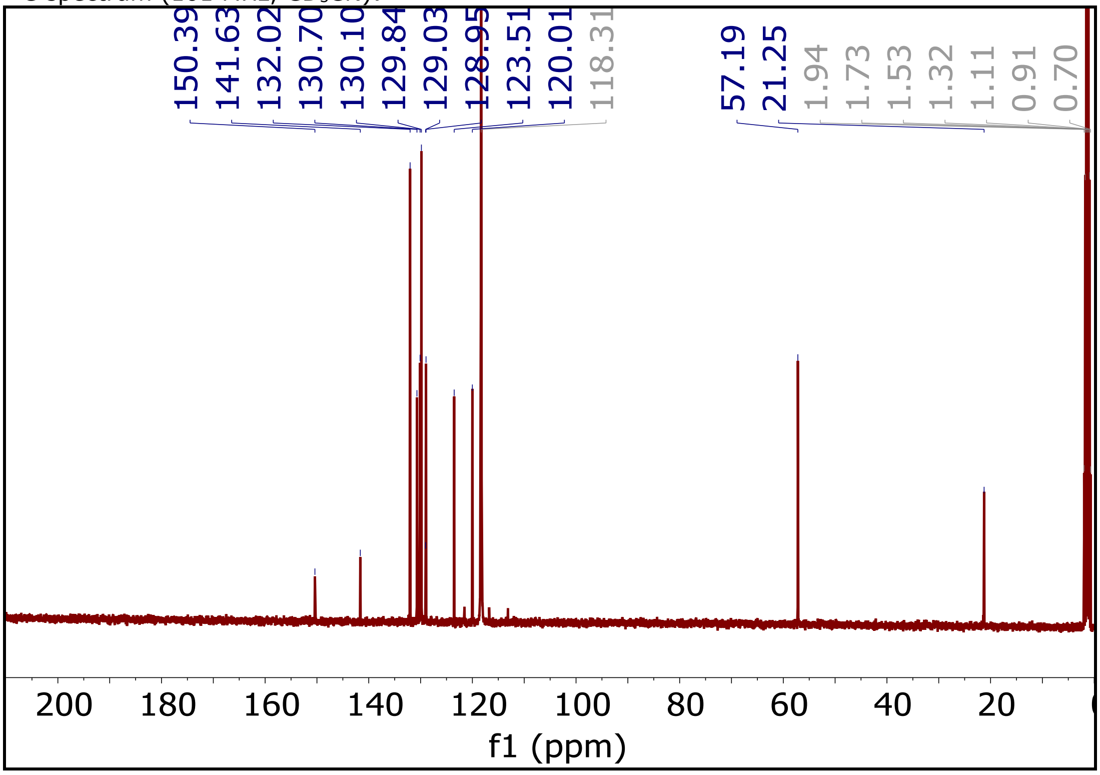

3c. 4-Aminophenyl benzylsulfonate

^{1}H spectrum (400 MHz, CD_3CN with 20% MeOD):

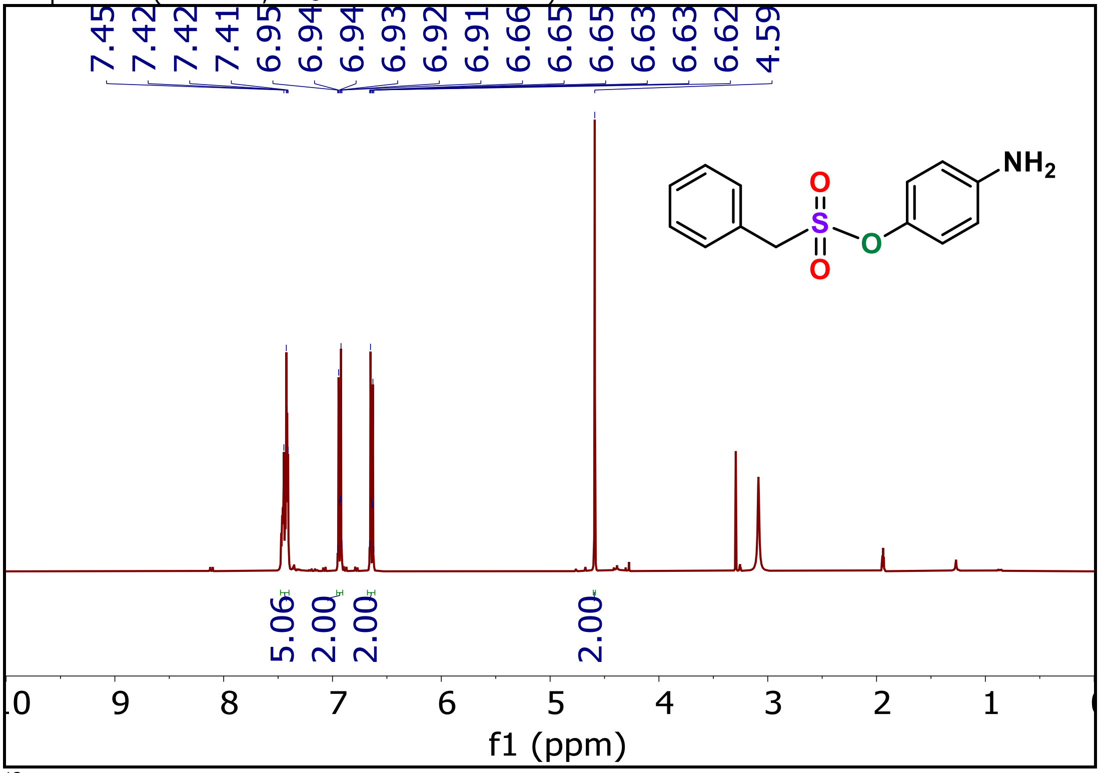

^{13}C spectrum (101 MHz, CD_3CN with 20% MeOD):

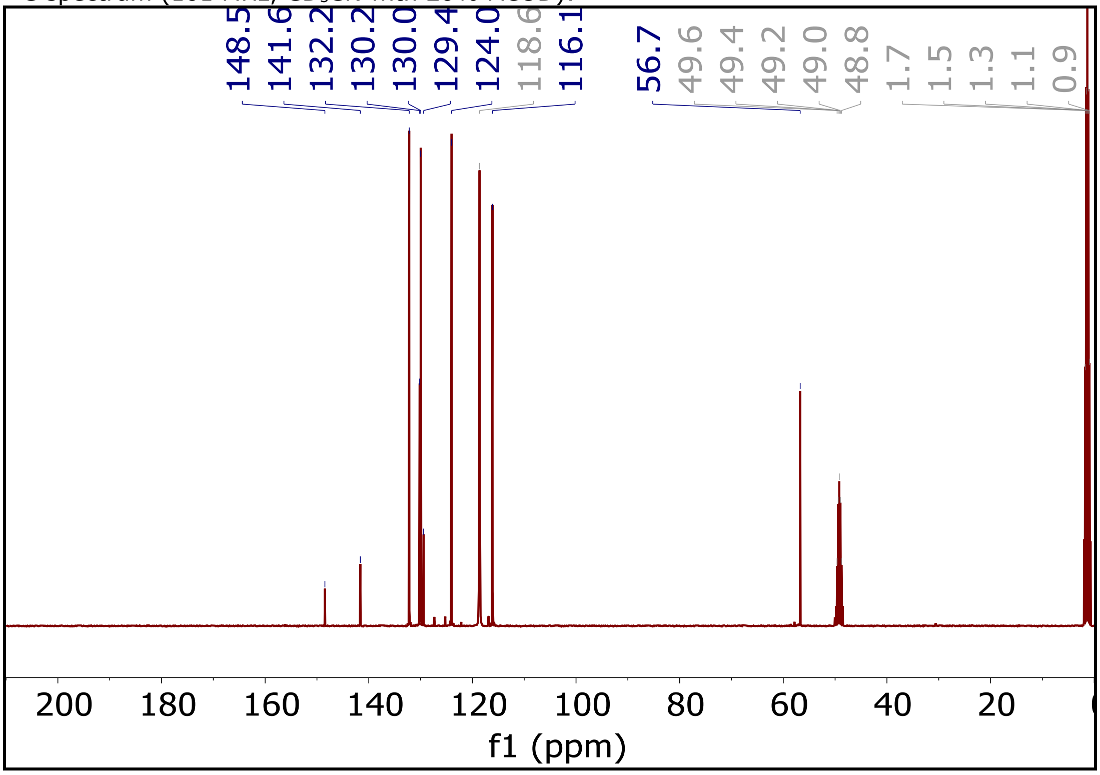

3g-*o*. 2-Fluorophenyl benzylsulfonate

^{1}H spectrum (400 MHz, CD_3CN):

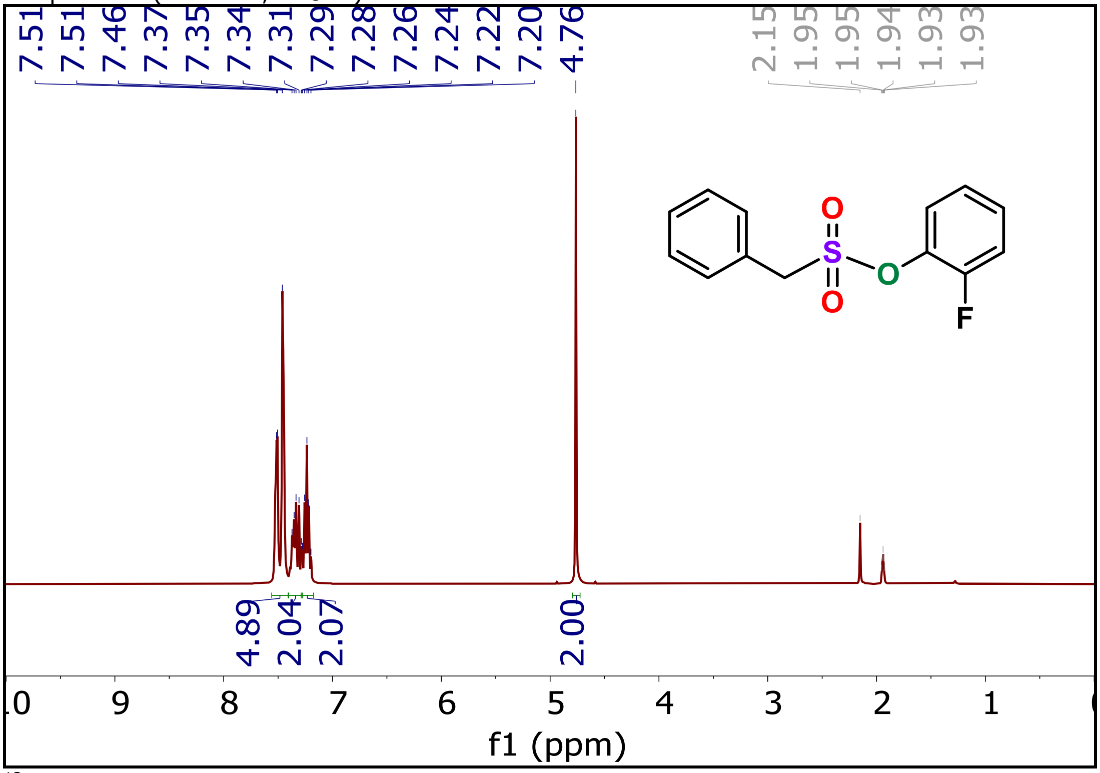

^{13}C spectrum (101 MHz, CD_3CN):

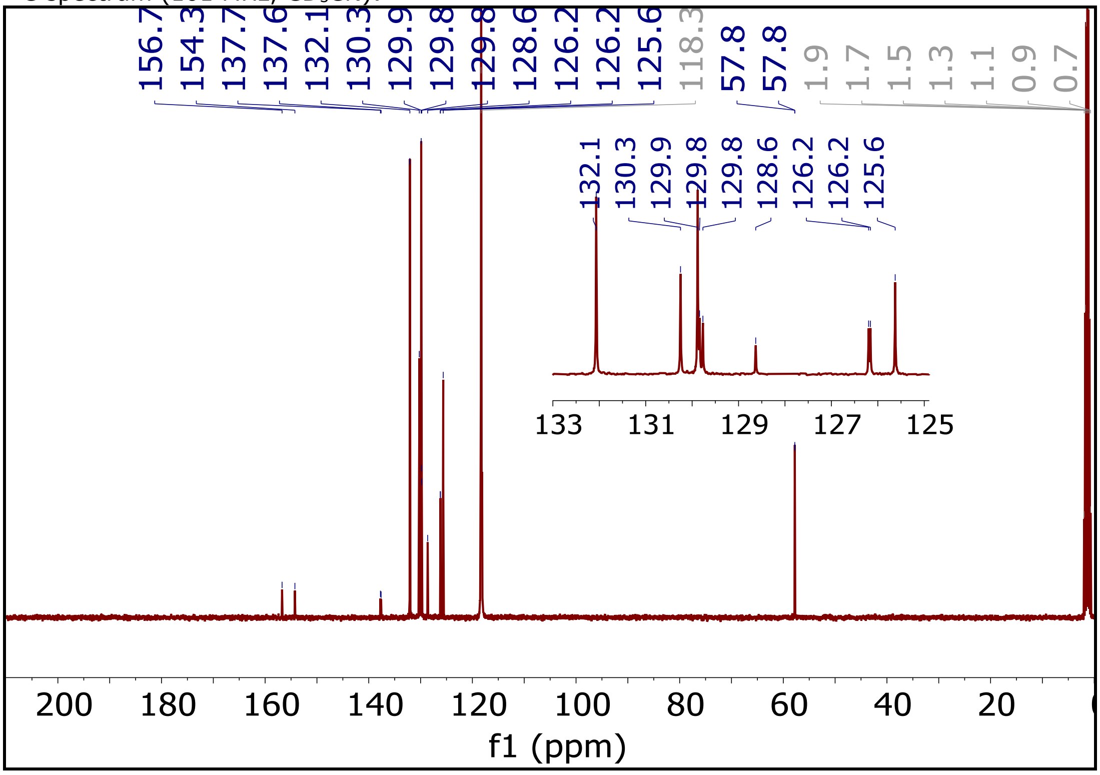

^{19}F spectrum (decoupled) (376 MHz, CD_3CN):

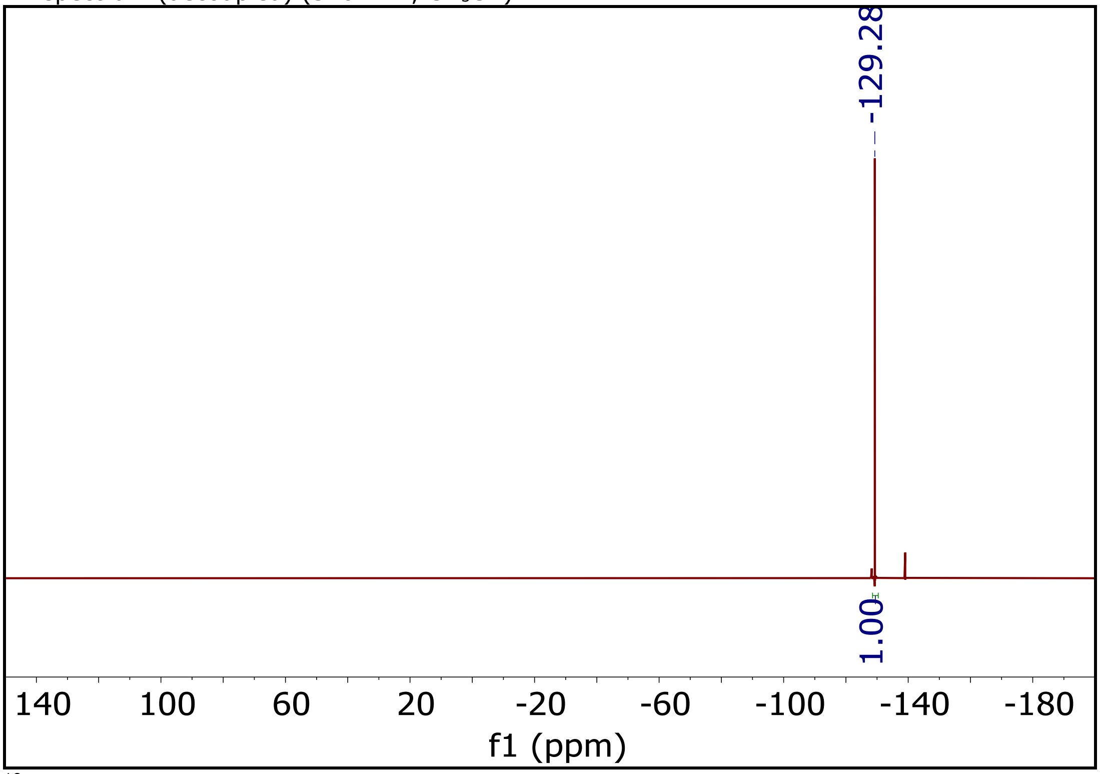

^{19}F spectrum (coupled) (376 MHz, CD_3CN):

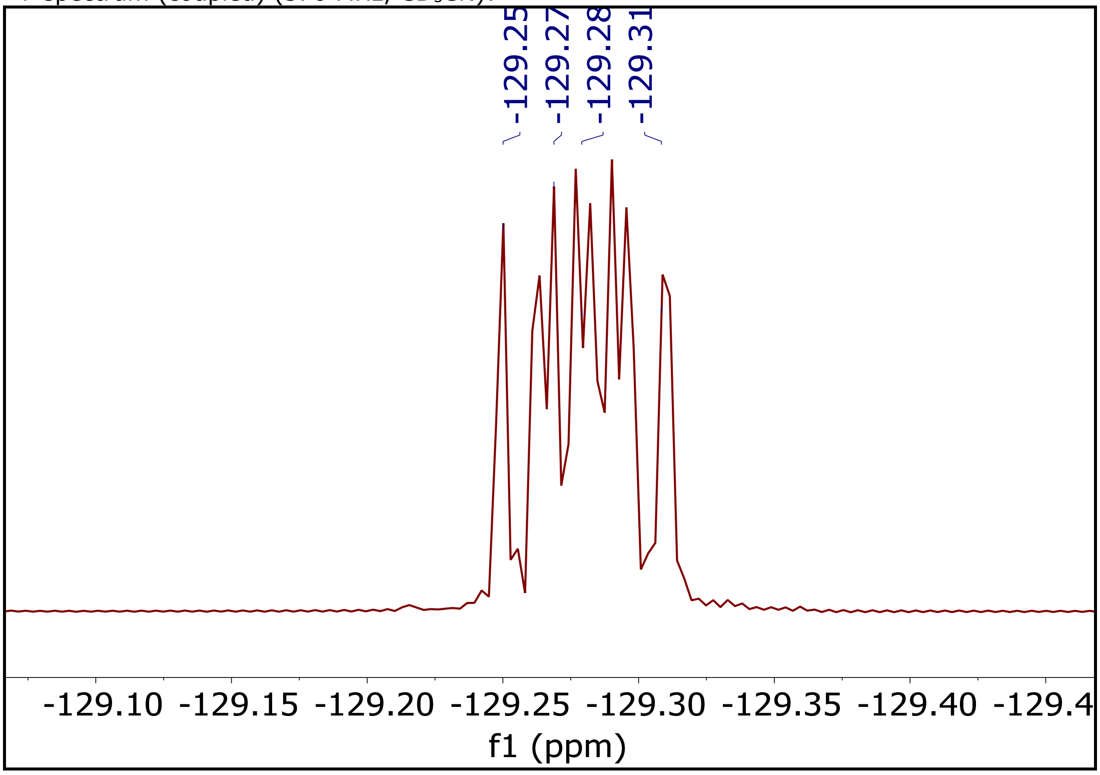

3h-*o*. 2-Bromophenyl benzylsulfonate

^{1}H spectrum (400 MHz, CD_3CN):

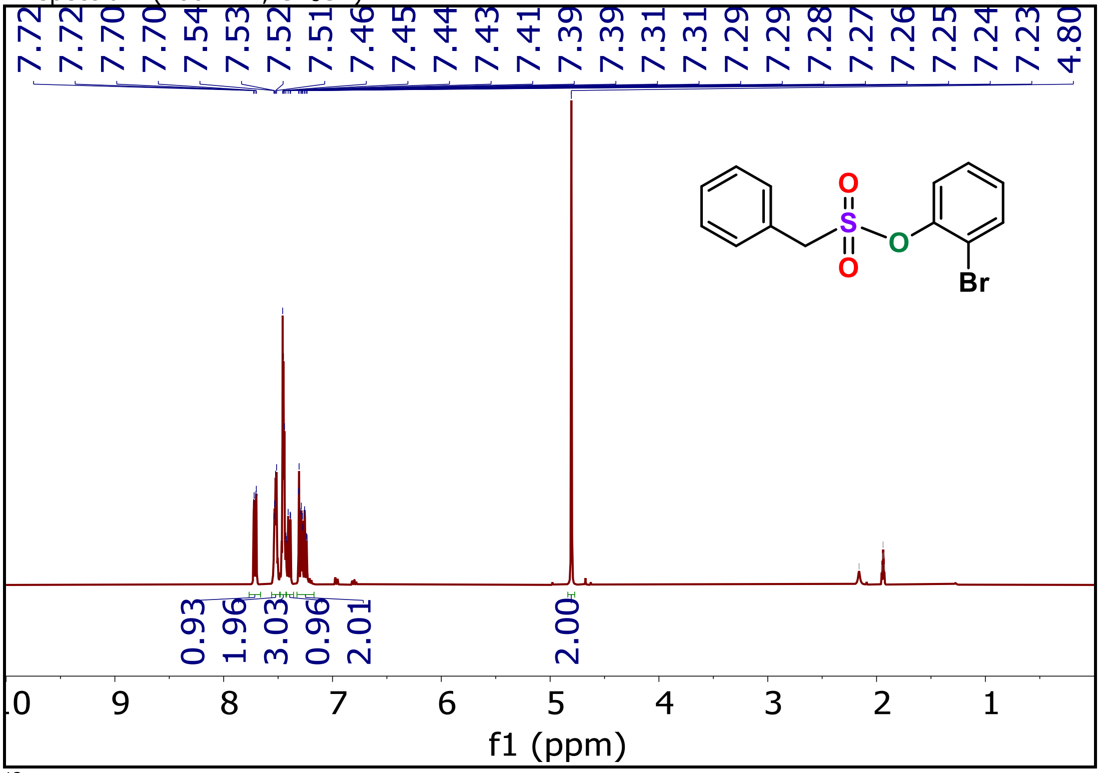

^{13}C spectrum (101 MHz, CD_3CN):

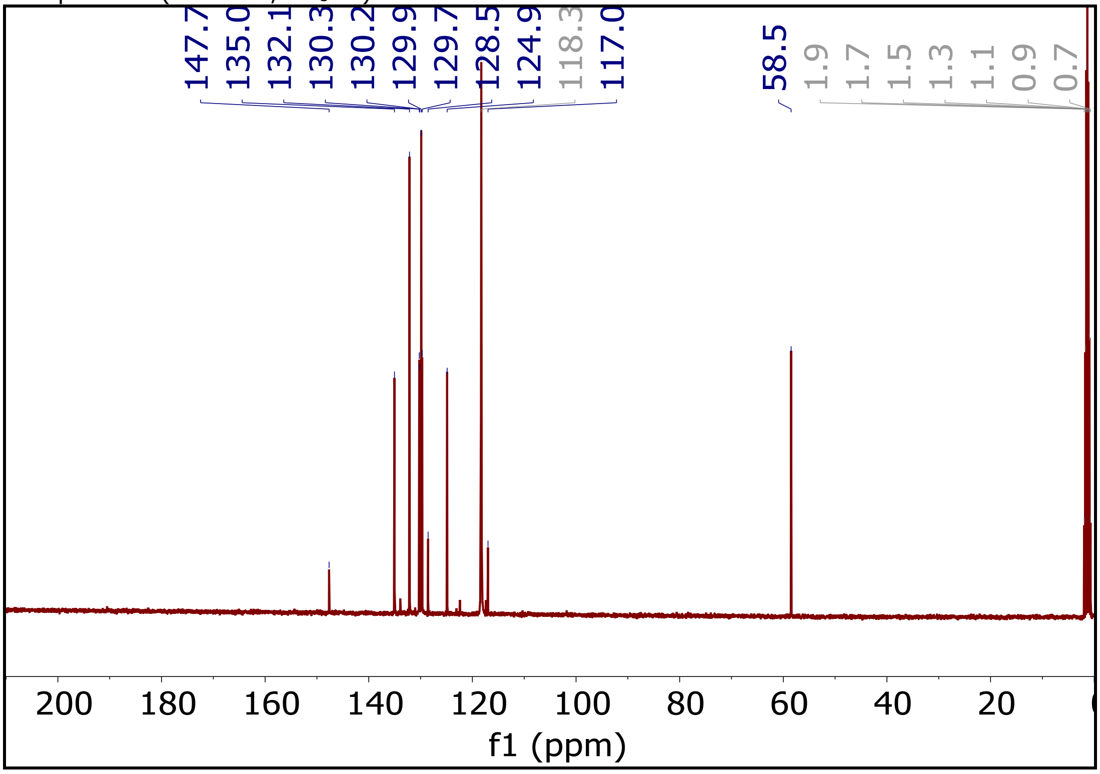

3h-*m*. 3-Bromophenyl benzylsulfonate

^{1}H spectrum (400 MHz, $CDCl_3$):

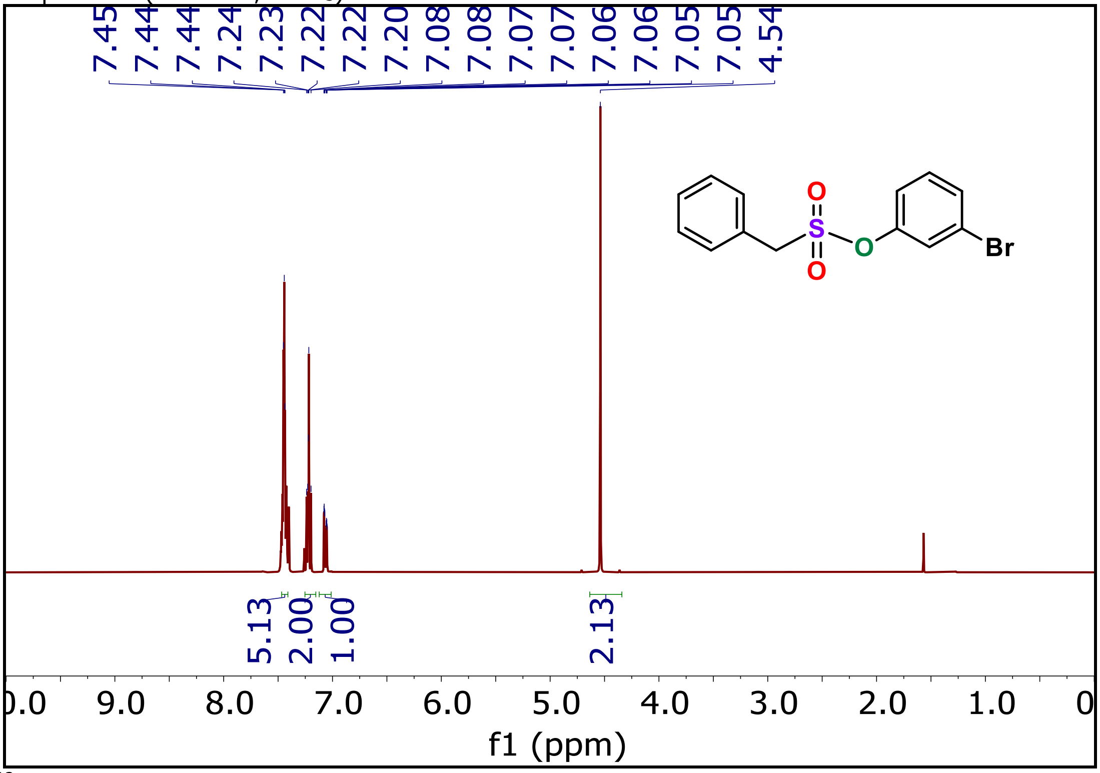

^{13}C spectrum $CDCl_3$ (101 MHz, CD_3CN):

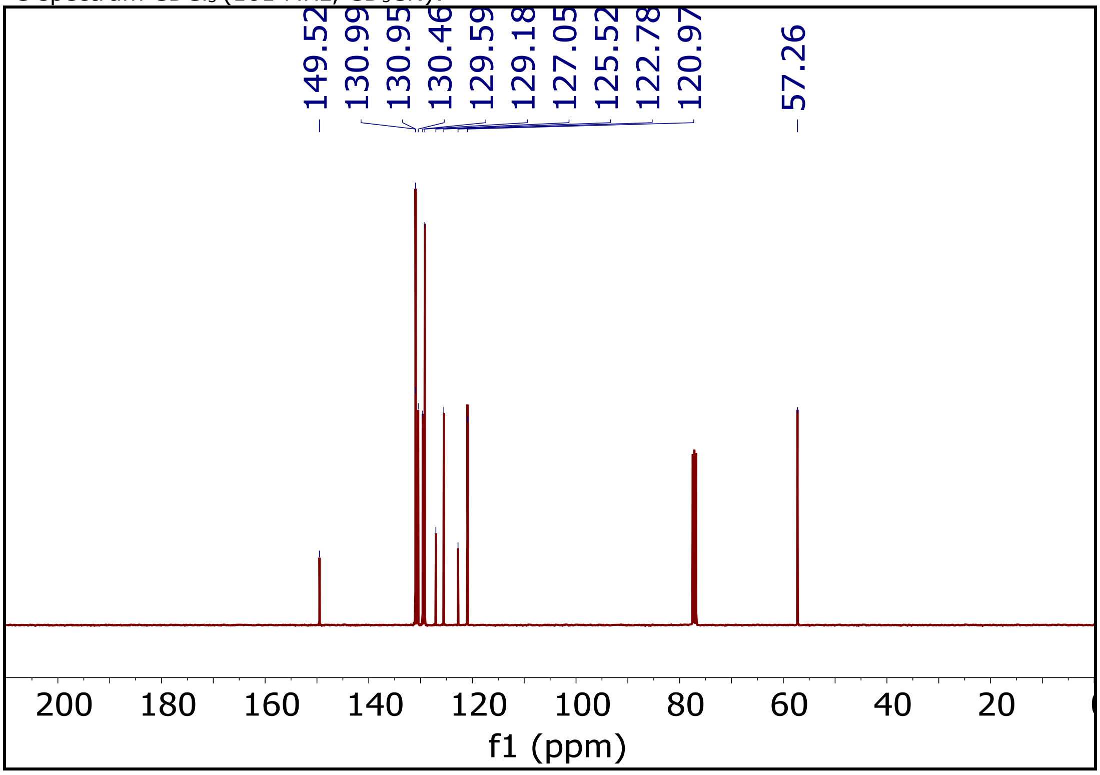

3i. 4-Iodophenyl benzylsulfonate

^{1}H spectrum (400 MHz, CD_3CN):

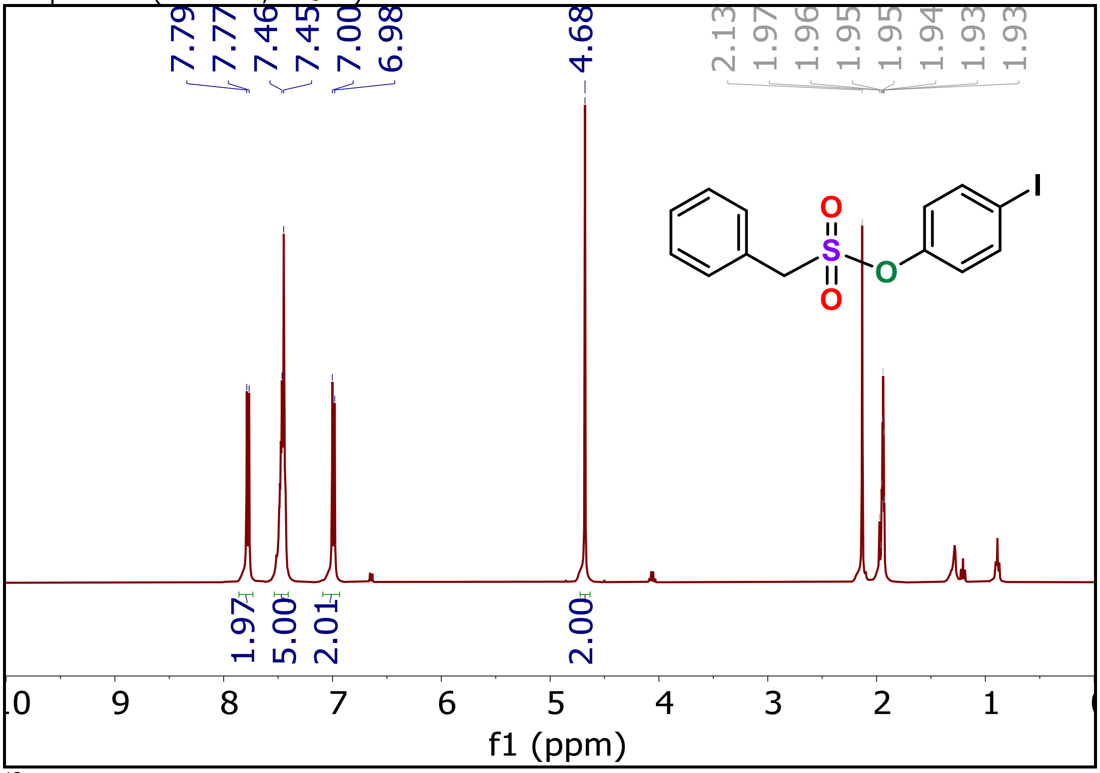

^{13}C spectrum (101 MHz, CD_3CN):

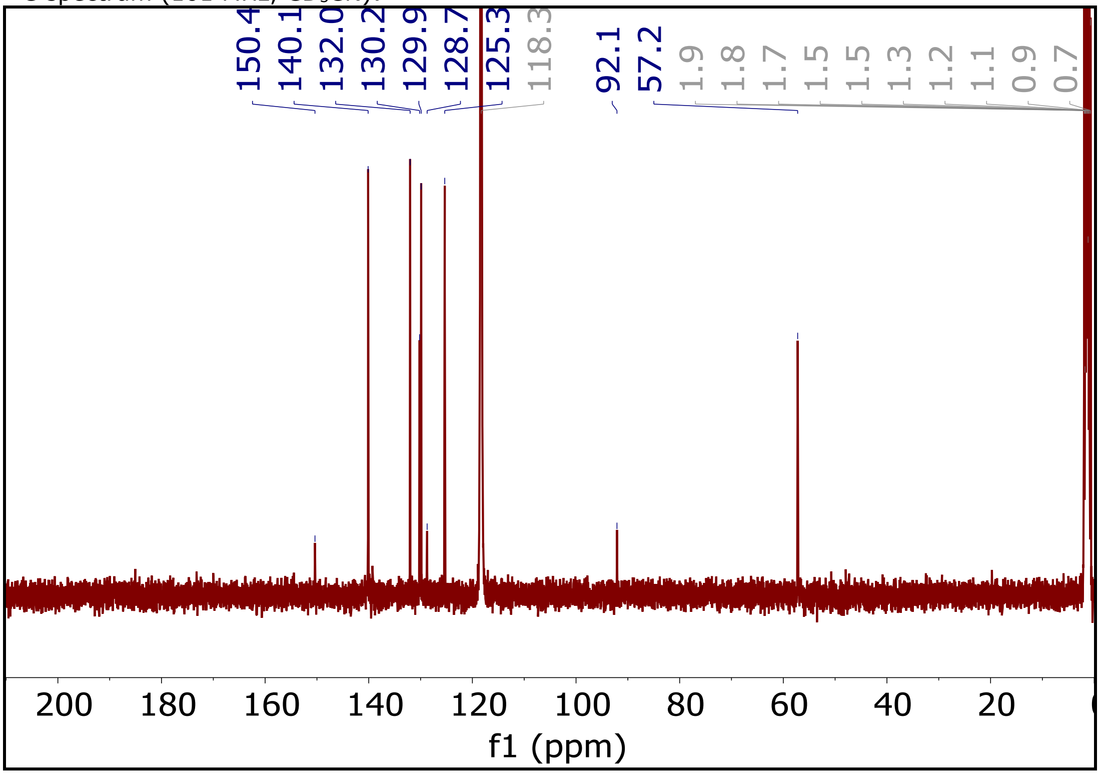

3j. 4-(Trifluoromethoxy)phenyl benzylsulfonate

^{1}H spectrum (400 MHz, CD_3CN):

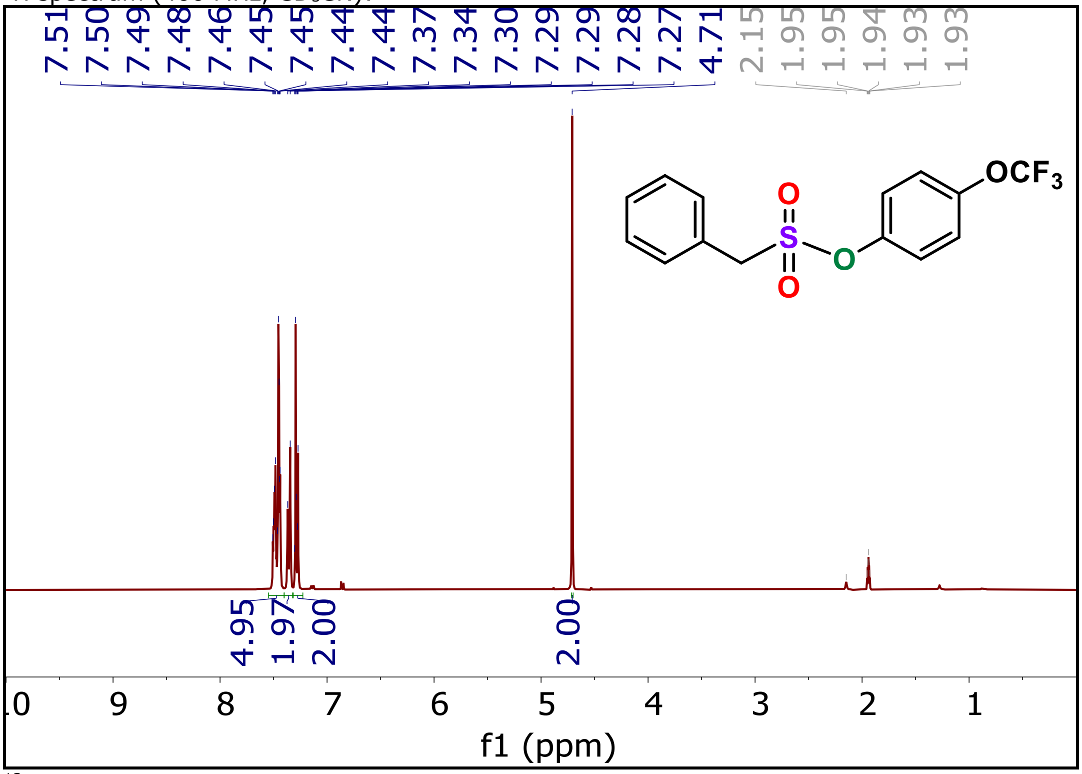

^{13}C spectrum (101 MHz, CD_3CN):

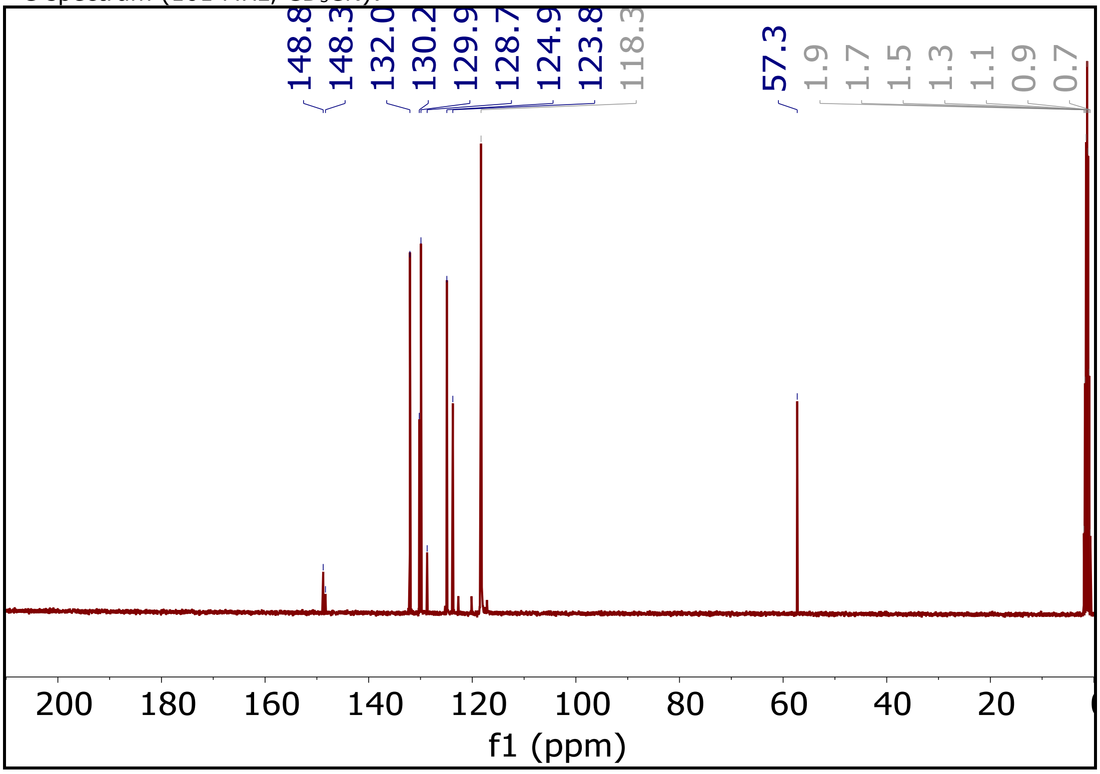

^{19}F spectrum (decoupled) (376 MHz, CD_3CN):

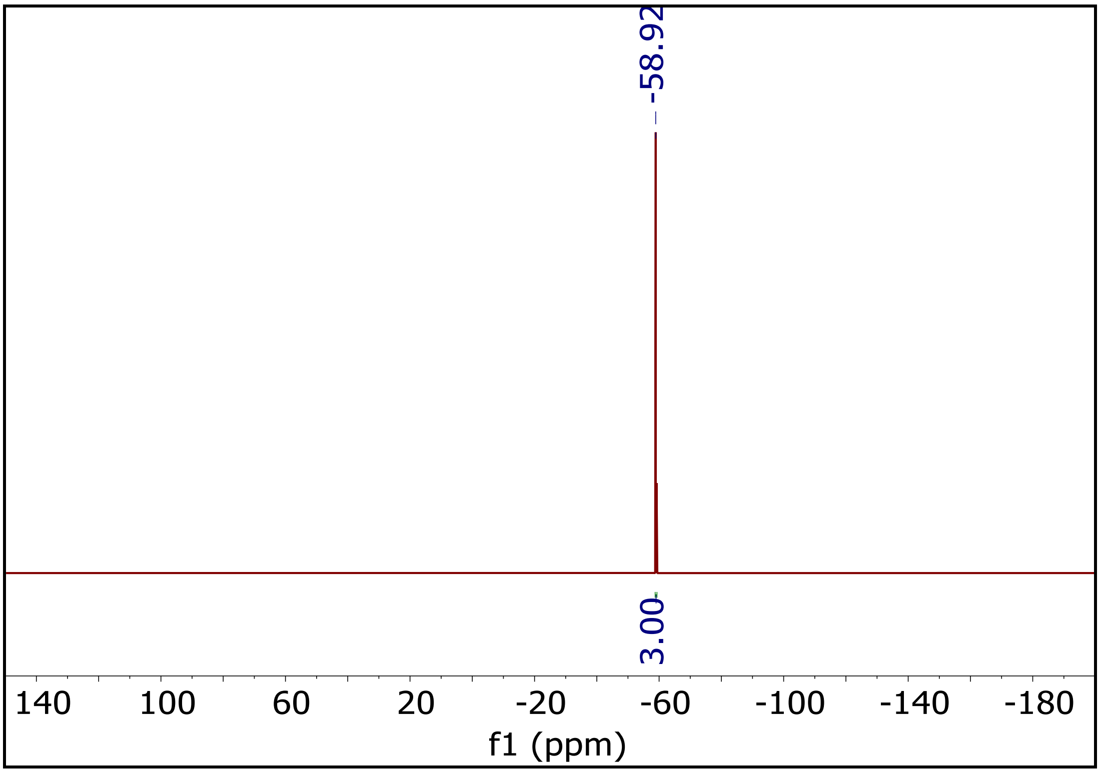

3k-*p*. 4-Cyanophenyl benzylsulfonate

^{1}H spectrum (400 MHz, CD_3CN):

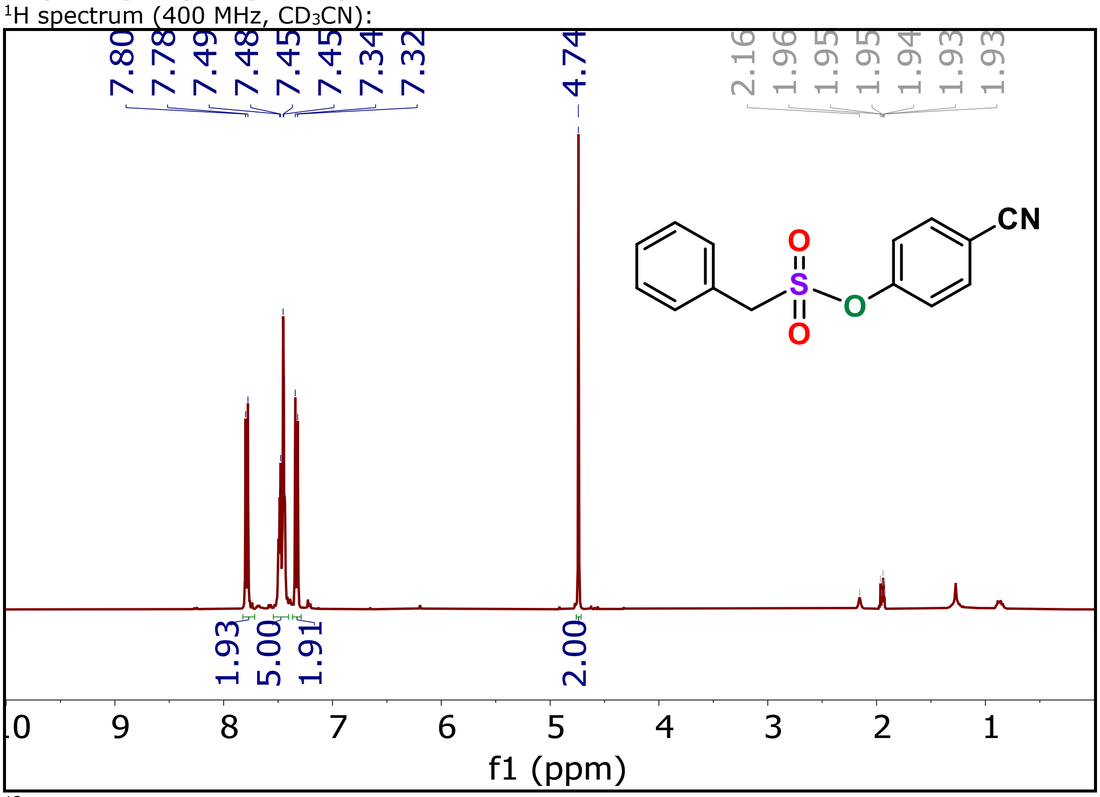

^{13}C spectrum (101 MHz, CD_3CN):

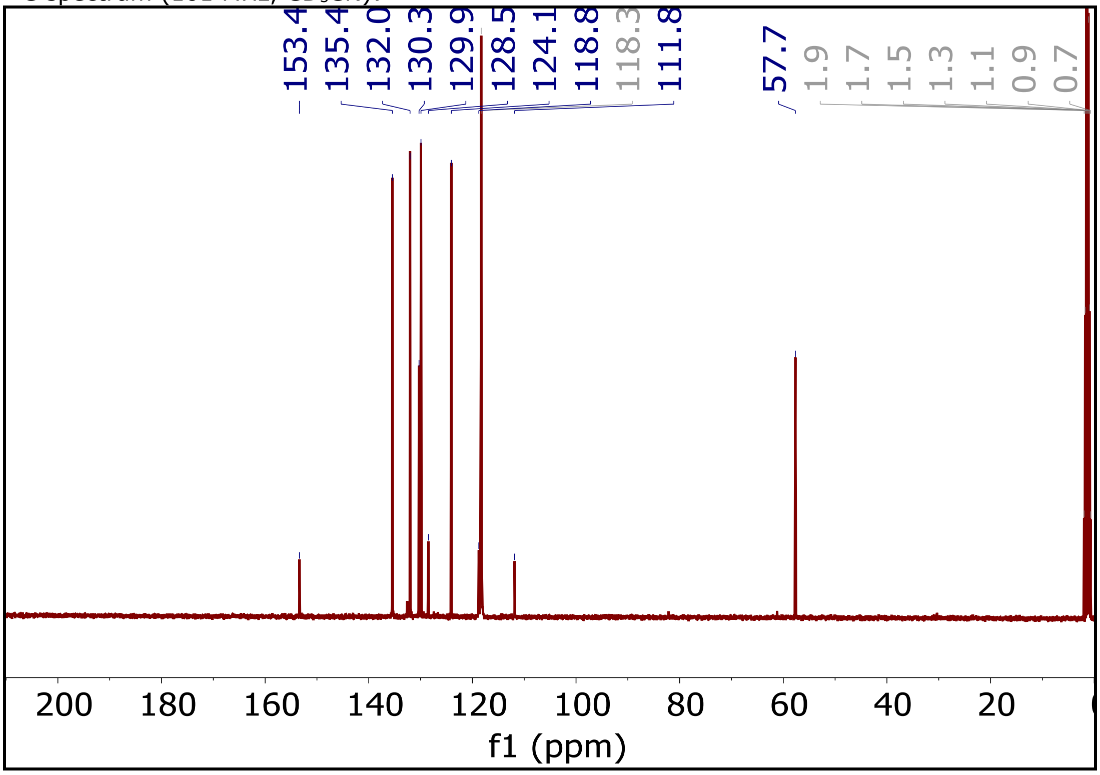

3l. 3,4,5-Trifluorophenyl benzylsulfonate

^{1}H spectrum (400 MHz, CD_3CN):

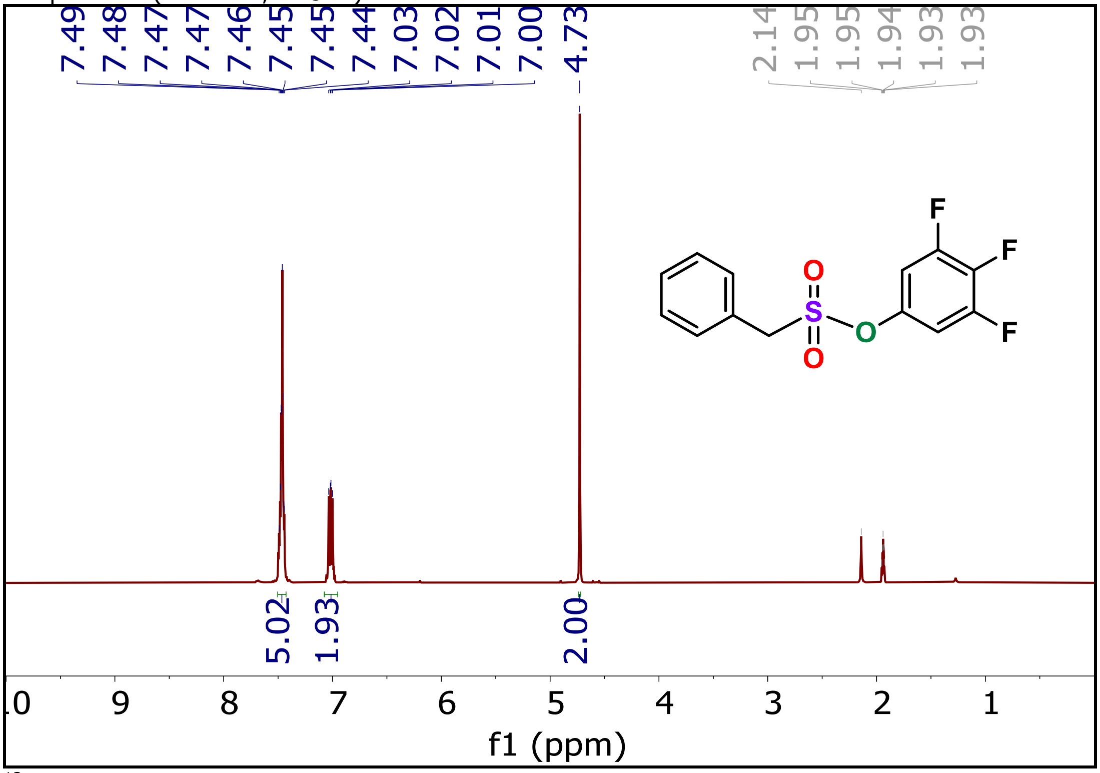

^{13}C spectrum (101 MHz, CD_3CN):

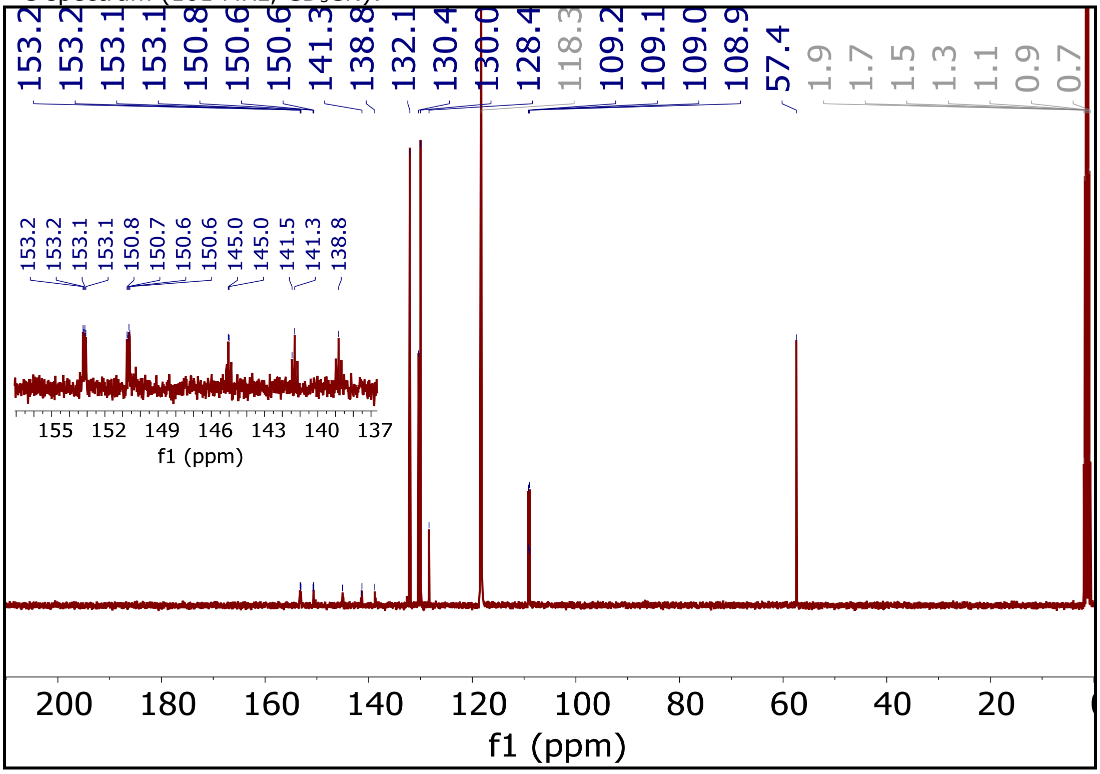

^{19}F spectrum (decoupled) (376 MHz, CD_3CN):

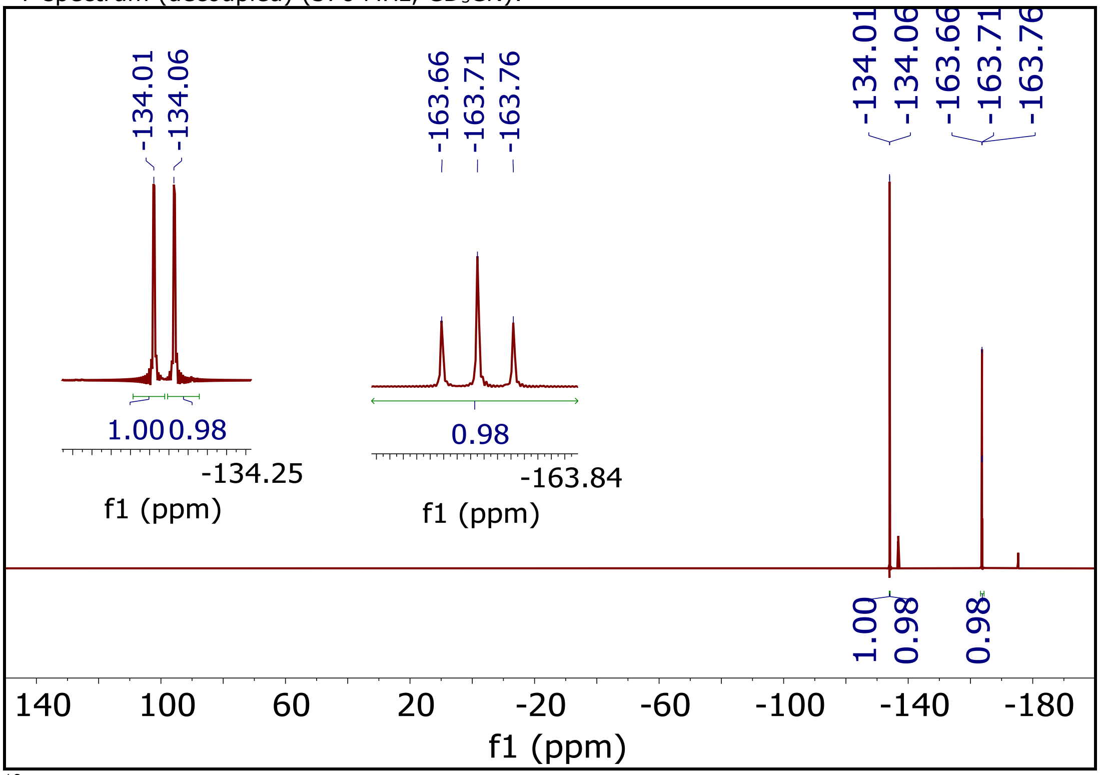

^{19}F spectrum (coupled) (376 MHz, CD_3CN):

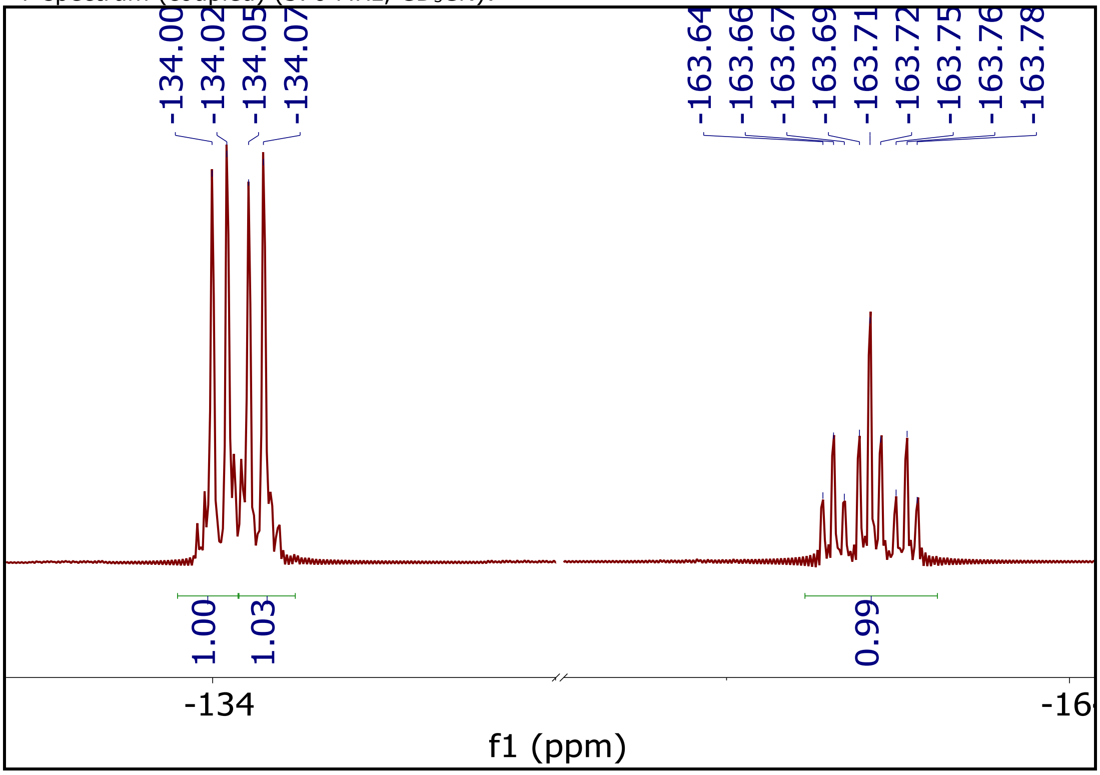

3m. 4-(Hydroxymethyl)phenyl benzylsulfonate

^{1}H spectrum (400 MHz, DMSO-d6):

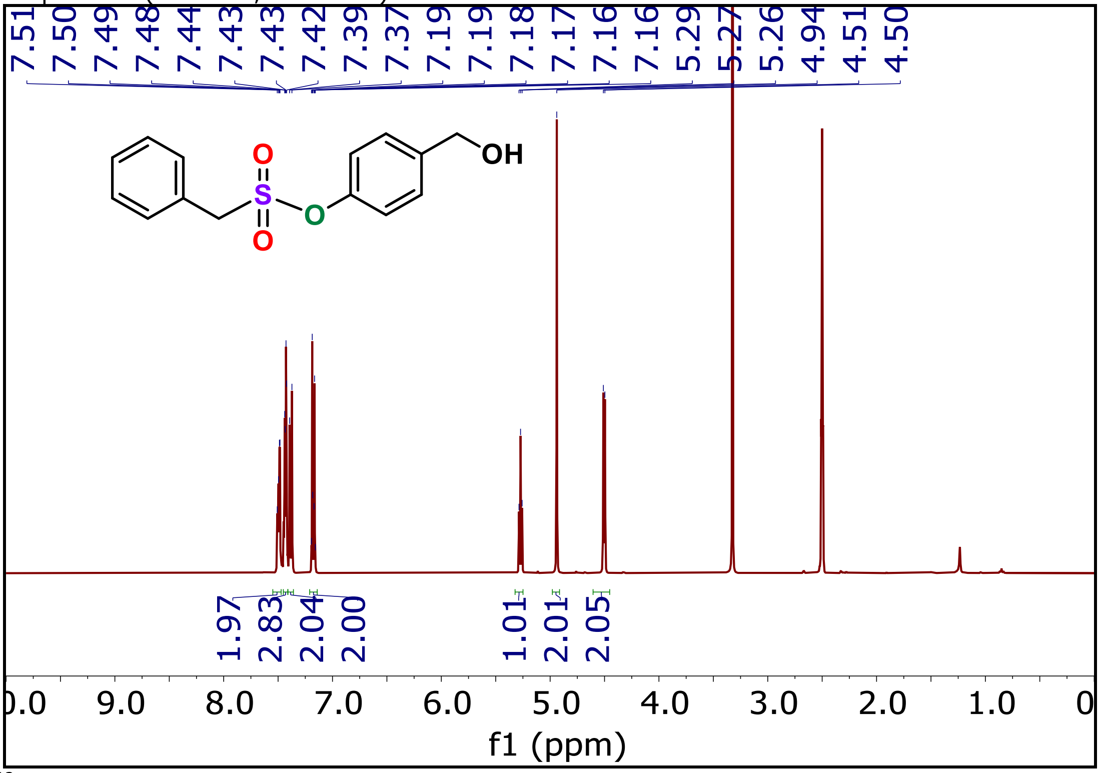

^{13}C spectrum (101 MHz, DMSO-d6):

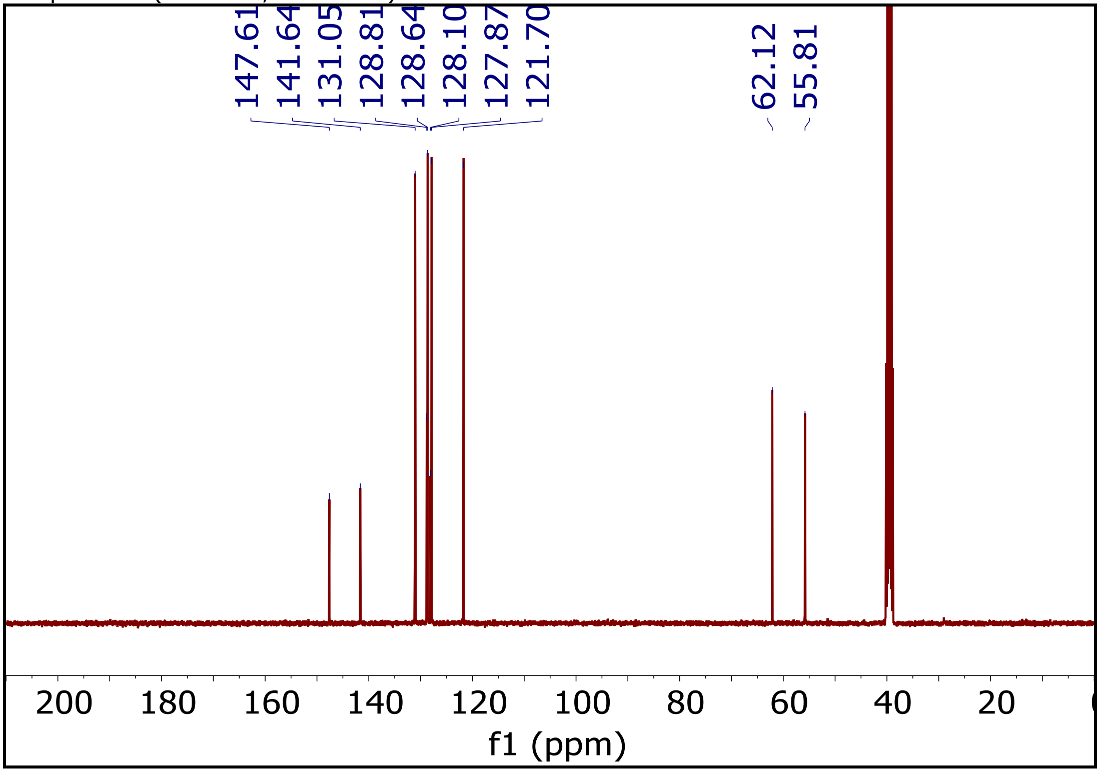

3o-*o*. 2-Tert-butylphenyl benzylsulfonate

^{1}H spectrum (400 MHz, CD_3CN):

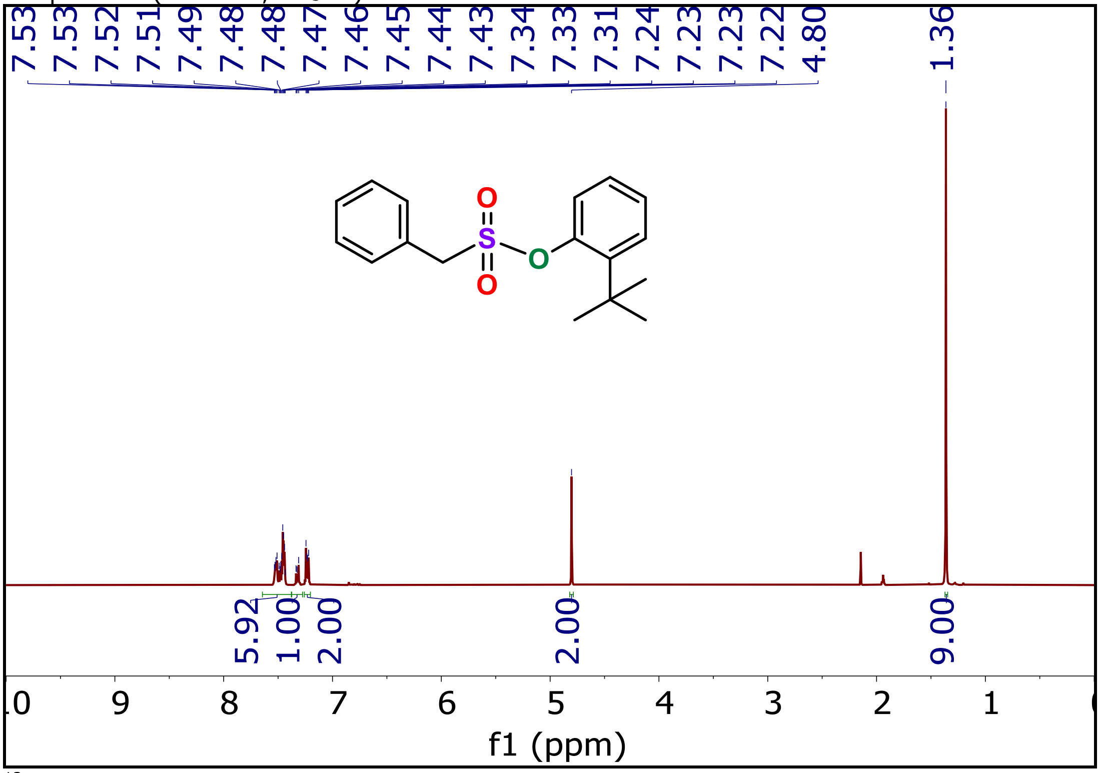

^{13}C spectrum (101 MHz, CD_3CN):

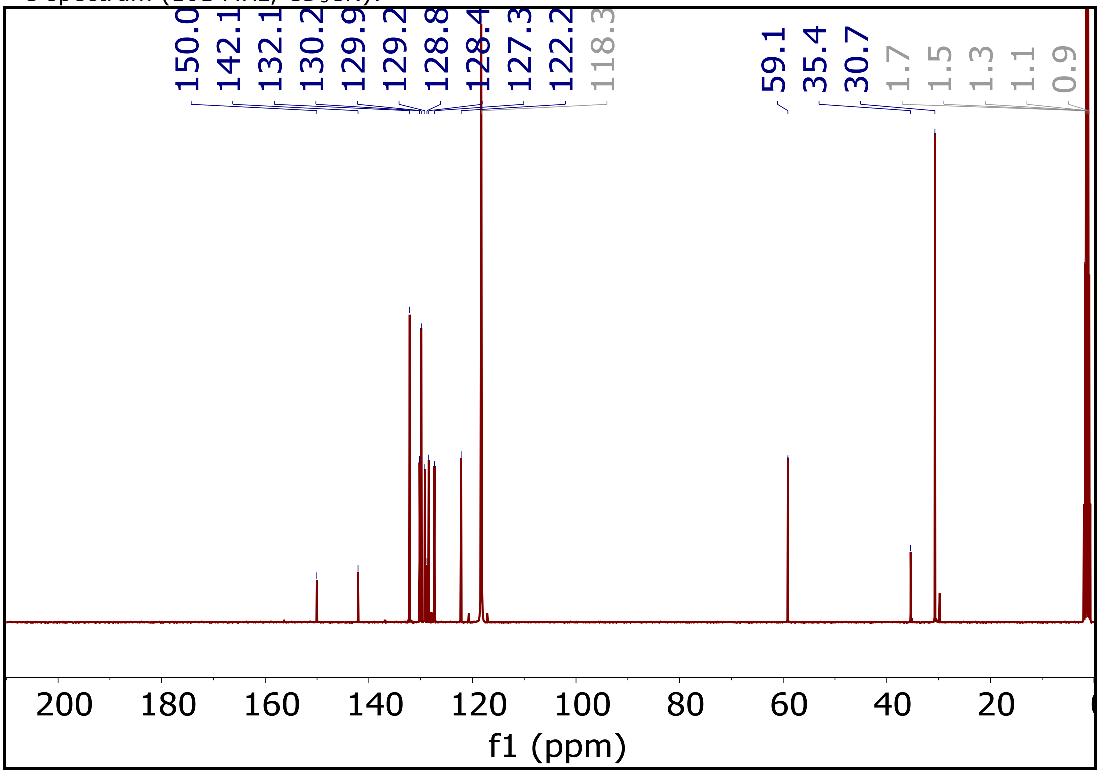

3o-*p*. 4-Tert-butylphenyl benzylsulfonate

^{1}H spectrum (400 MHz, CD_3CN):

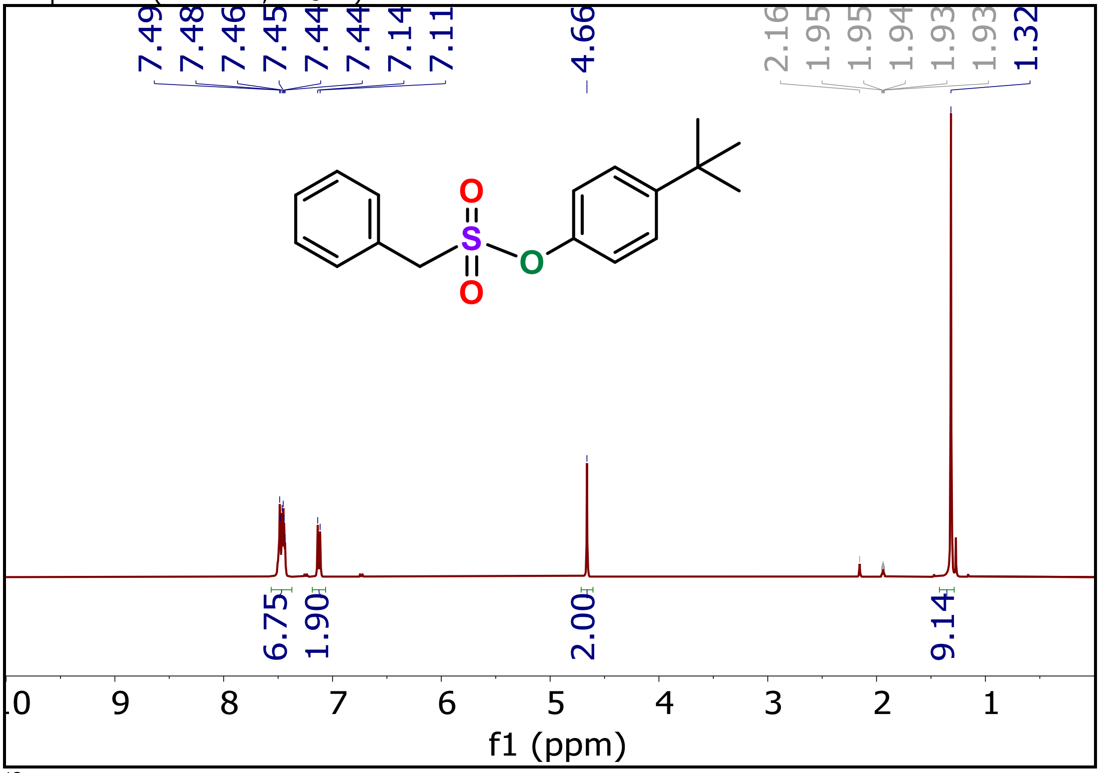

^{13}C spectrum (101 MHz, CD_3CN):

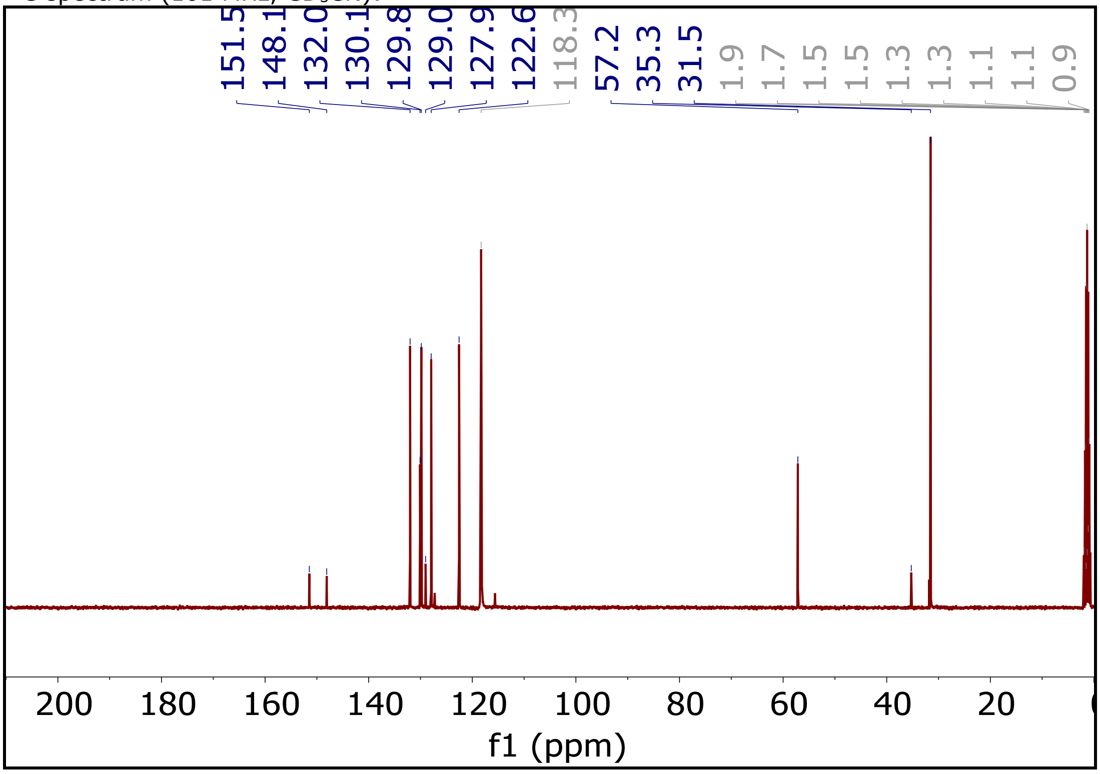

3p. Pyridin-3-yl benzylsulfonate

^{1}H spectrum (400 MHz, CD_3CN):

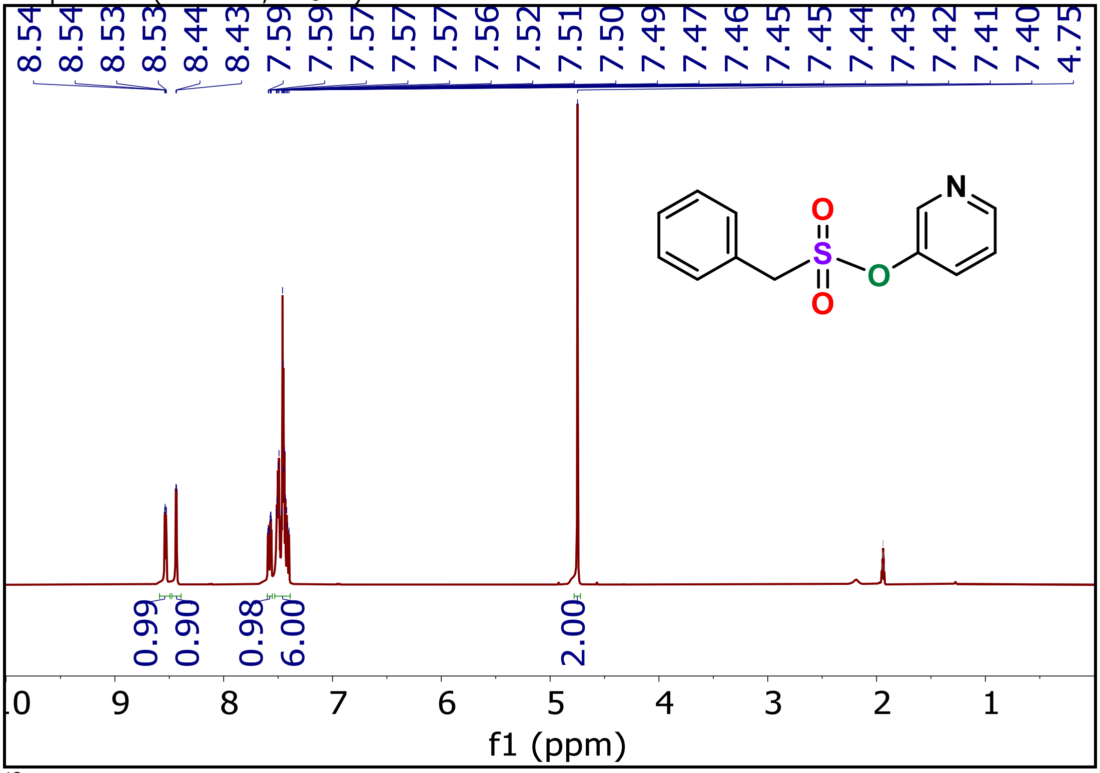

^{13}C spectrum (101 MHz, CD_3CN):

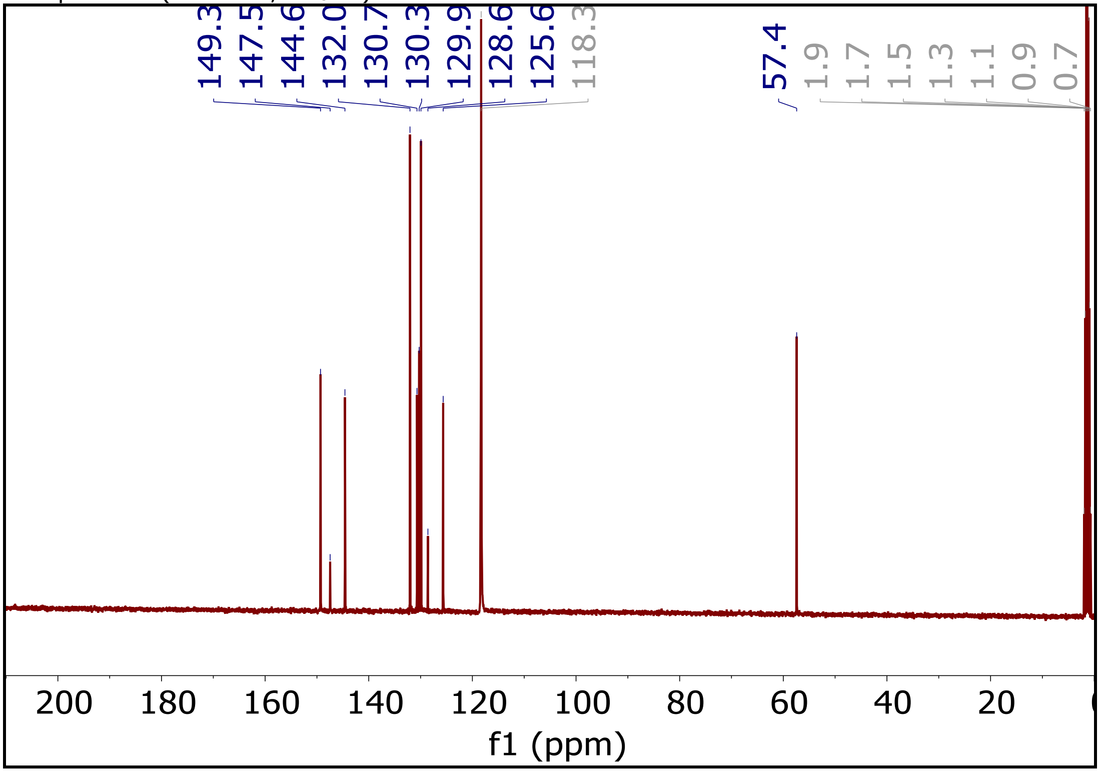

3q. 4-(Trifluoromethyl)phenyl benzylsulfonate

^{1}H spectrum (400 MHz, CD_3CN):

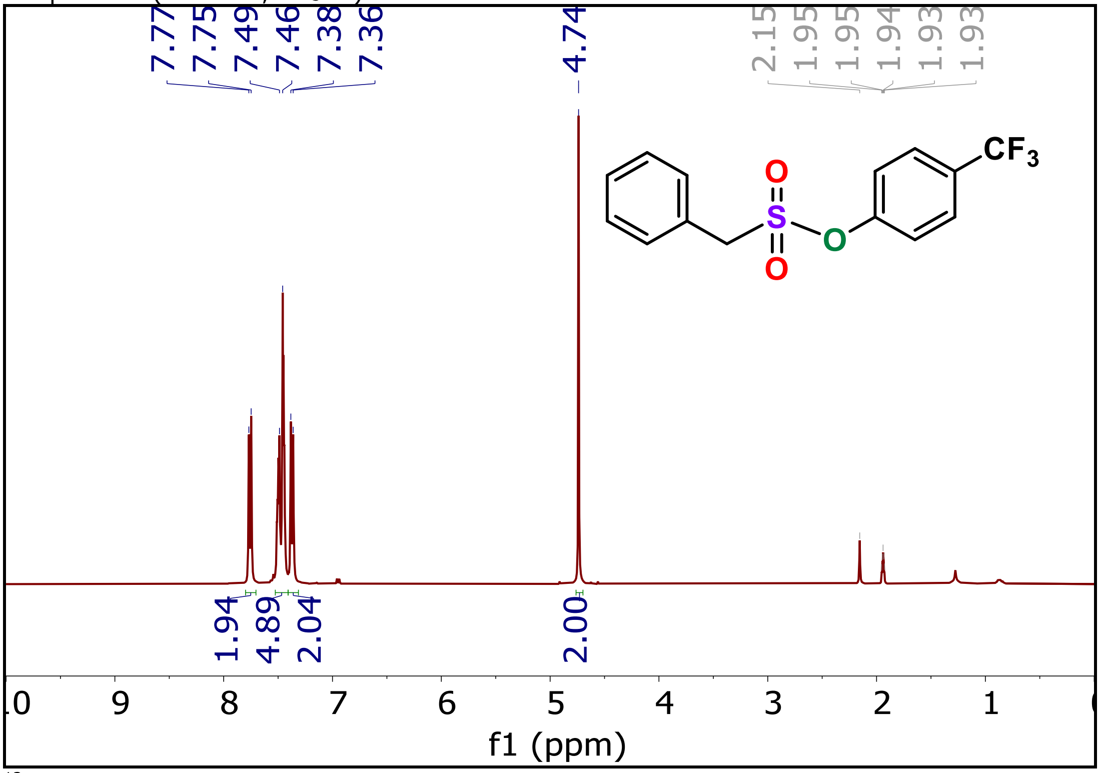

^{13}C spectrum (101 MHz, CD_3CN):

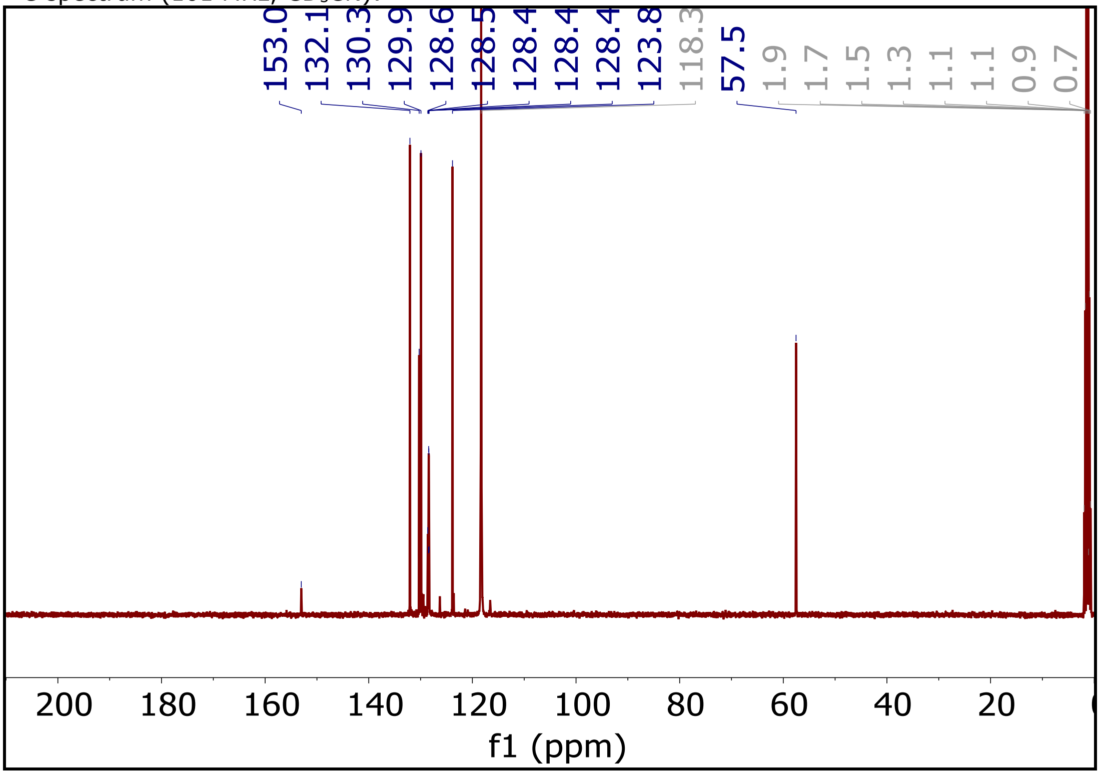

^{19}F spectrum (decoupled) (376 MHz, CD_3CN):

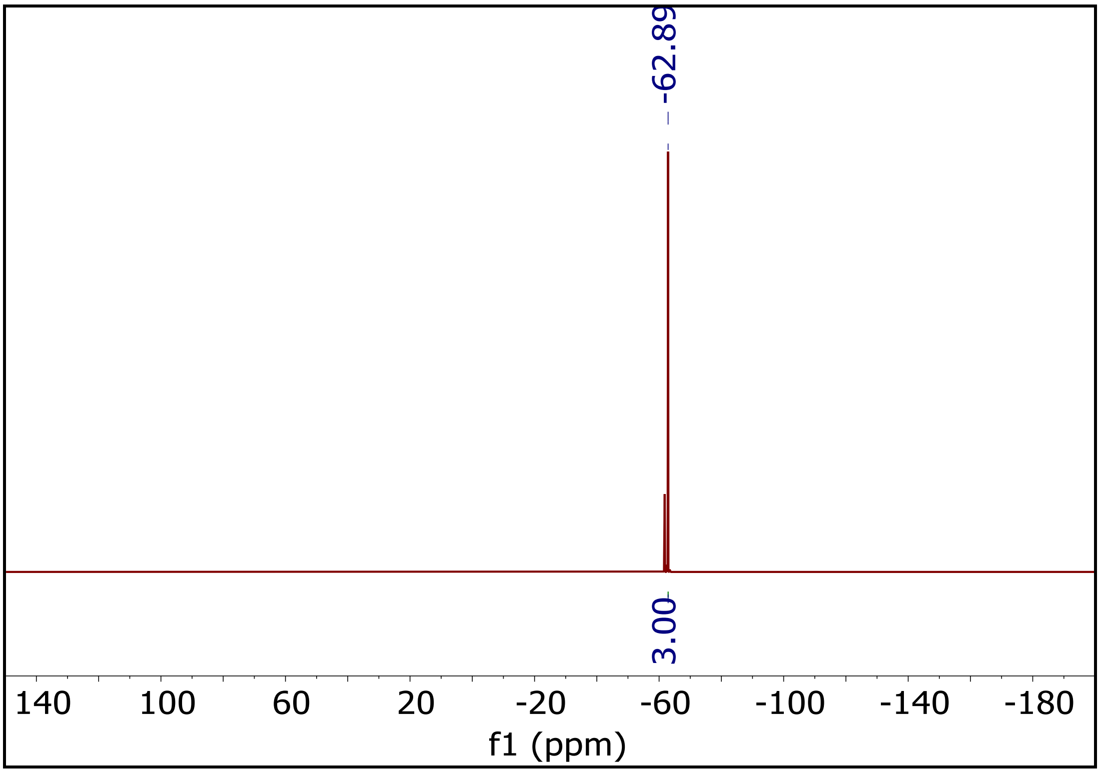

3s. 4-Acetamidophenyl benzylsulfonate

^{1}H spectrum (400 MHz, CD_3CN):

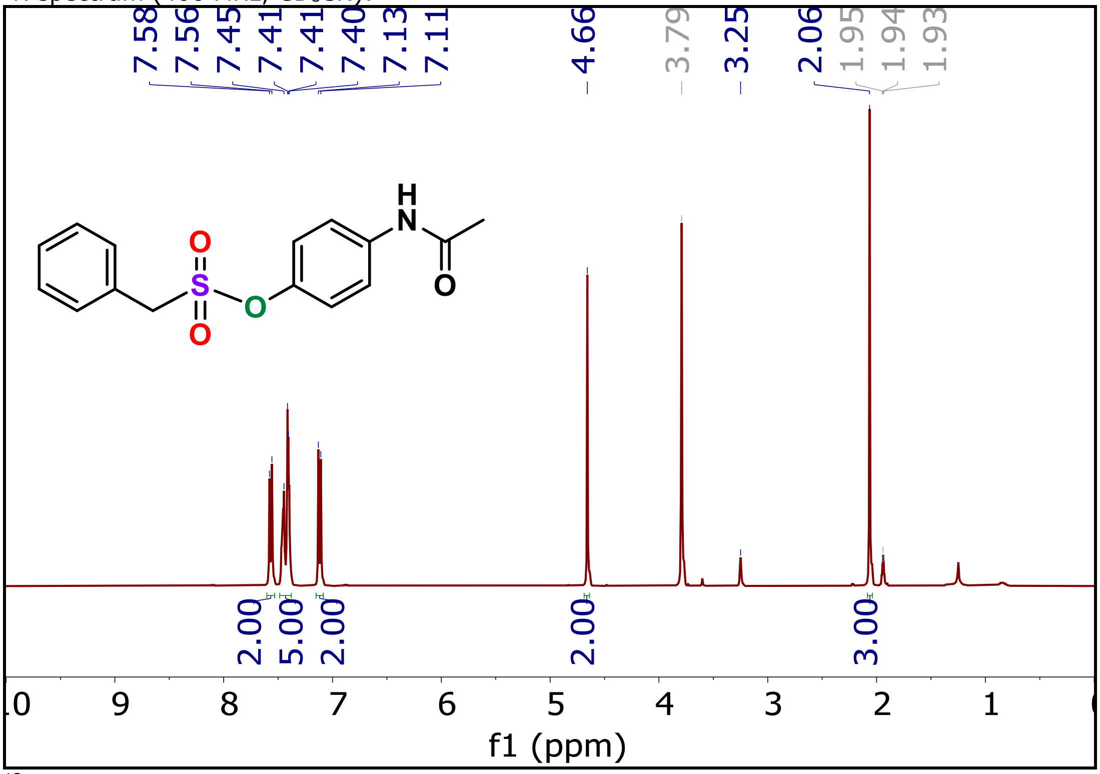

^{13}C spectrum (101 MHz, CD_3CN):

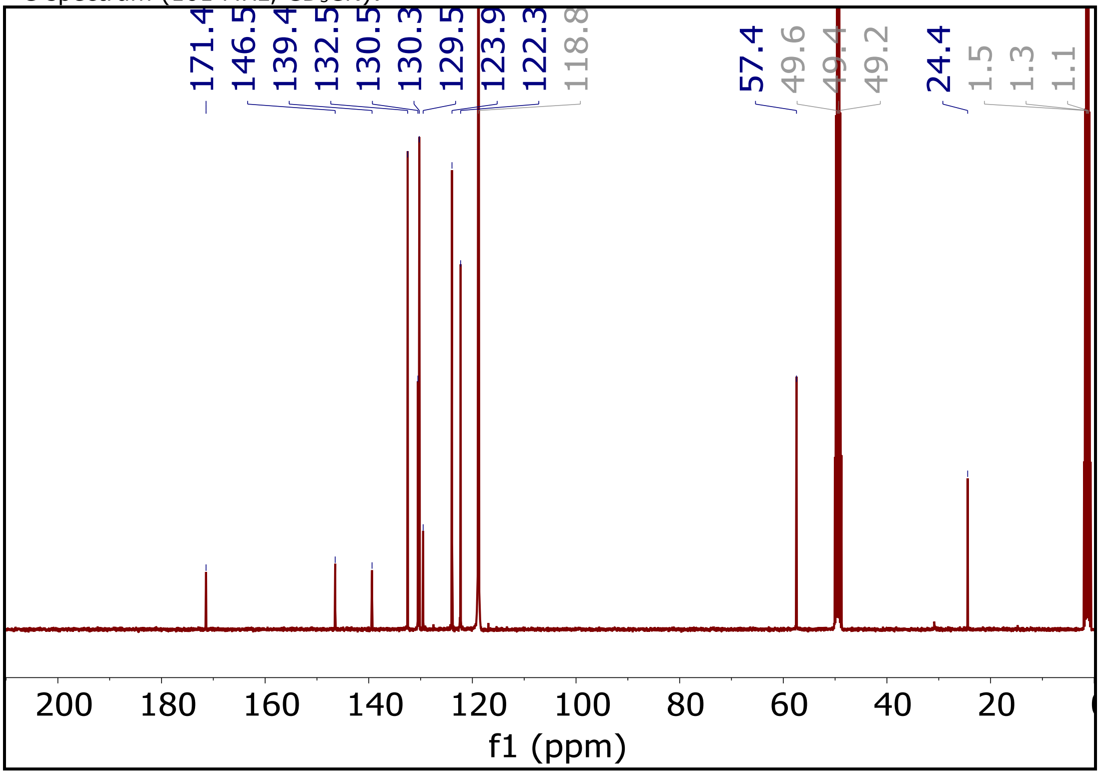

3u. 3,5-Bis(trifluoromethyl)phenyl benzylsulfonate

^{1}H spectrum (400 MHz, CD_3CN):

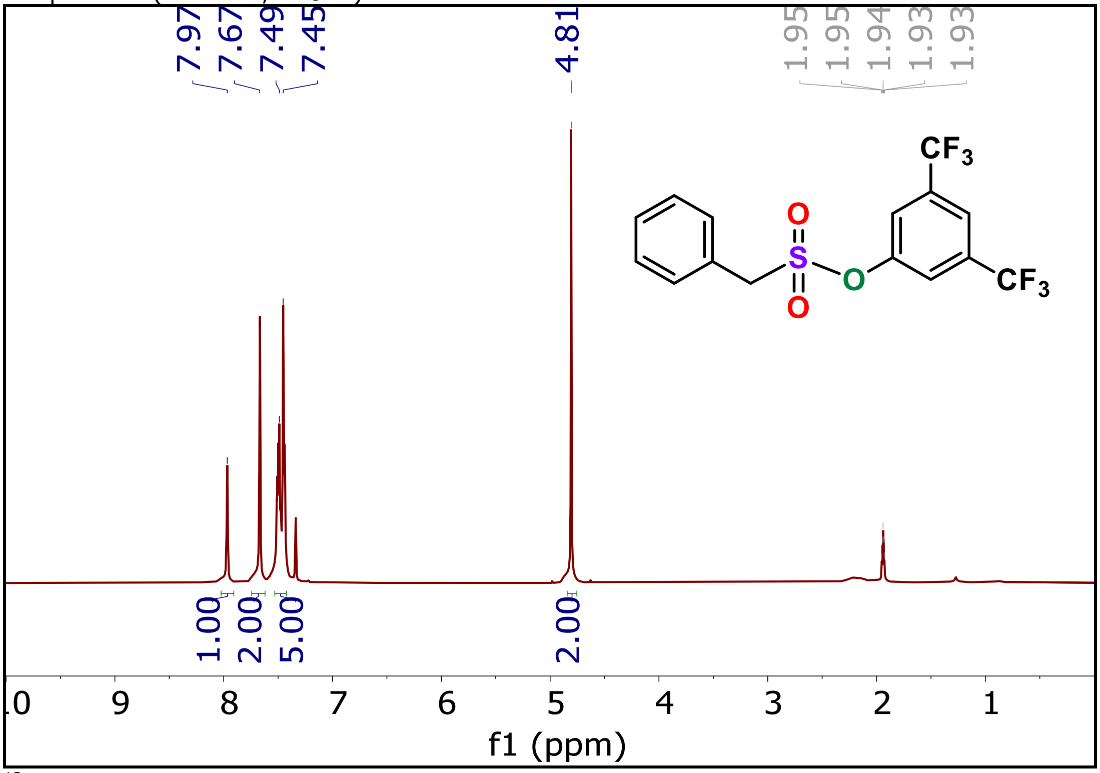

^{13}C spectrum (101 MHz, CD_3CN):

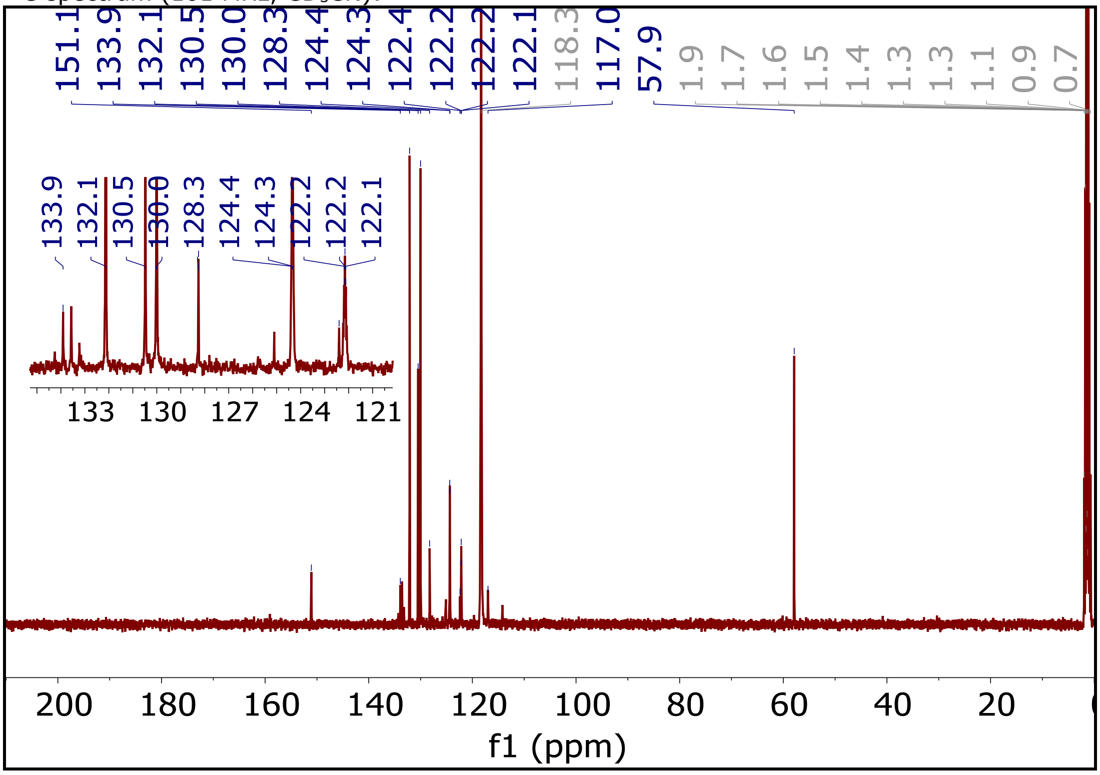

^{19}F spectrum (decoupled) (376 MHz, CD_3CN):

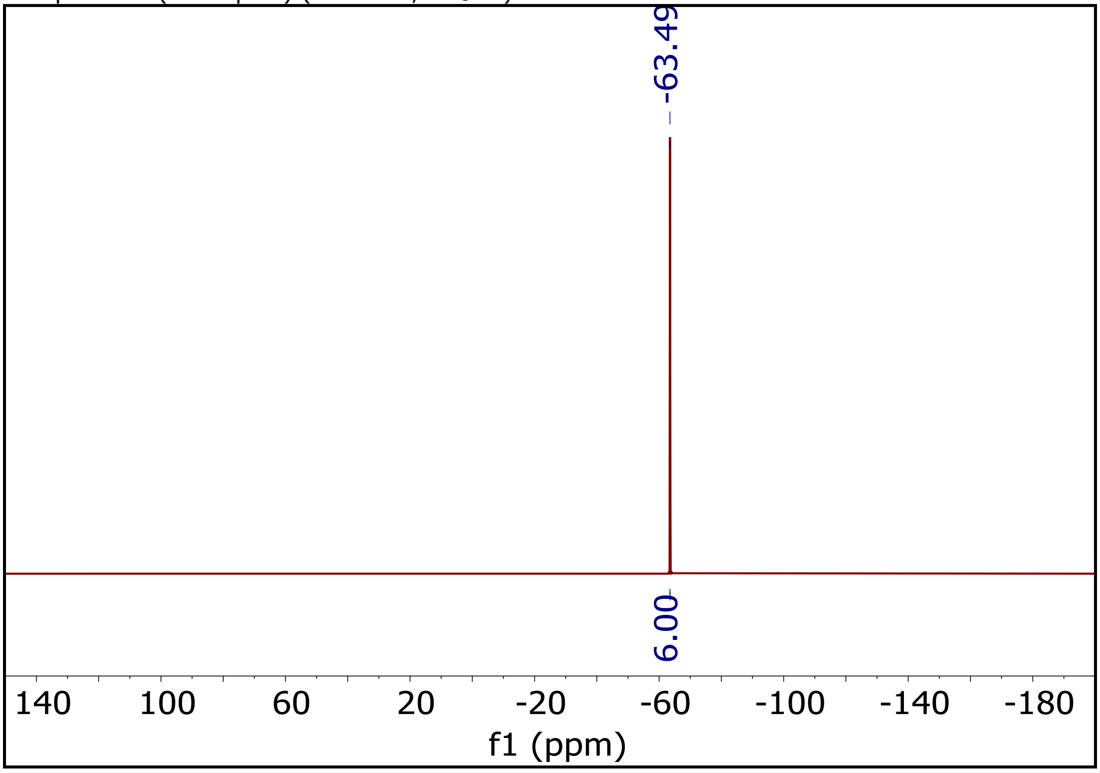

3w. Methyl 4-((benzylsulfonyl)oxy)benzoate

^{1}H spectrum (400 MHz, CD_3CN):

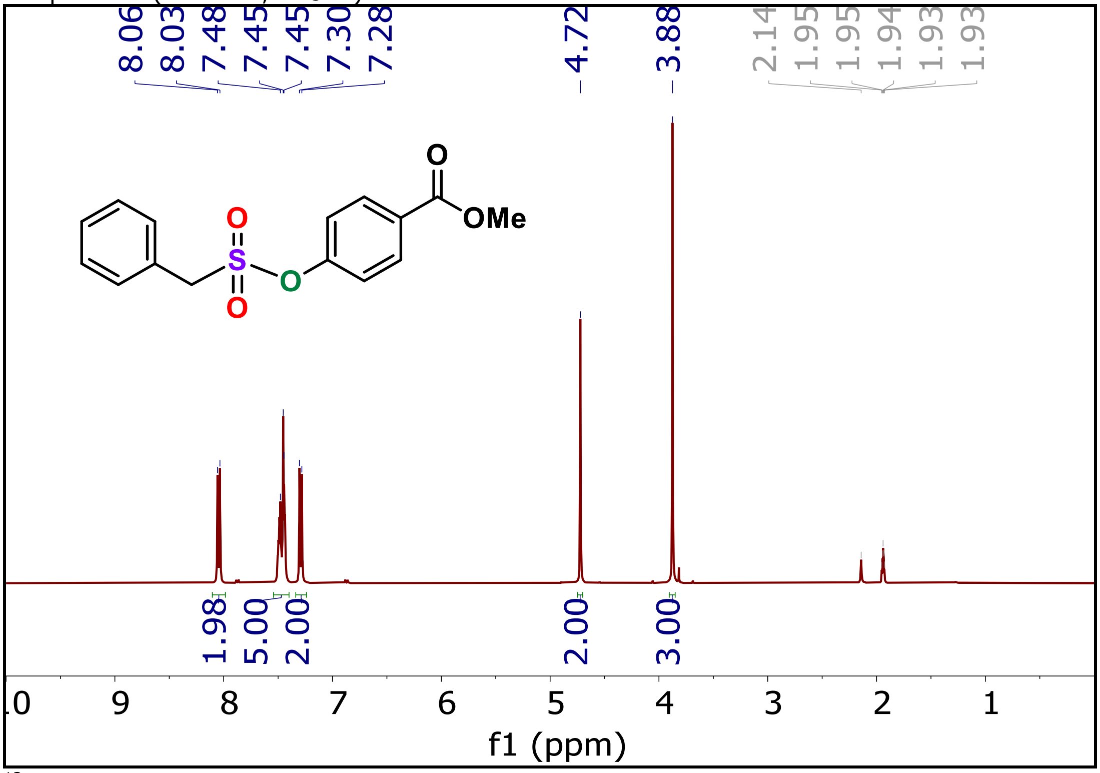

^{13}C spectrum (101 MHz, CD_3CN):

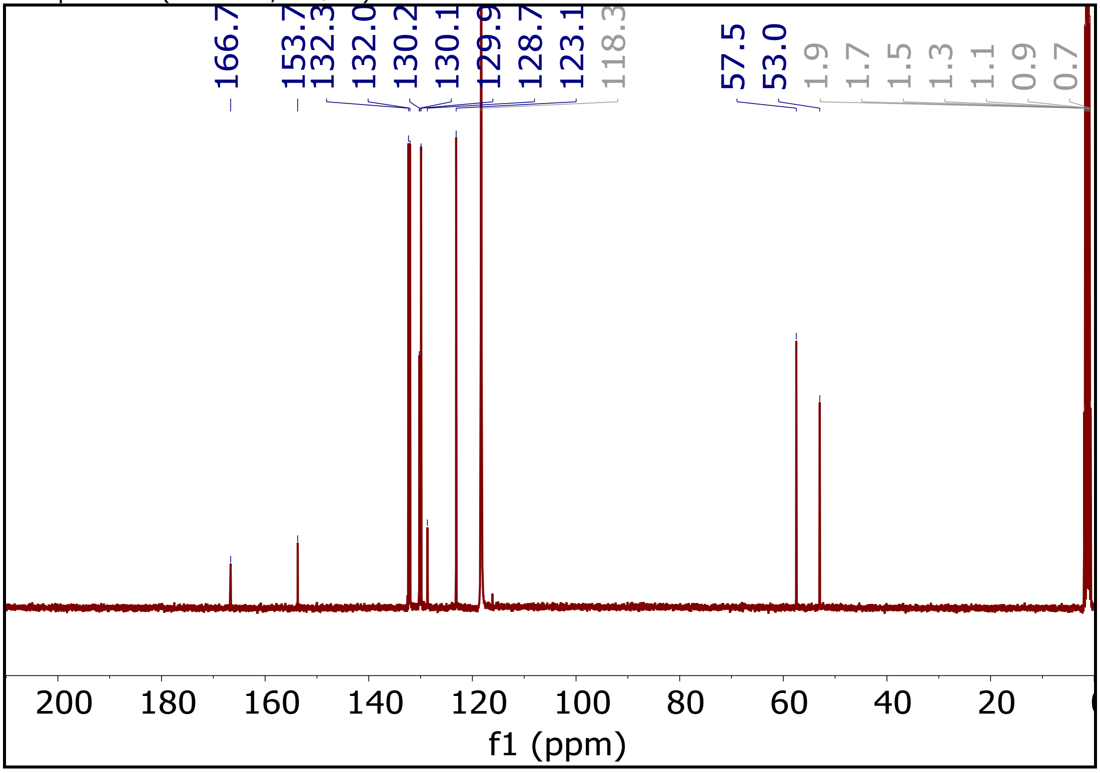

3y: 4-carbamoylphenyl phenylmethanesulfonate

^{1}H spectrum (400 MHz, CD_3CN):

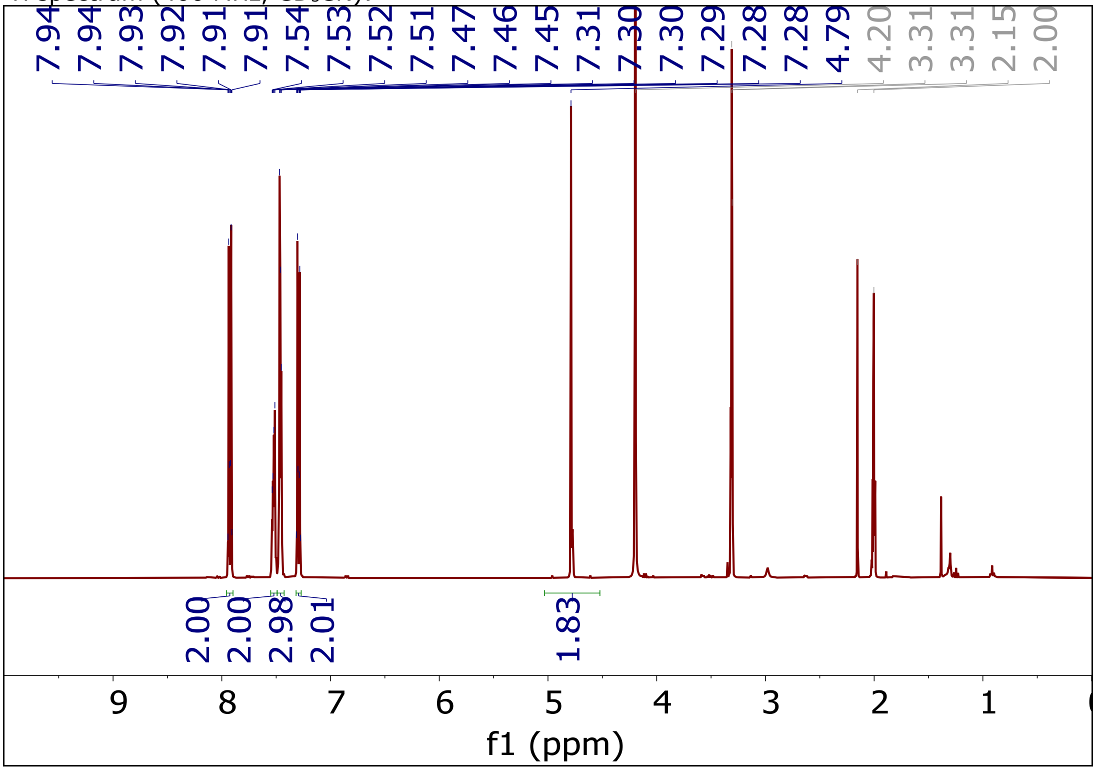

^{13}C spectrum (101 MHz, CD_3CN):

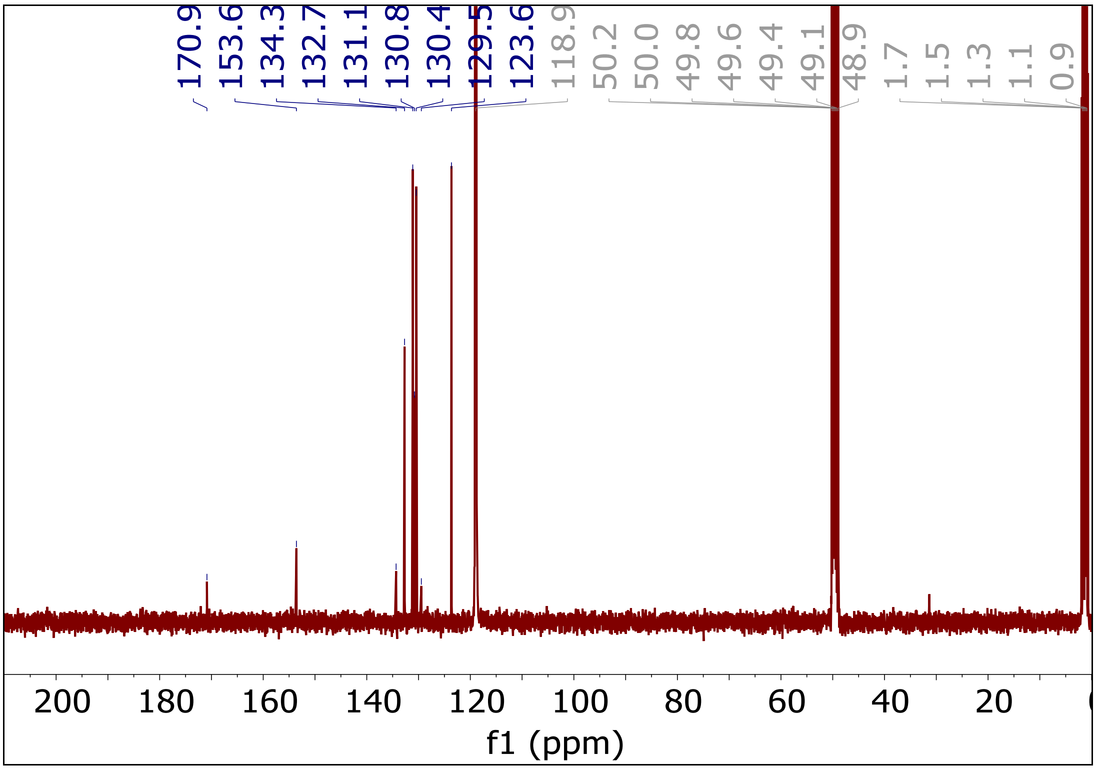

3aa. 4-((4-Isopropoxyphenyl)sulfonyl)phenyl benzylsulfonate

^{1}H spectrum (400 MHz, CD_3CN):

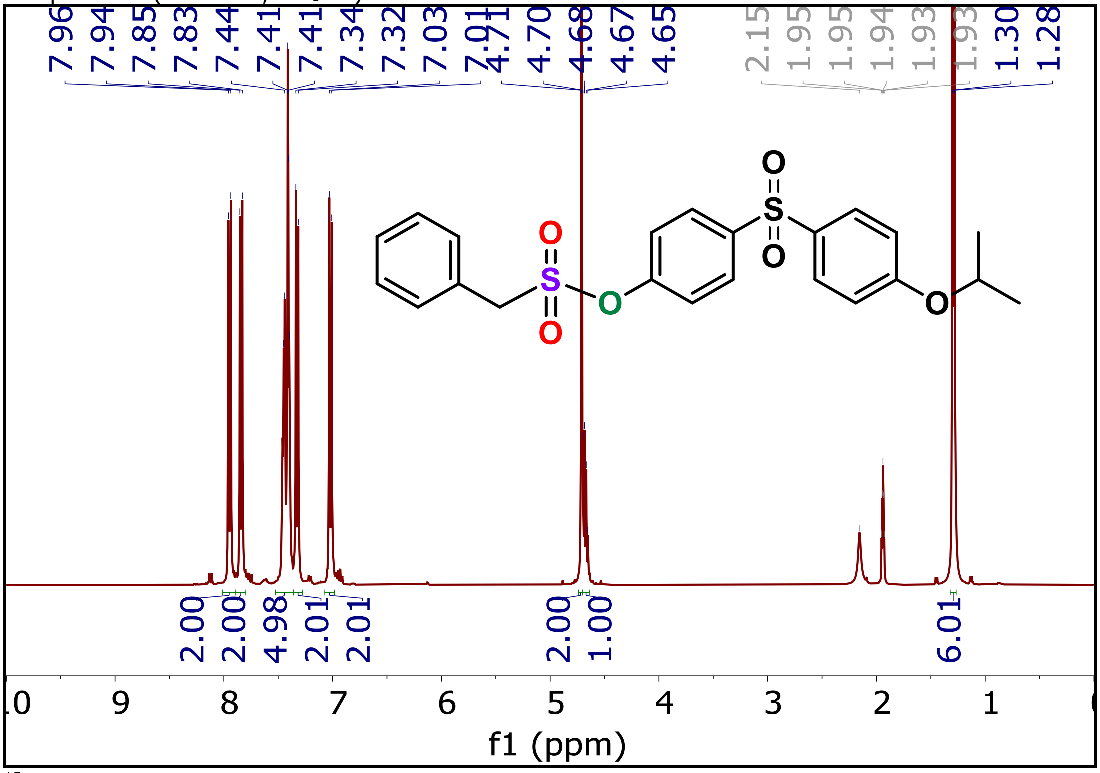

^{13}C spectrum (101 MHz, CD_3CN):

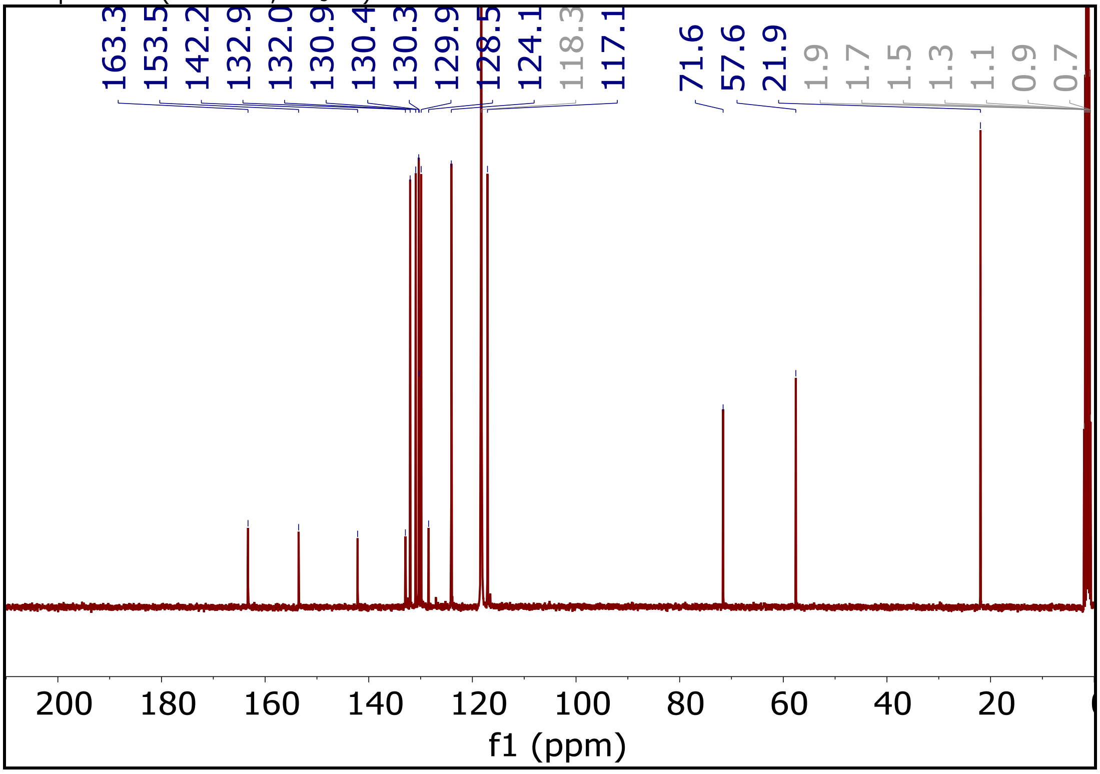

3ab. 1,3-Phenylene-bis(benzylsulfonate)

^{1}H spectrum (400 MHz, CD_3CN):

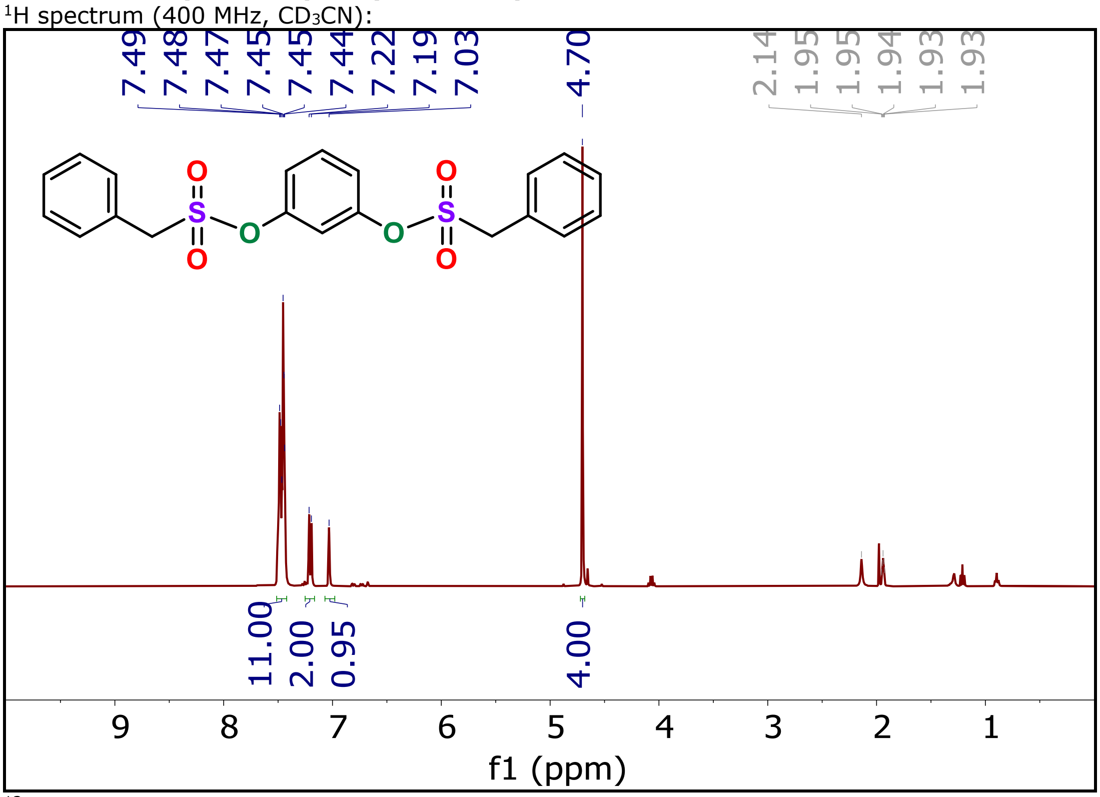

^{13}C spectrum (101 MHz, CD_3CN):

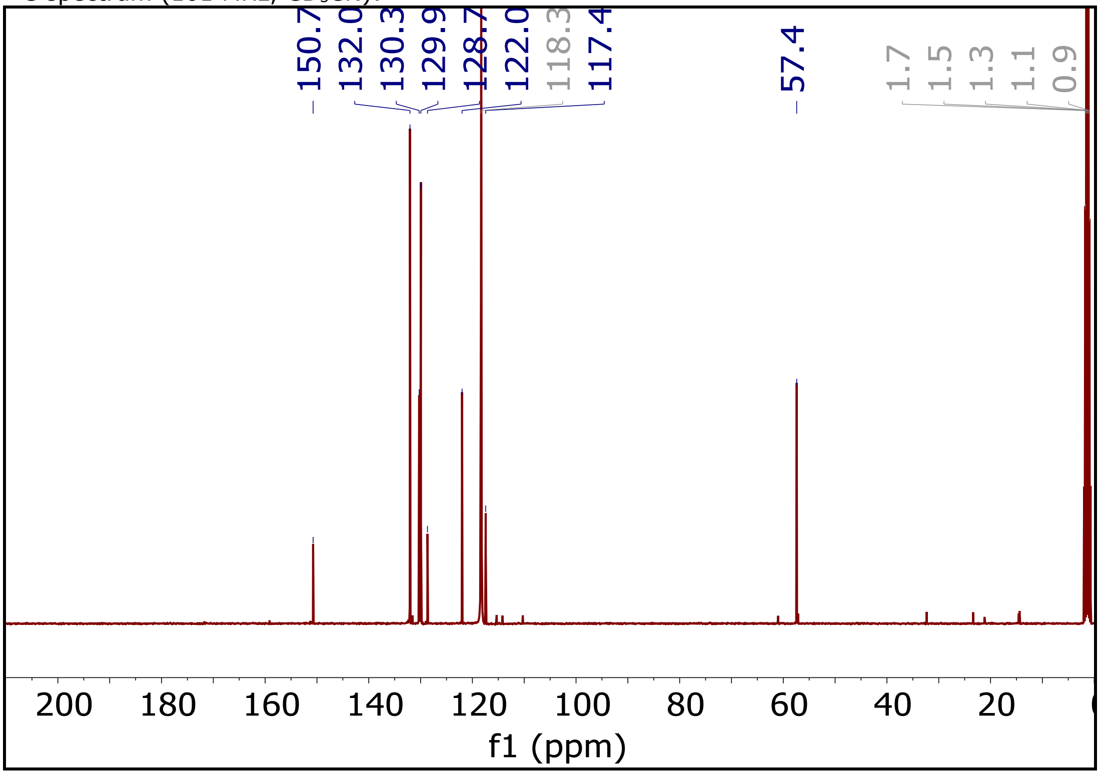

3ac. Propane-2,2-diylbis(4,1-phenylene) bis(benzylsulfonate)

^{1}H spectrum (400 MHz, CD_3CN):

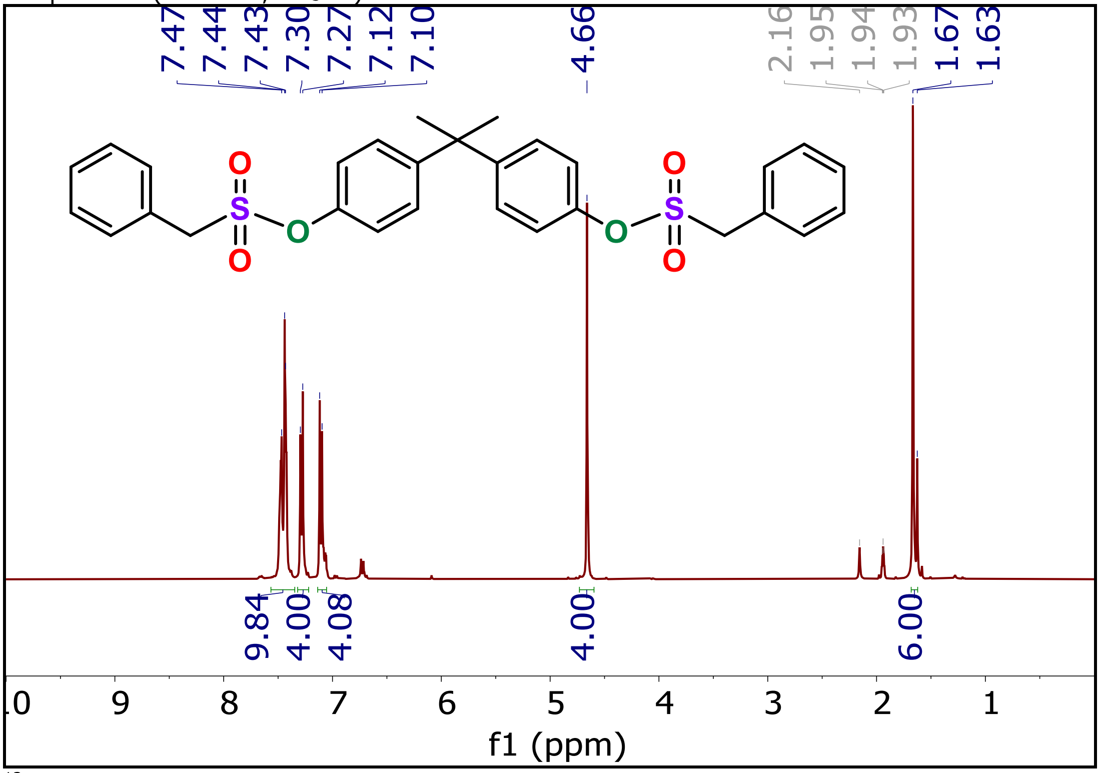

^{13}C spectrum (101 MHz, CD_3CN):

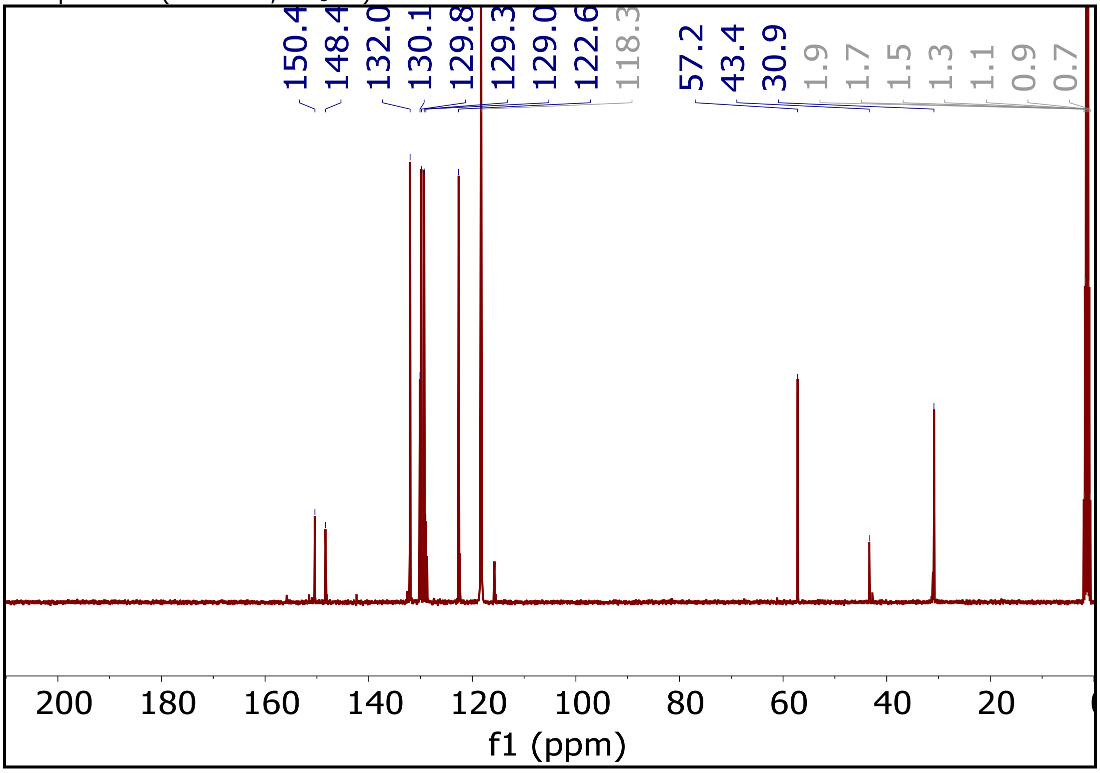

3ae. 2,6-Di-tert-butyl-4-methylphenyl benzylsulfonate

^{1}H spectrum (400 MHz, CD_3CN):

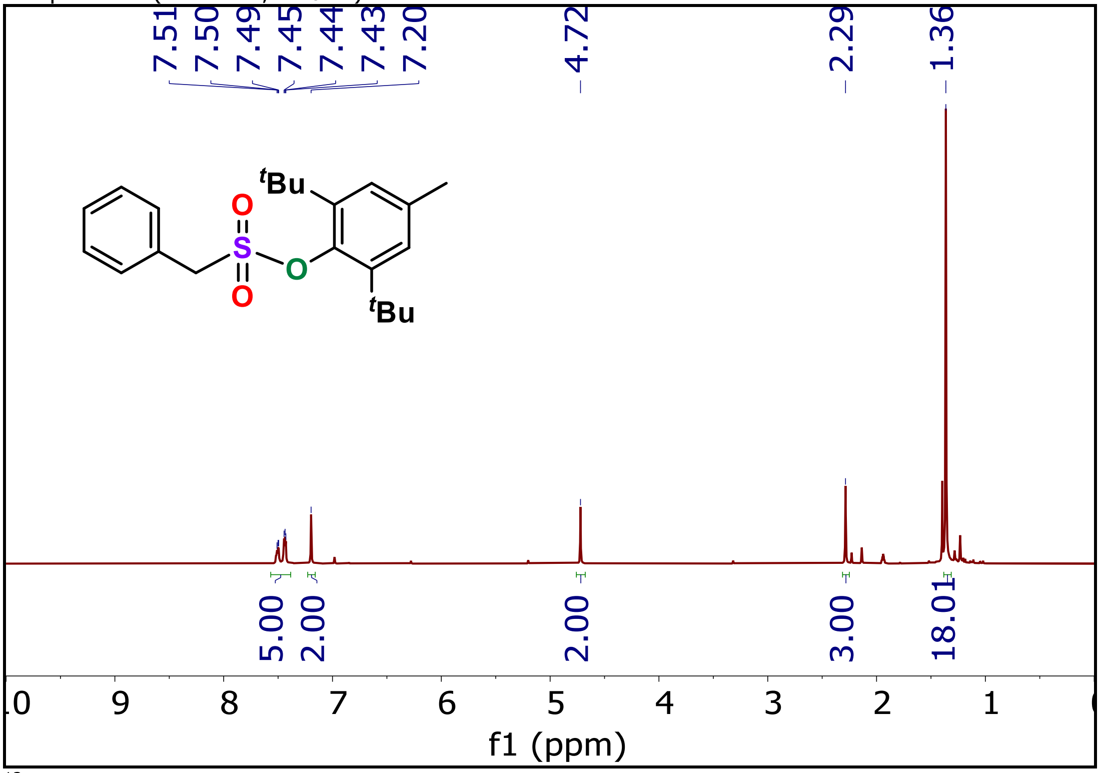

^{13}C spectrum (101 MHz, CD_3CN):

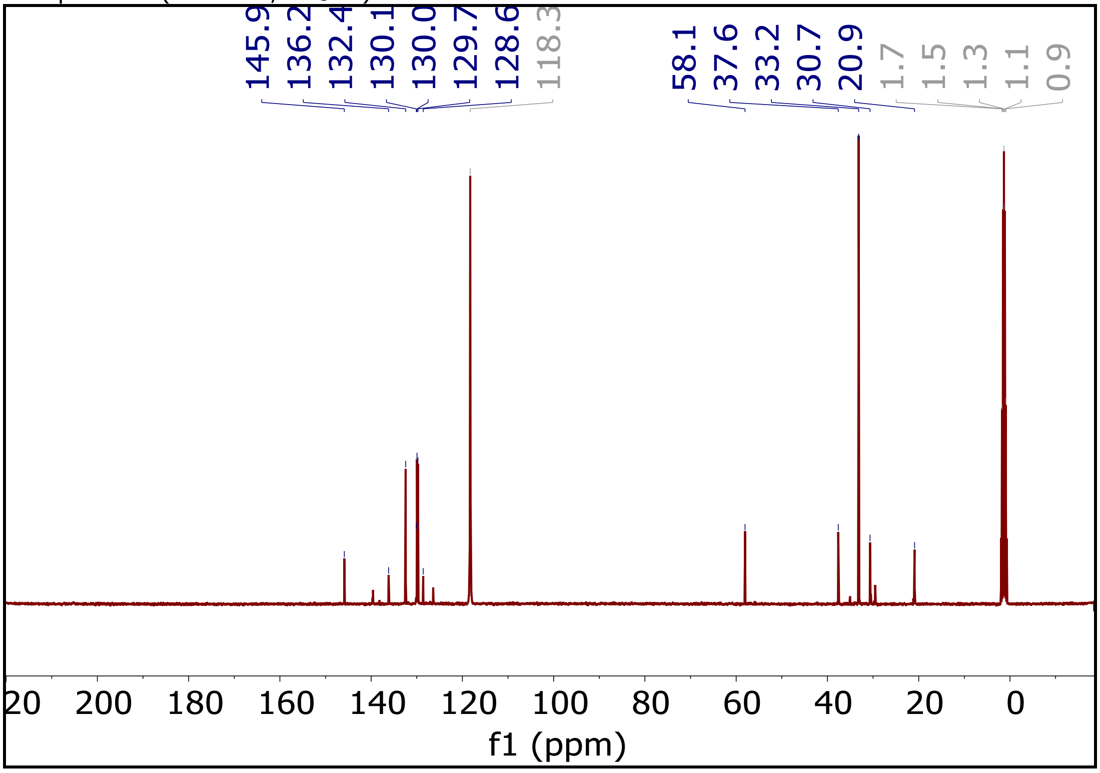

3af. 4-(Dimethylamino)phenyl benzylsulfonate

^{1}H spectrum (400 MHz, CD_3CN):

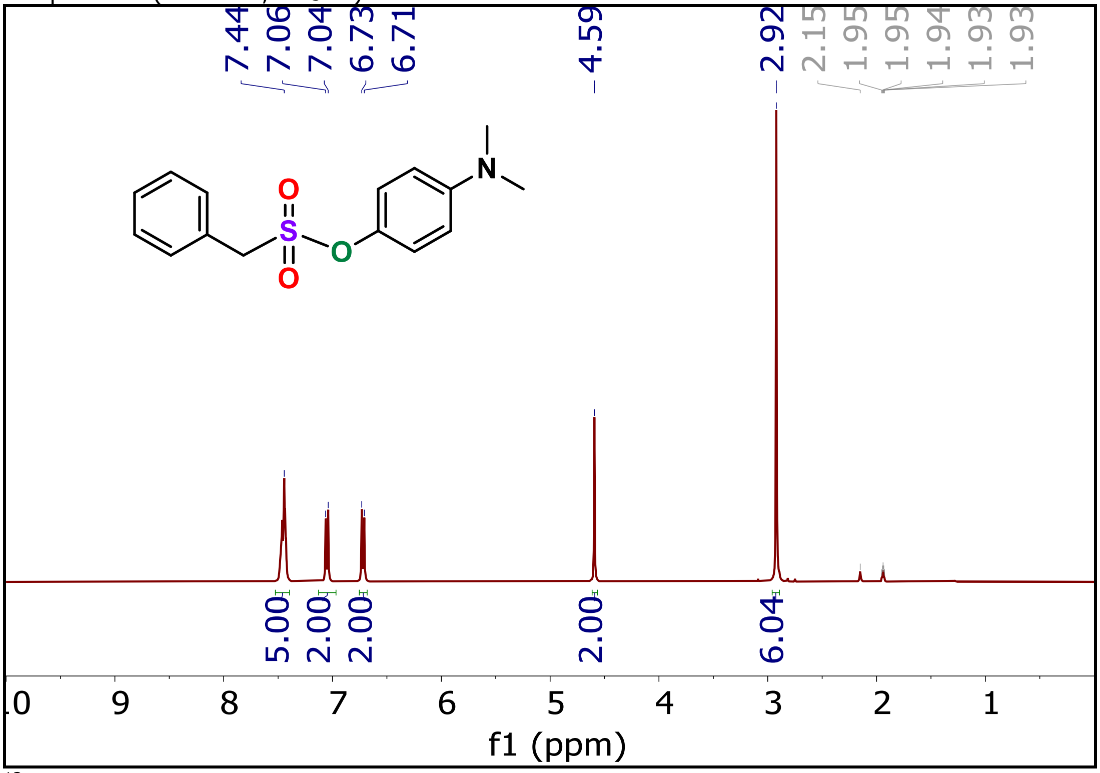

^{13}C spectrum (101 MHz, CD_3CN):

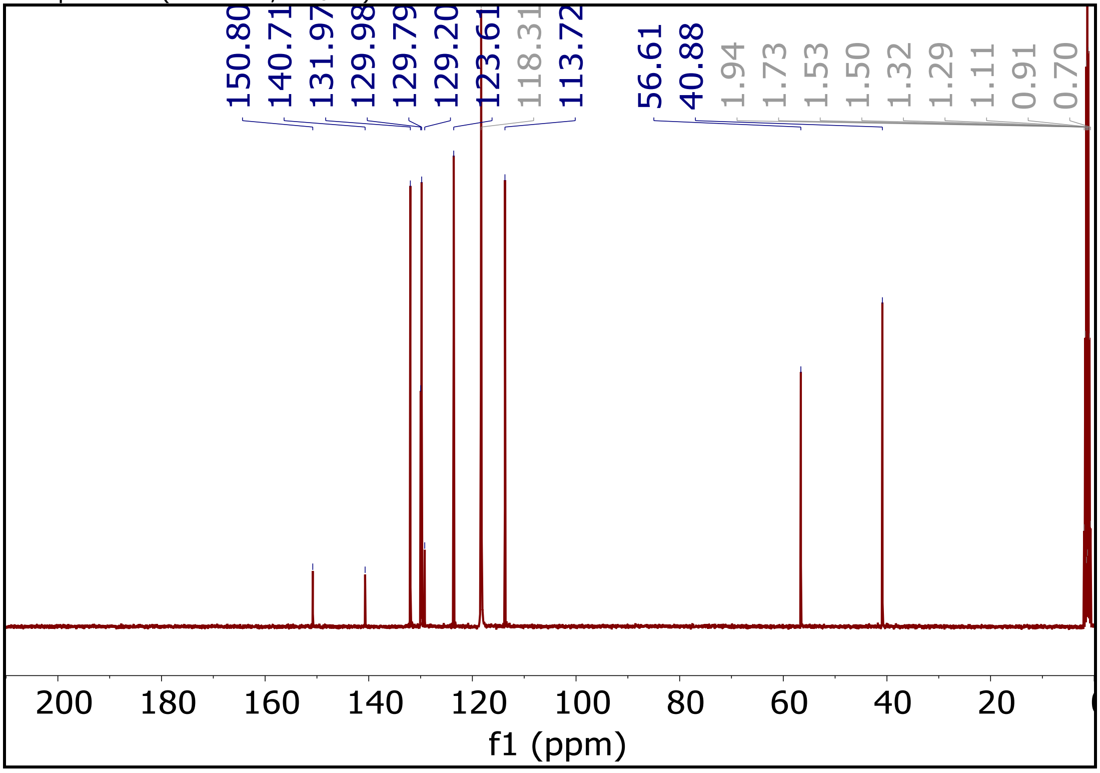

5a. 1-Butoxy benzylsulfonate

^{1}H spectrum (400 MHz, CD_3CN):

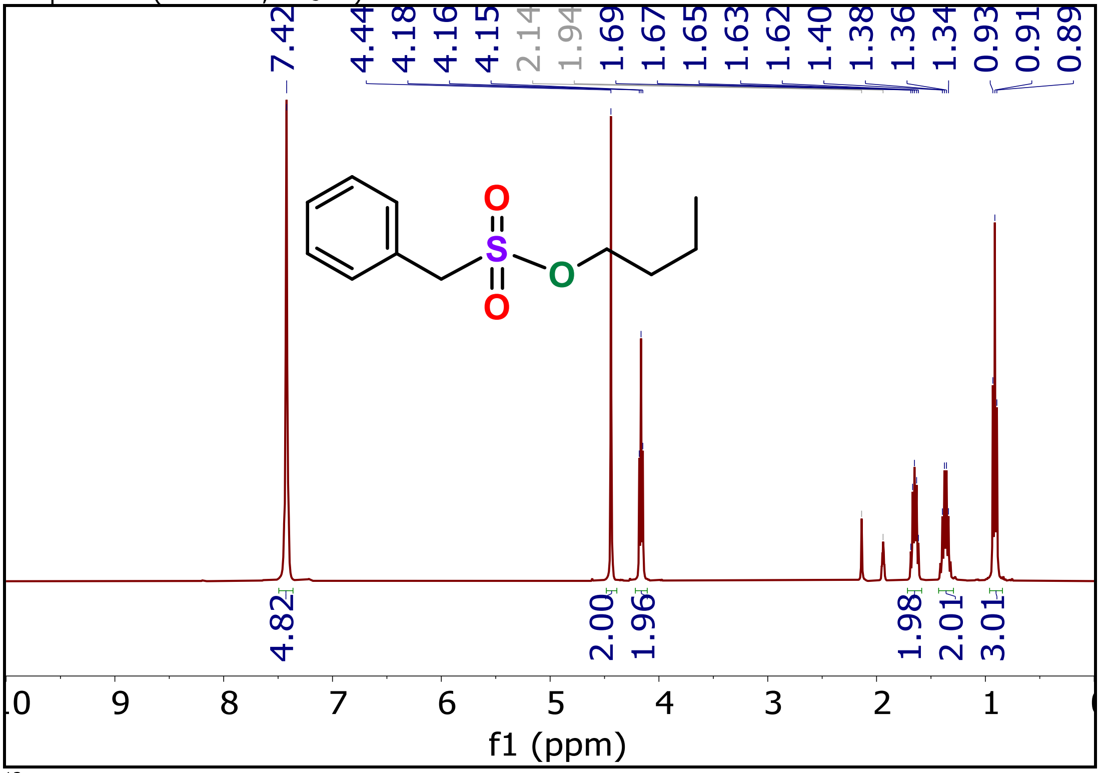

^{13}C spectrum (101 MHz, CD_3CN):

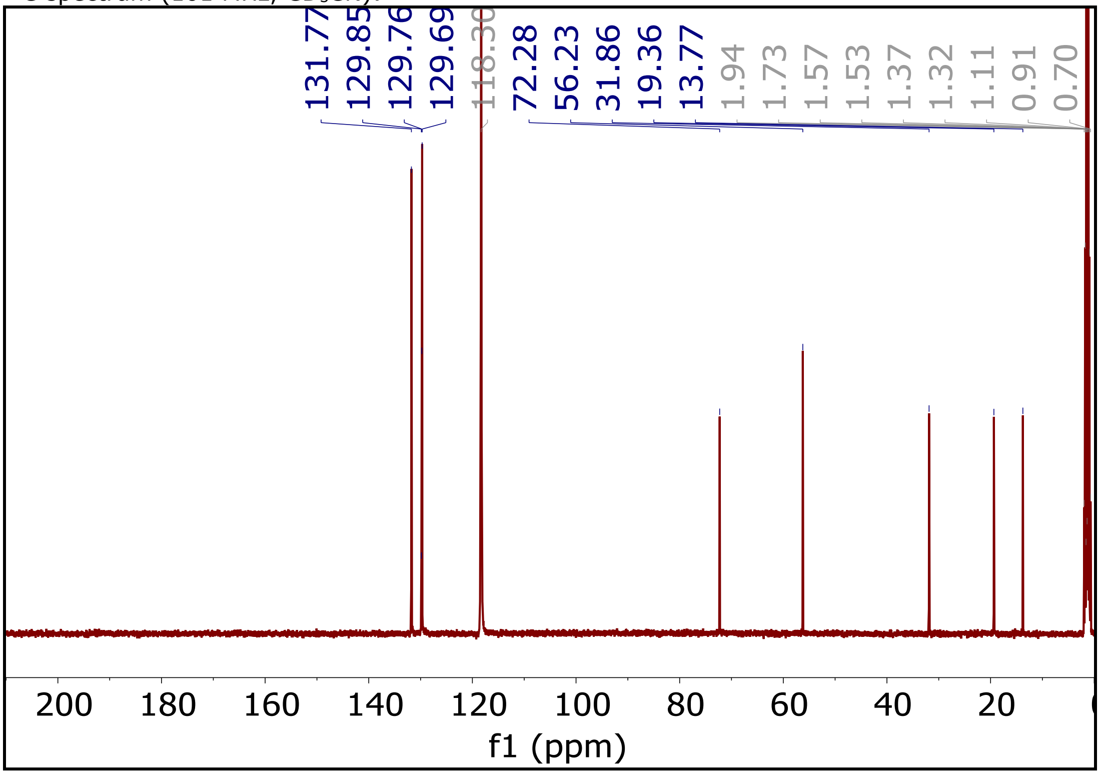

5b. 2-Butoxy benzylsulfonate

^{1}H spectrum (400 MHz, CD_3CN):

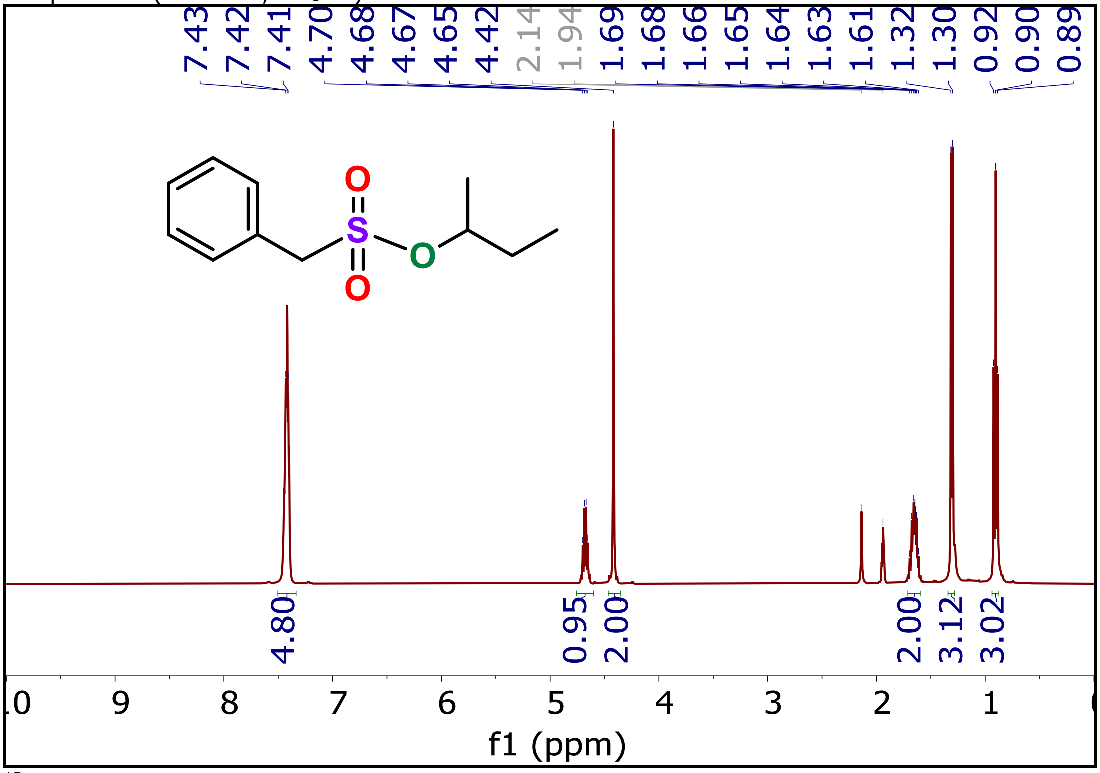

^{13}C spectrum (101 MHz, CD_3CN):

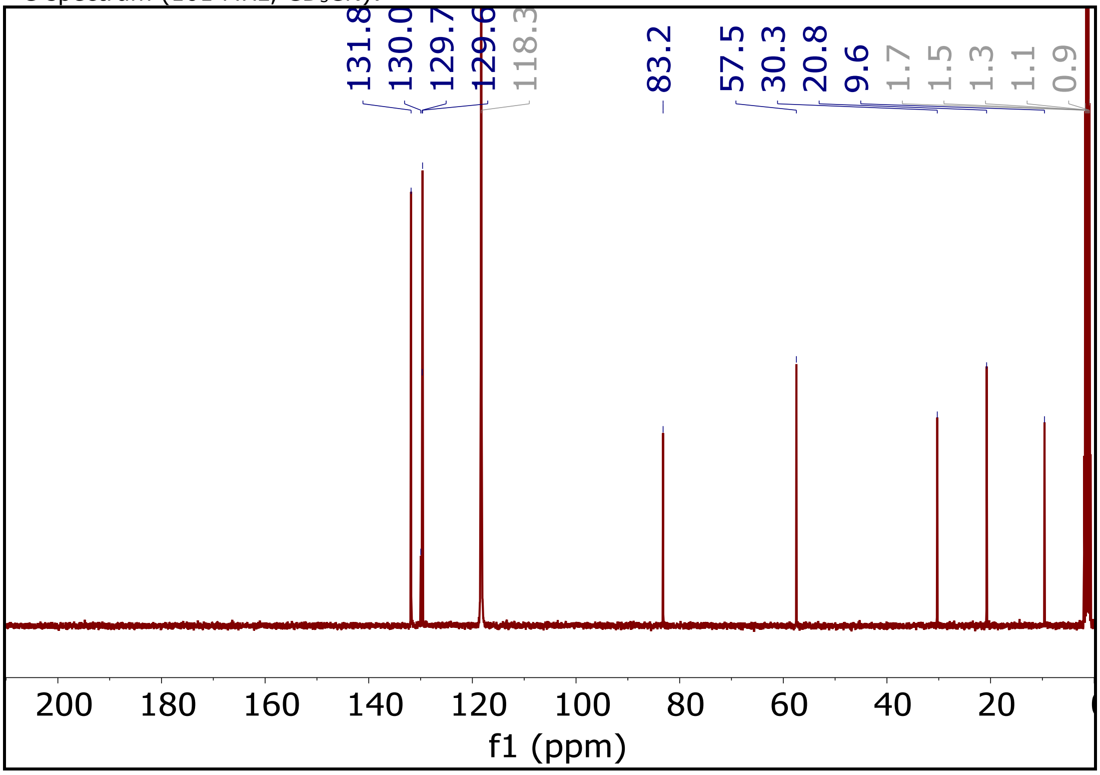

5f. Propane-1,2,3-triyl tris(benzylsulfonate)

^{1}H spectrum (400 MHz, CD_3CN):

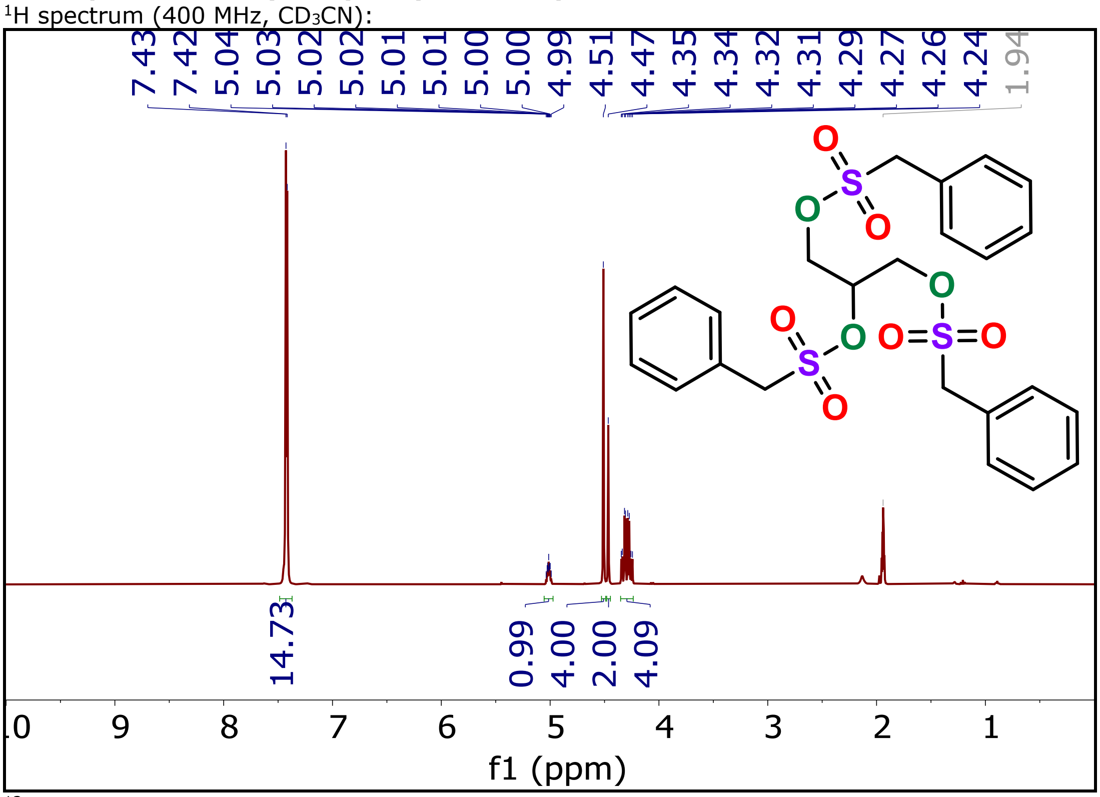

^{13}C spectrum (101 MHz, CD_3CN):

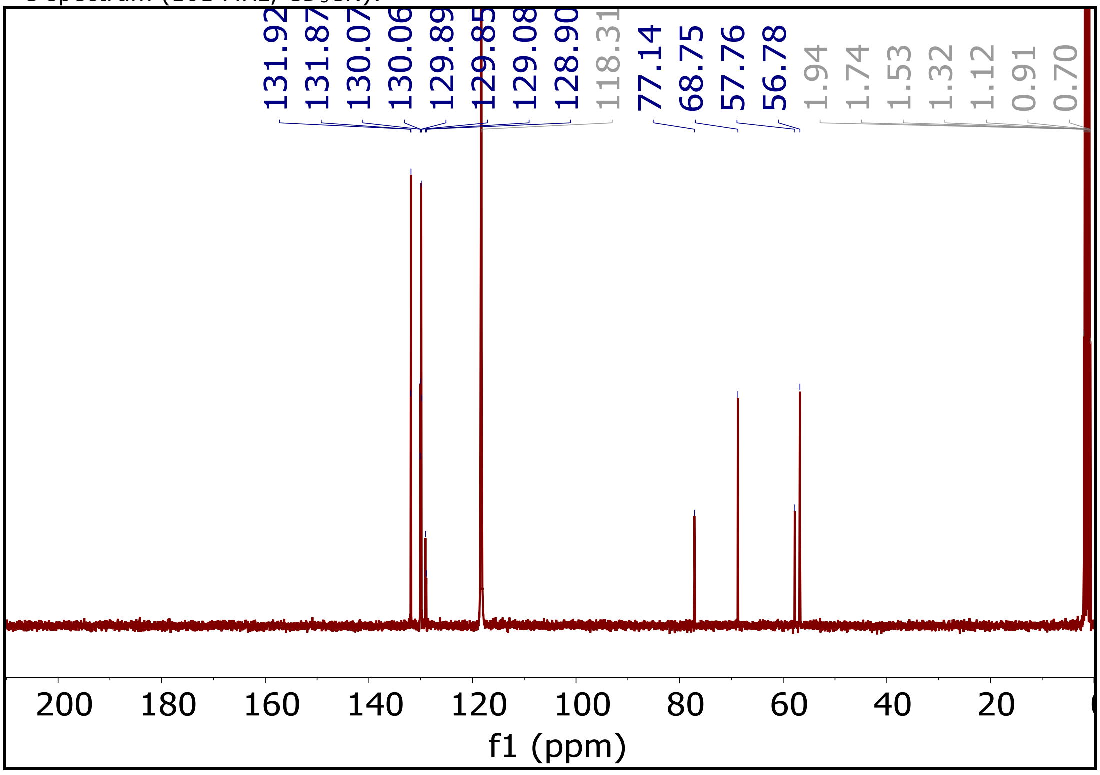

7a. 2-Isopropyl-5-methylphenyl benzylsulfonate

^{1}H spectrum (400 MHz, CD_3CN):

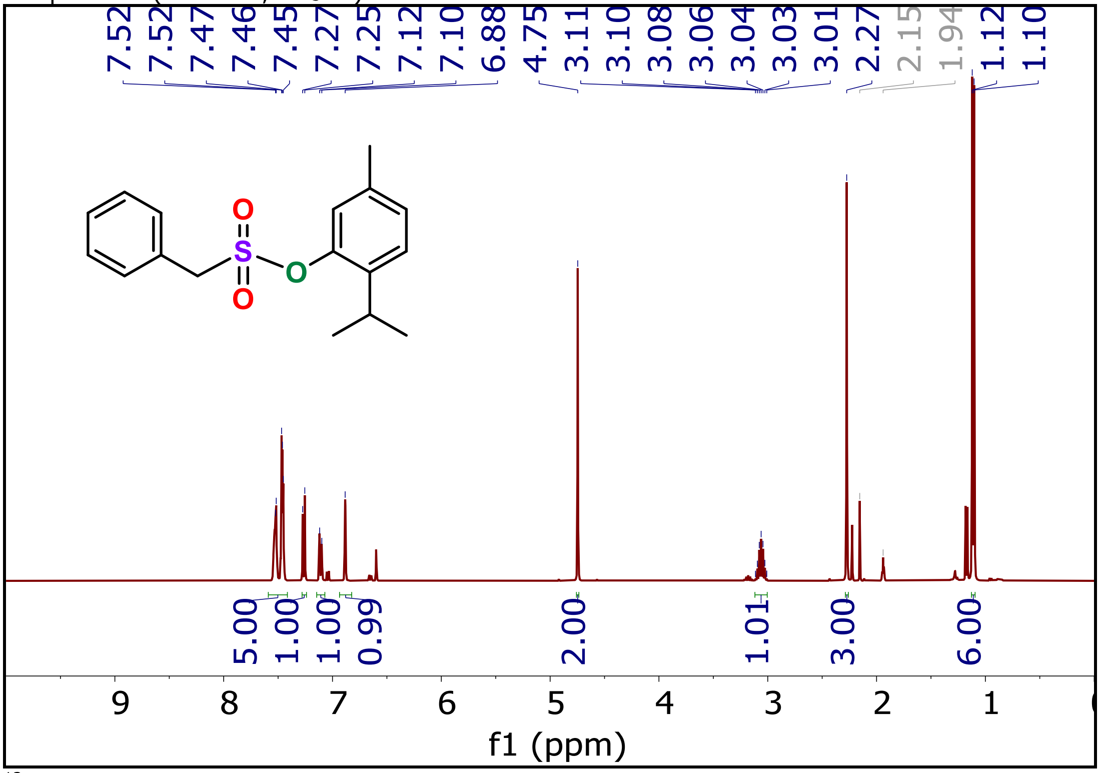

^{13}C spectrum (101 MHz, CD_3CN):

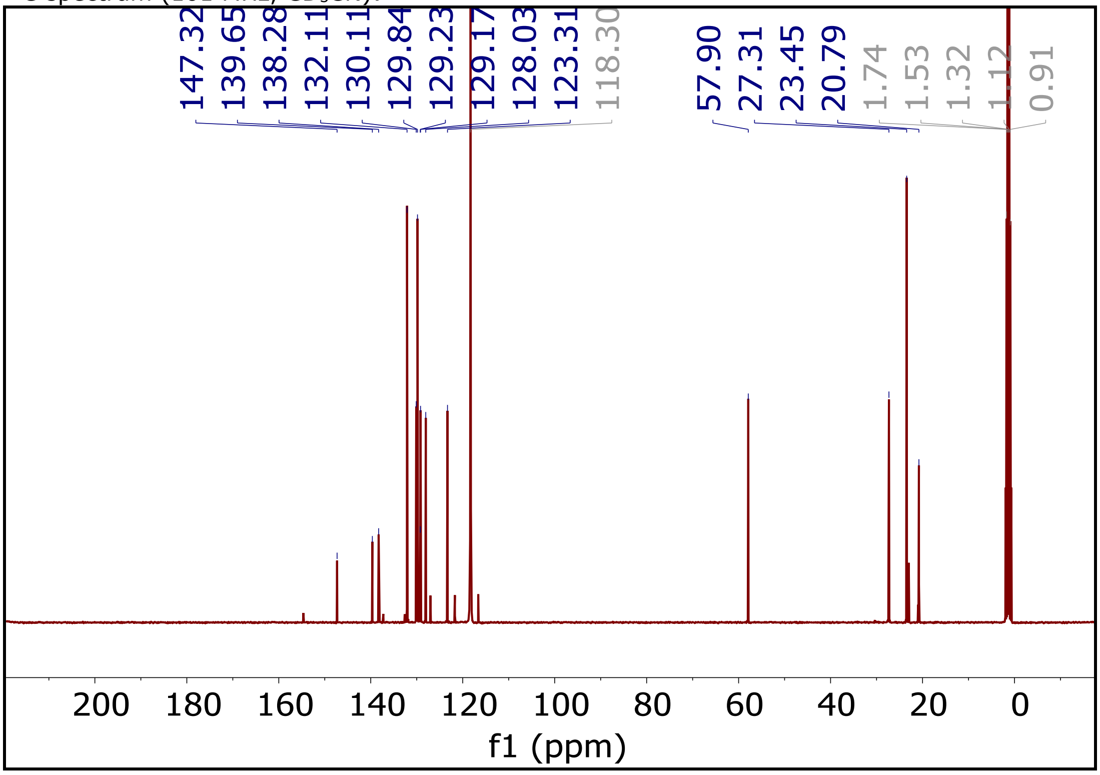

7b. Benzo[*d*][1,3]dioxol-5-yl benzylsulfonate

^{1}H spectrum (400 MHz, CD_3CN):

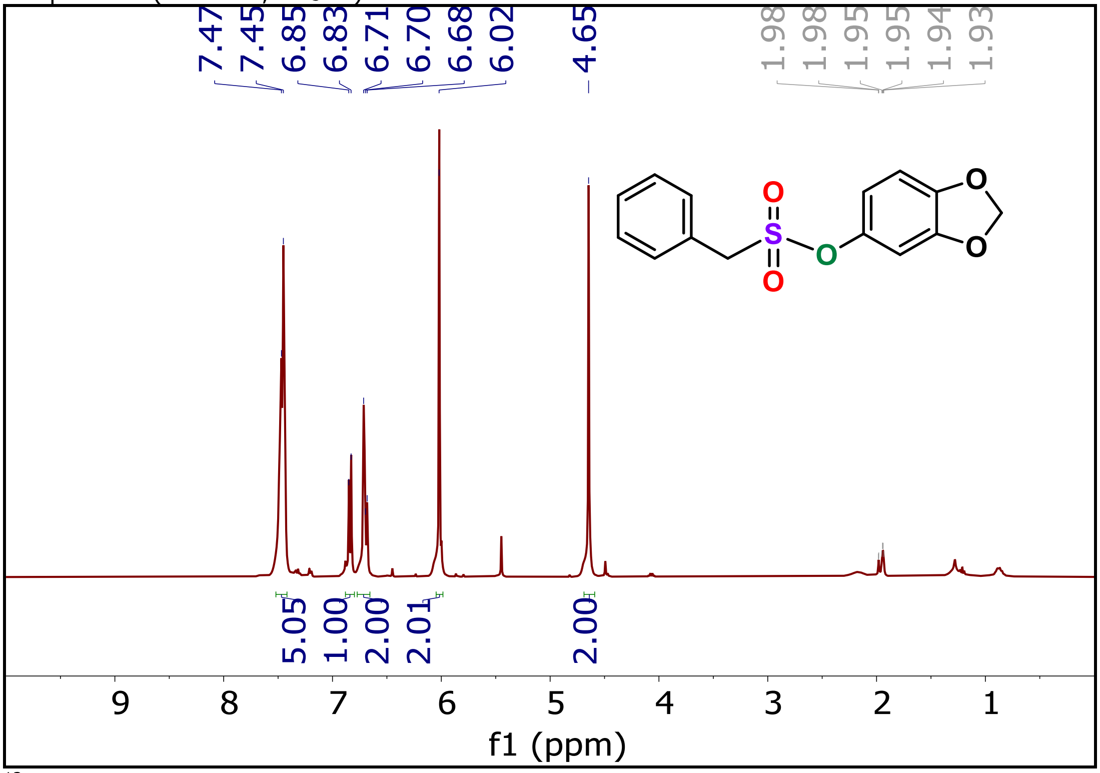

^{13}C spectrum (101 MHz, CD_3CN):

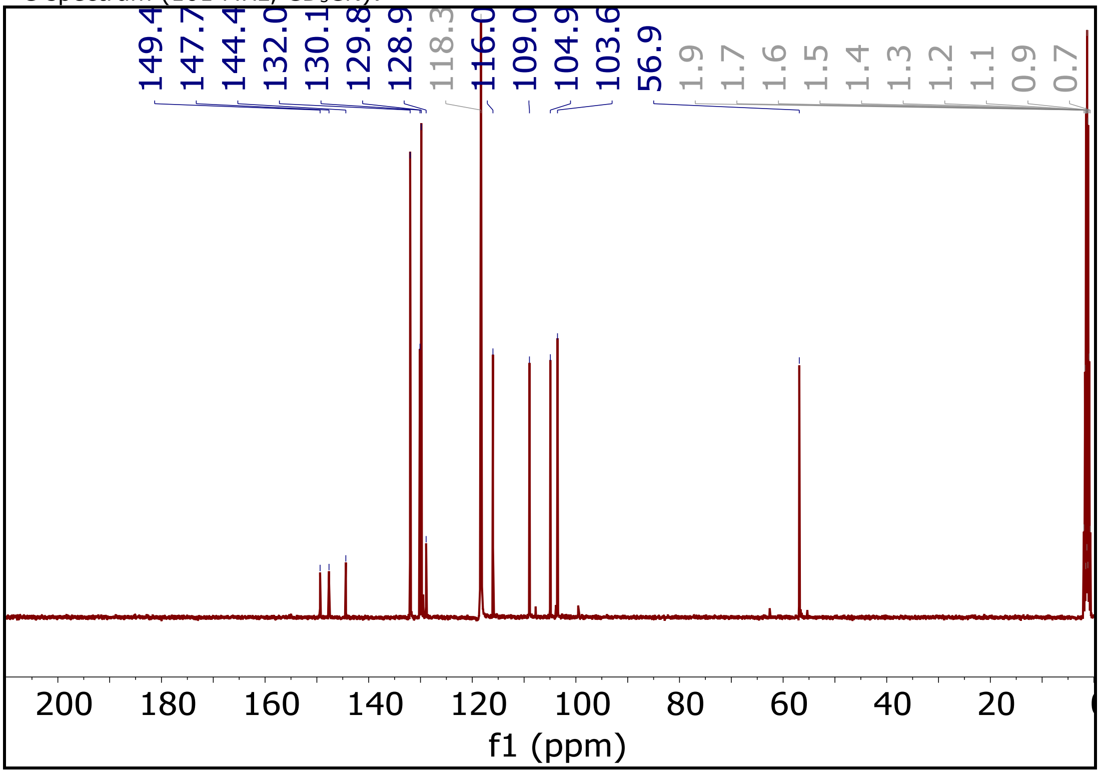

7c. 4-Allyl-2-methoxyphenyl benzylsulfonate

^{1}H spectrum (400 MHz, CD_3CN):

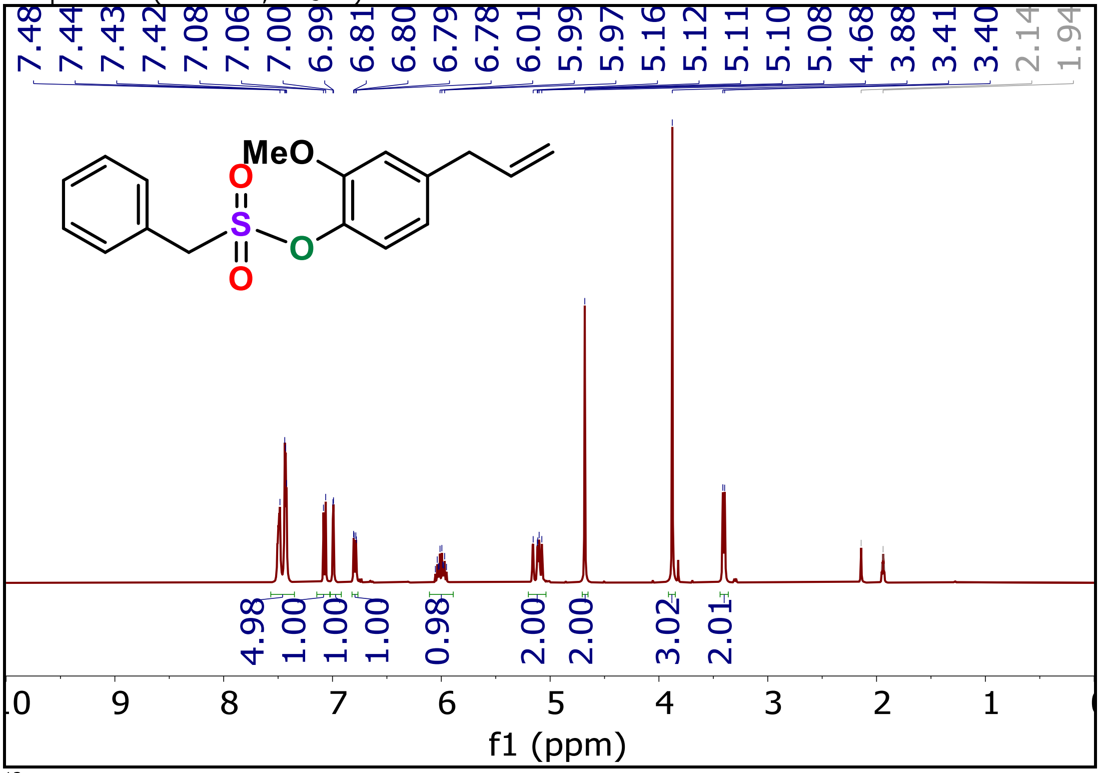

^{13}C spectrum (101 MHz, CD_3CN):

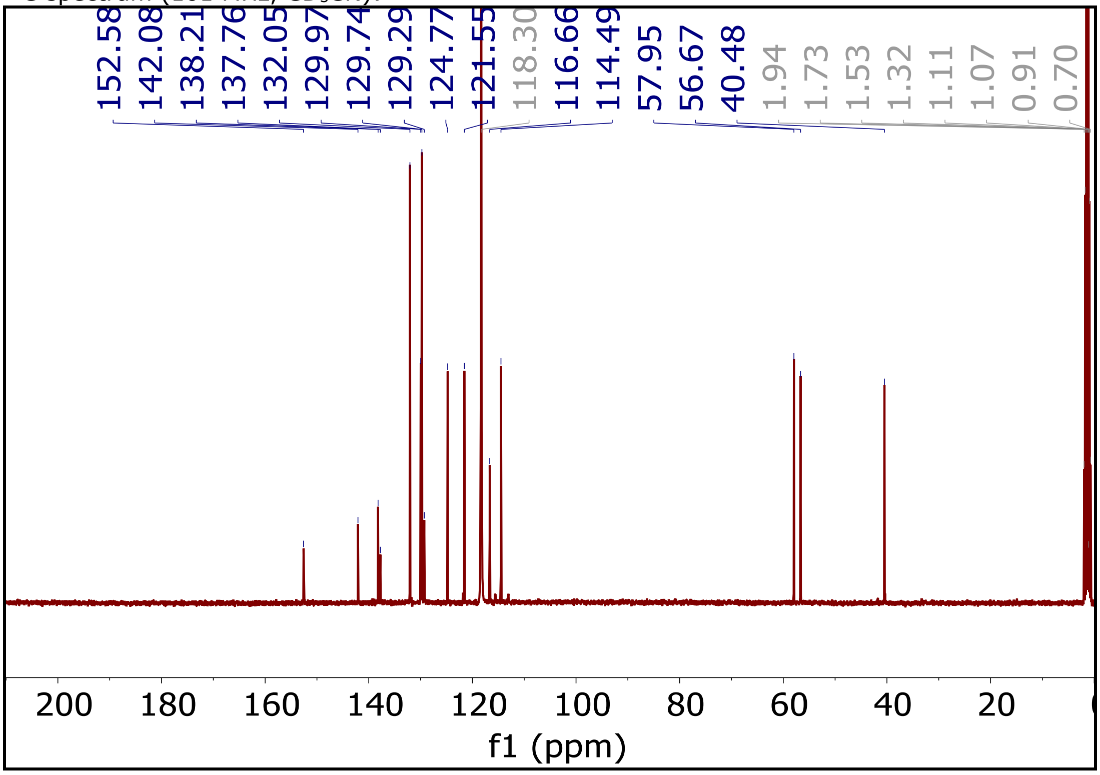

7e. 4-(2-Hydroxyethyl)phenyl benzylsulfonate

^{1}H spectrum (400 MHz, $CDCl_3$):

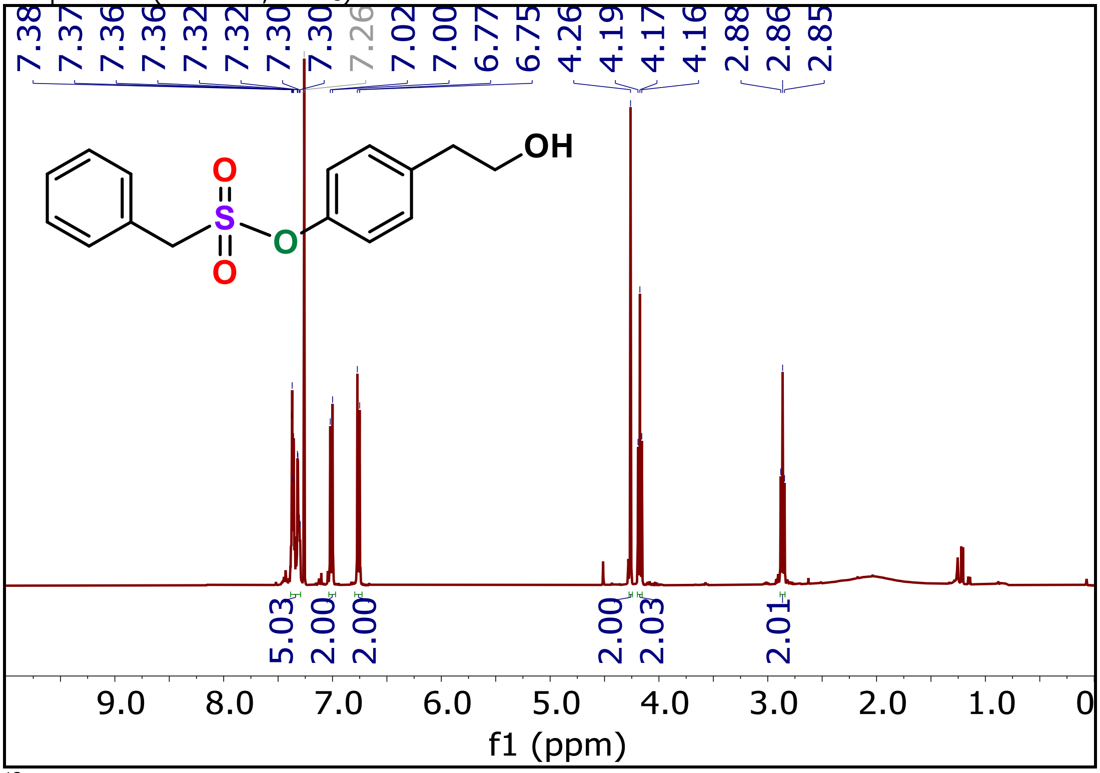

^{13}C spectrum (101 MHz, $CDCl_3$):

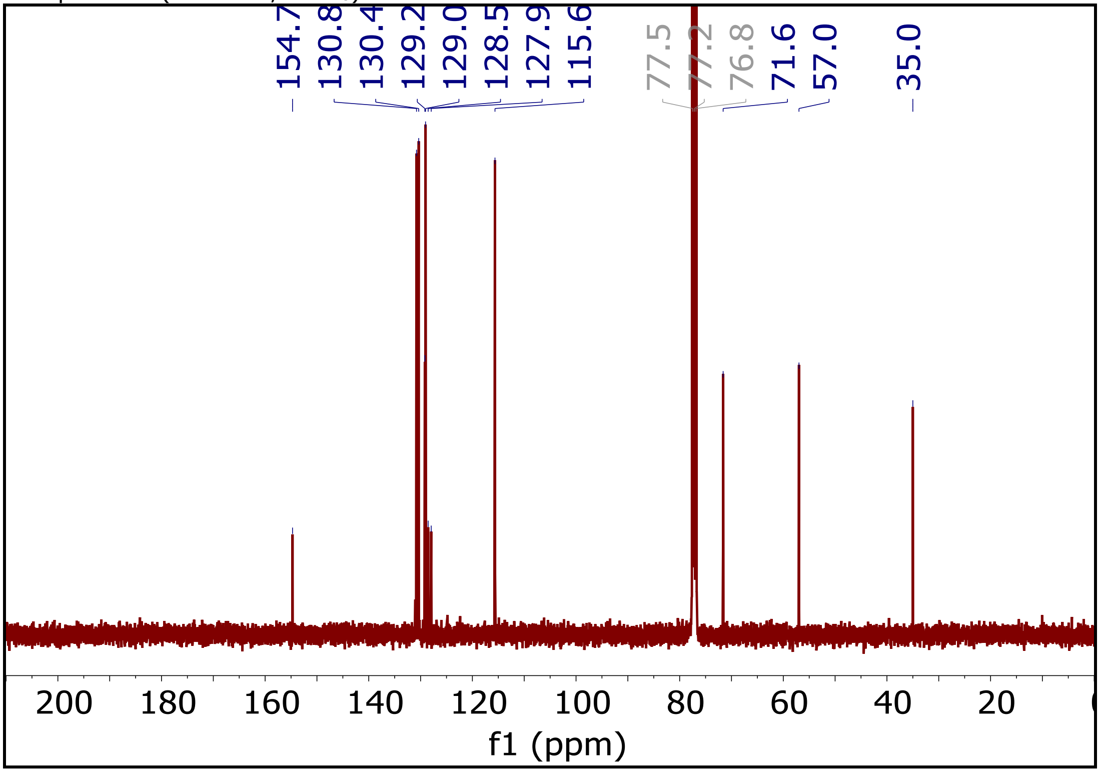

7g. 4-(3-Oxobutyl)phenyl benzylsulfonate

^{1}H spectrum (400 MHz, CD_3CN):

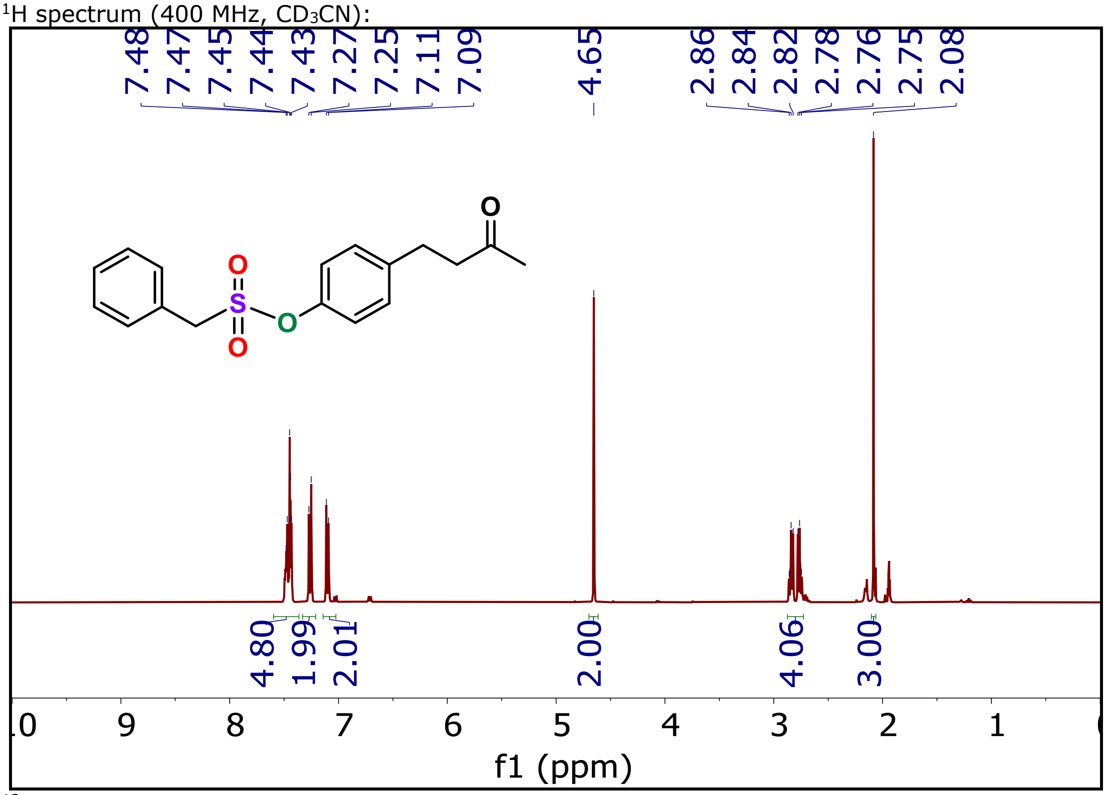

^{13}C spectrum (101 MHz, CD_3CN):

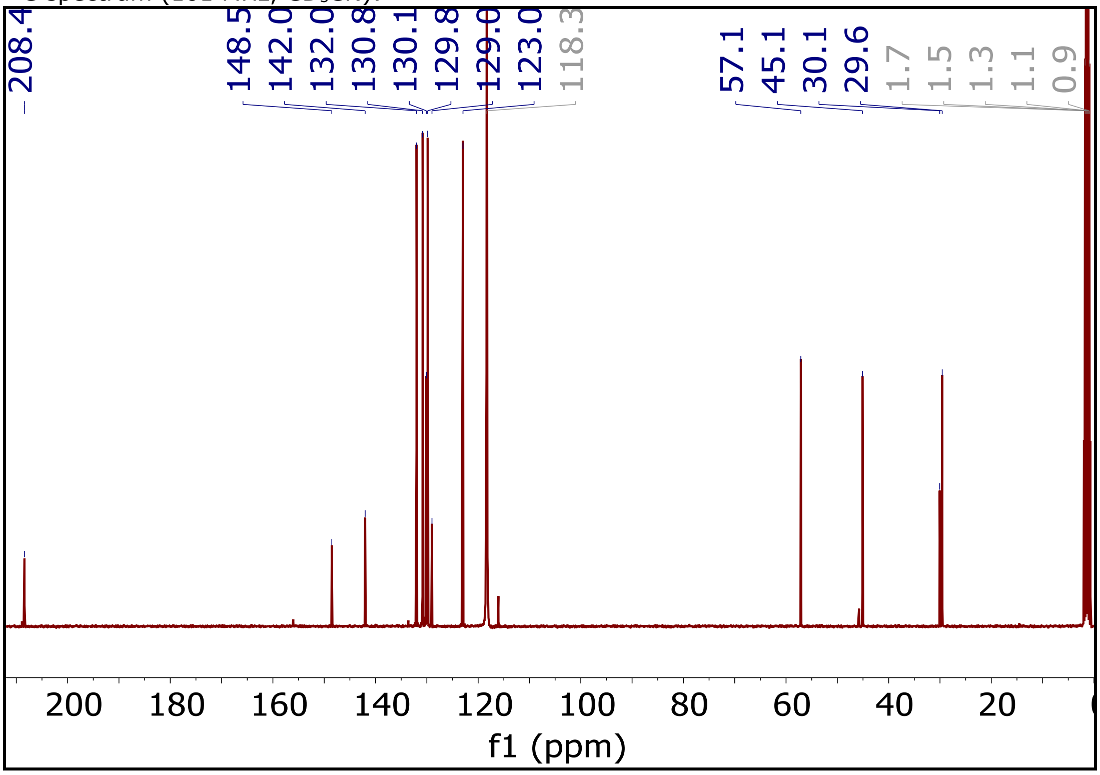

7i. β-Estradiol, benzylsulfonyl ester

^{1}H spectrum (400 MHz, $CDCl_3$):

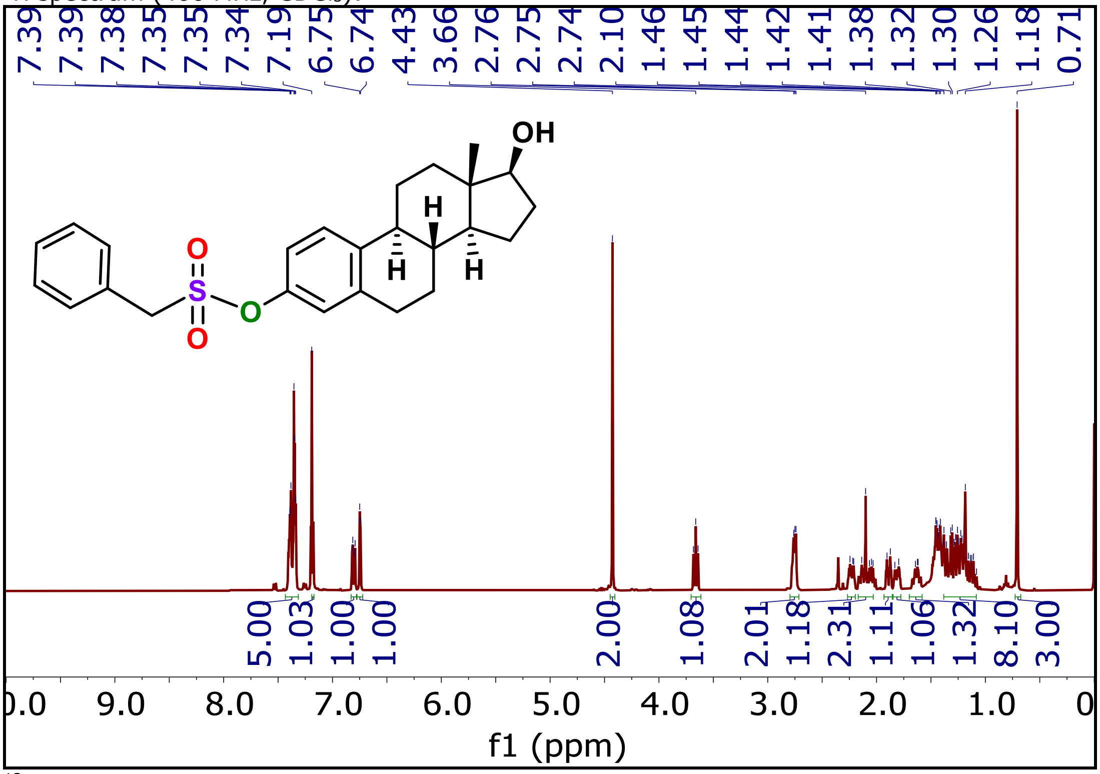

^{13}C spectrum (101 MHz, $CDCl_3$):

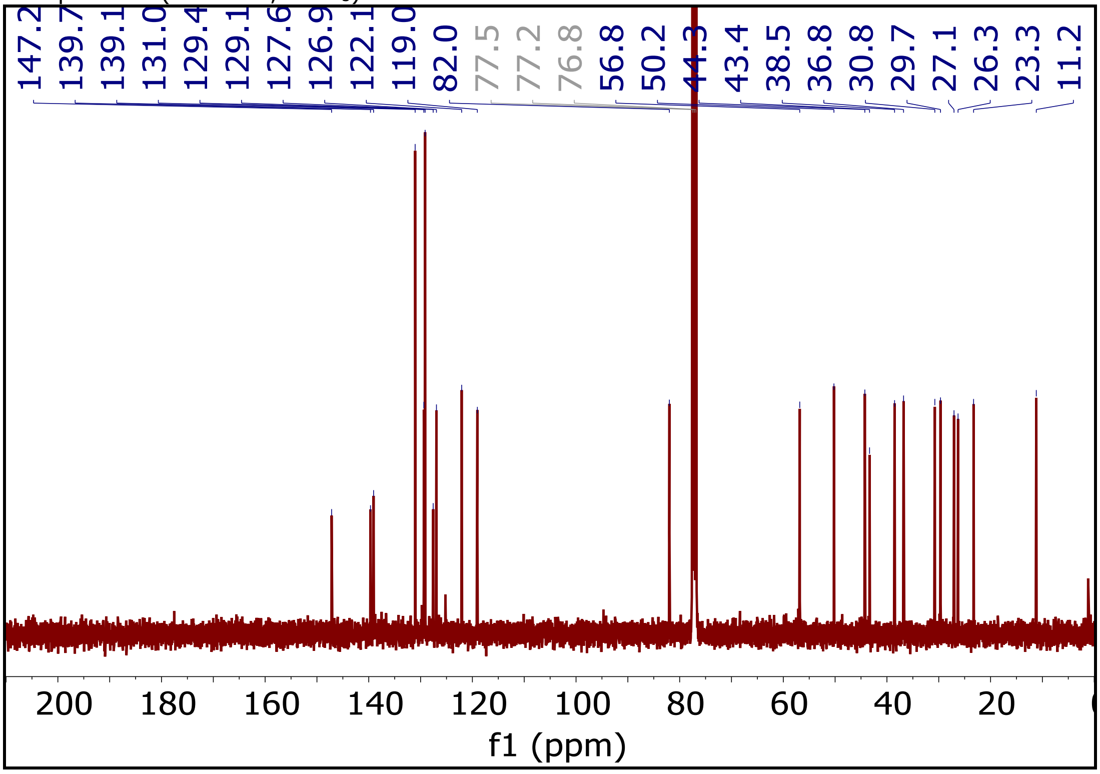

GPC distribution plots

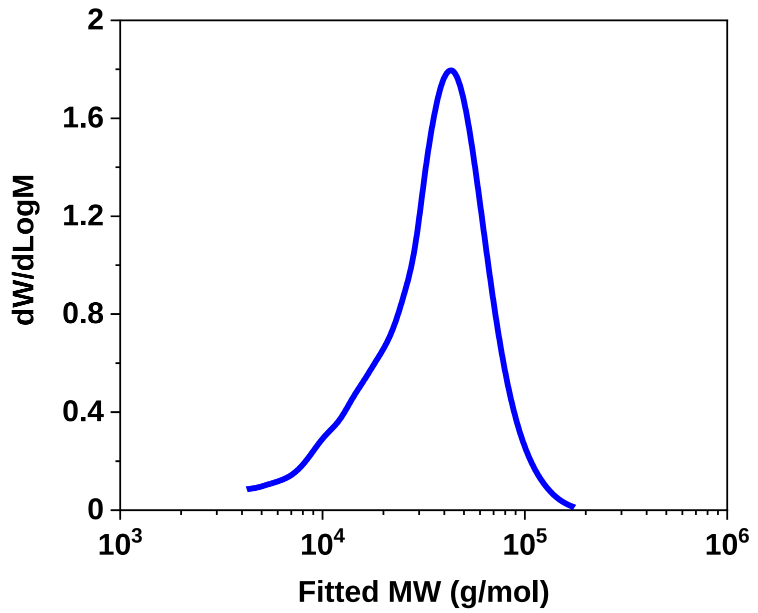

Molecular Weight Averages

Peak	Mp (g/mol)	Mn (g/mol)	Mw (g/mol)	Mz (g/mol)	Mz+1 (g/mol)	Mv (g/mol)	PD
Peak 1	43179	26575	40891	54573	68197	52633	1.539

GPC distribution plot and analyzed data for polysulfonate 8

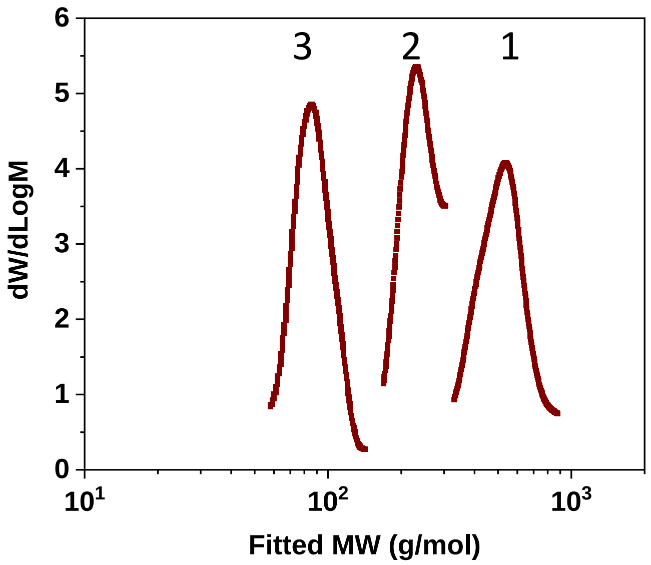

Molecular Weight Averages

Peak	Mp (g/mol)	Mn (g/mol)	Mw (g/mol)	Mz (g/mol)	Mz+1 (g/mol)	Mv (g/mol)	PD
Peak 1	555	509	534	559	584	555	1.049
Peak 2	238	228	232	236	240	235	1.018
Peak 3	87	85	87	90	92	89	1.024

GPC distribution plot for degraded polysulfonate 8.

LC Mass Spectra of the degraded polysulfonate **8**

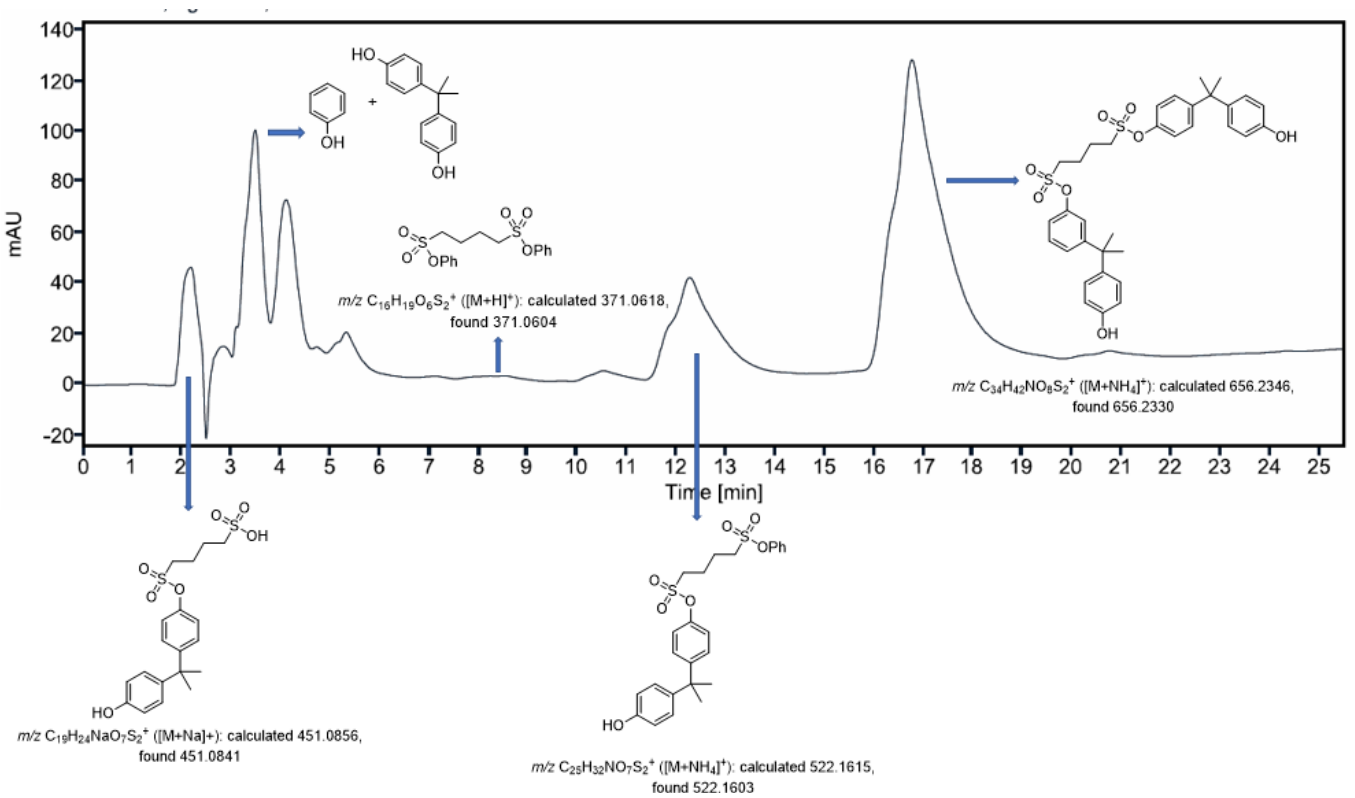

References

(1) Zaborsky, O. R.; Kaiser, E. T. A Comparative Study of the Alkaline Hydrolysis of O-Hydroxy-α-Toluenesulfonic Acid Sultone and Phenyl α-Toluenesulfonate. *J. Am. Chem. Soc.* **1966**, *88* (13), 3084–3087.

(2) Alonso, F.; Moglie, Y.; Vitale, C.; Radivoy, G.; Yus, M. A New Mild Deprotecting Method for O-Benzylsulfonyl Phenols and Alcohols Based on a DTBB-Catalyzed Lithiation. *Synthesis (Stuttg).* **2005**, No. 12, 1971–1976.

(3) Moghaddam, F. M.; Hoor, A. A.; Dekamin, M. G. Microwave-Promoted Pseudo-Thia-Fries Rearrangement of Aryl Benzylsulfonates; Highly Reactive Benzyl Cation Generation. *J. Sulfur Chem.* **2004**, *25* (2–3), 125–130.

(4) Truce, W. E.; Christensen, L. W. Base-Induced a-Sulfonylation of Aryl Alkanesulfonates. *J. Org. Chem.* **1970**, *35* (11), 3968–3970.

(5) Betts, L. M.; Tam, N. C.; Kabir, S. M. H.; Langler, R. F.; Crandall, I. Ether Aryl Sulfonic Acid Esters with Improved Antimalarial/Anticancer Activities. *Aust. J. Chem.* **2006**, *59* (4), 277–282.

(6) Gao, J.; Pan, X.; Liu, J.; Lai, J.; Chang, L.; Yuan, G. Iodine-Induced Synthesis of Sulfonate Esters from Sodium Sulfinates and Phenols under Mild Conditions. *RSC Adv.* **2015**, *5* (35), 27439–27442.

(7) Davy, M. B.; Douglas, K. T.; Loran, J. S.; Steltner, A.; Williams, A. Elimination-Addition Mechanisms of Acyl Group Transfer: Hydrolysis and Aminolysis of Aryl Phenylmethanesulfonates. *J. Am. Chem. Soc.* **1977**, *99* (4), 1196–1206.

(8) Smedley, C. J.; Homer, J. A.; Gialelis, T. L.; Barrow, A. S.; Koelln, R. A.; Moses, J. E. Accelerated SuFEx Click Chemistry For Modular Synthesis. *Angew. Chem. Int. Ed.* **2022**, *61* (4).

(9) Bahrami, K.; Khodaei, M. M.; Abbasi, J. Synthesis of Sulfonamides and Sulfonic Esters via Reaction of Amines and Phenols with Thiols Using H2O2-POCl3 System. *Tetrahedron* **2012**, *68* (25), 5095–5101.

(10) Dai, Q.; Liu, L.; Zhang, J. Palladium / Xiao-Phos-Catalyzed Kinetic Resolution of Sec -Phosphine Oxides by P -Benzylation. *Angew. Chem. Int. Ed.* **2021**, *60*, 27247–27252.

(11) Gilman, H.; Beaber, N. J.; Myers, C. H. THE REACTION BETWEEN ARYL SULFONATES AND ORGANOMAGNESIUM HALIDES. *J. Am. Chem. Soc.* **1925**, *47* (7), 2047–2052.

(12) Borrows, E. T.; Clayton, J. C.; Hems, B. A.; Long, A. G. S 43. The Synthesis of Thyroxine and Related Substances. Part II. The Preparation of Dinitrodiphenyl Ethers. *J. Chem. Soc.* **1949**, No. 0, S190–S199.

(13) Takikawa, Y.; Saburo, T. Reactions of Organic Compounds with Sodium Hydrosulfide in Liquid Ammonia. IV. Reactions of Aromatic Nitriles with Sodium Hydrosulfide in Liquid Ammonia. *Nippon Kagaku Kaishi* **1972**, *1972* (4), 766–770.

(14) Kenyon, J.; Phillips, H.; Pittman, V. P. 250. Walden Inversion Reactions of d-β-Butyl, d-β-Octyl, and d-Benzylmethylcarbinyl p-Toluenesulphonates. *J. Chem. Soc.* **1935**, No. 0, 1072–1084.

(15) Gao, B.; Zhang, L.; Zheng, Q.; Zhou, F.; Klivansky, L. M.; Lu, J.; Liu, Y.; Dong, J.; Wu, P.; Sharpless, K. B. Bifluoride-Catalysed Sulfur(VI) Fluoride Exchange Reaction for the Synthesis of Polysulfates and Polysulfonates. *Nat. Chem.* **2017**, *9* (11), 1083–1088.
